# An optimized protocol for efficient derivation of pancreatic islets from multiple human pluripotent stem cell lines

**DOI:** 10.1016/j.stemcr.2026.102892

**Published:** 2026-04-16

**Authors:** Siqin Wu, Shivam Chandel, Galyna Bryzgalova, Paschalis Efstathopoulos, Kelly Blust, Cheng Zhao, Eda Erbil, Anna Falk, My Hedhammar, Per-Olof Berggren, Fredrik Lanner

**Affiliations:** 1 Department of Clinical Sciences, Intervention and Technology, Karolinska Institutet, 171 77 Stockholm, Sweden; 2 Gynecology and Reproductive Medicine, Karolinska Universitetssjukhuset, 141 86 Stockholm, Sweden; 3 Spiber Technologies AB, AlbaNova University Center, 106 91 Stockholm, Sweden; 4 The Rolf Luft Research Center for Diabetes and Endocrinology, Karolinska Institutet, 171 76 Stockholm, Sweden; 5 Division of Protein Technology, KTH Royal Institute of Technology, Roslagstullsbacken 21, 106 91 Stockholm, Sweden; 6 Department of Neuroscience, Karolinska Institutet, 171 65 Solna, Sweden; 7 Neural Stem Cells, Department of Experimental Medical Science, Lund Stem Cell Center, Lund University, 221 84 Lund, Sweden

**Keywords:** human pluripotent stem cells, beta cells, endocrine progenitors, differentiation protocol, functional SC-islets, endocrine purity, cell-line variability, cell therapy, diabetes, intraocular transplantation

## Abstract

The success of cell therapy for type 1 diabetes (T1D) depends on reliable differentiation of stem cells into functional pancreatic islets. Current protocols produce stem cell-derived islets (SC-islets) that contain non-endocrine cells and show limited maturity. We developed a robust protocol that generates functional SC-islets from all eight tested human pluripotent stem cell (hPSC) lines. Differentiation to the endocrine progenitor (EP) stage on 2D laminin-521 is improved by shortening the prior pancreatic progenitor (PP) stage. Notably, allowing EP cells to self-aggregate efficiently removes proliferative and non-endocrine cells. Subsequent suspension culture yields SC-islets with strong glucose responsiveness *in vitro*. After transplantation into the anterior chamber of the eye of diabetic mice, SC-islets further mature and restore normal glycemic control. Single-cell analyses show that the SC-islets are free of non-endocrine cell populations before and after transplantation. This protocol enables production of highly functional SC-islets suitable for T1D cell therapy.

## Highlights

• Differentiation to pancreatic endocrine progenitors is highly efficient on laminin-521
• Transfer from 2D to 3D culture yields functional SC-islets free of non-endocrine cells
• Glucose responsive SC-islets are generated from all tested hPSC lines
• The anterior chamber of the eye enables SC-islet transplantation and functional analysis

## Graphical abstract

**Figure:**
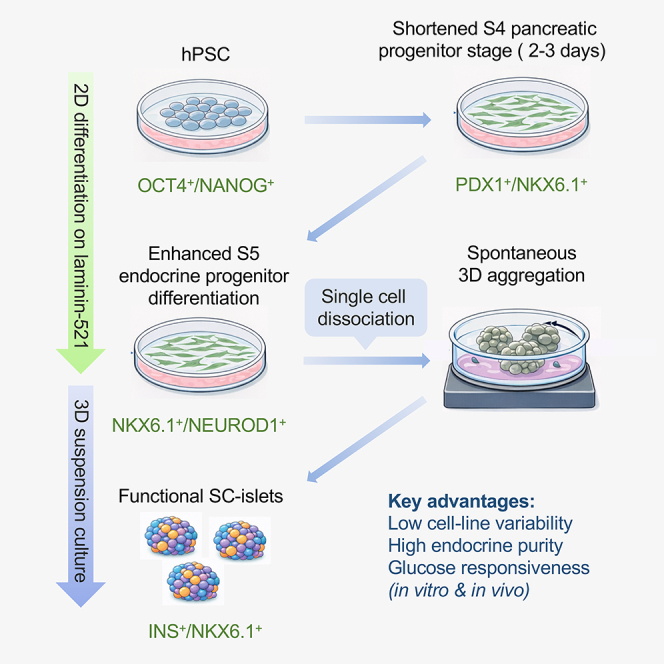

## Teaser

Wu et al. have established a new protocol for efficient generation of functional SC-islets from all hPSC lines tested in this study. The SC-islets display strong *in vitro* glucose responsiveness and high endocrine cell purity. Upon transplantation into the anterior chamber of the eye in mice, the SC-islets are able to reverse preexisting diabetes.

## Introduction

Pancreatic islets regulate blood glucose through coordinated hormone secretion by insulin-producing β cells, glucagon-producing α cells, and somatostatin-producing δ cells. In type 1 diabetes (T1D), autoimmune destruction of β cells results in loss of glycemic control. Transplantation of cadaveric islets can restore insulin independence in patients with T1D (Shapiro et al., 2000; Brennan et al., 2016), but broader application is limited by donor scarcity and the requirement for chronic immunosuppression or immune-protective devices. Human pluripotent stem cells (hPSCs) can undergo unlimited self-renewal and can be engineered to reduce immune rejection (Sintov et al., 2022; Gerace et al., 2023; Hu et al., 2023), making them an attractive alternative cell source for transplantation therapies (Ramzy et al., 2021; Wang et al., 2024a; Reichman et al., 2025).

**Figure 1:**
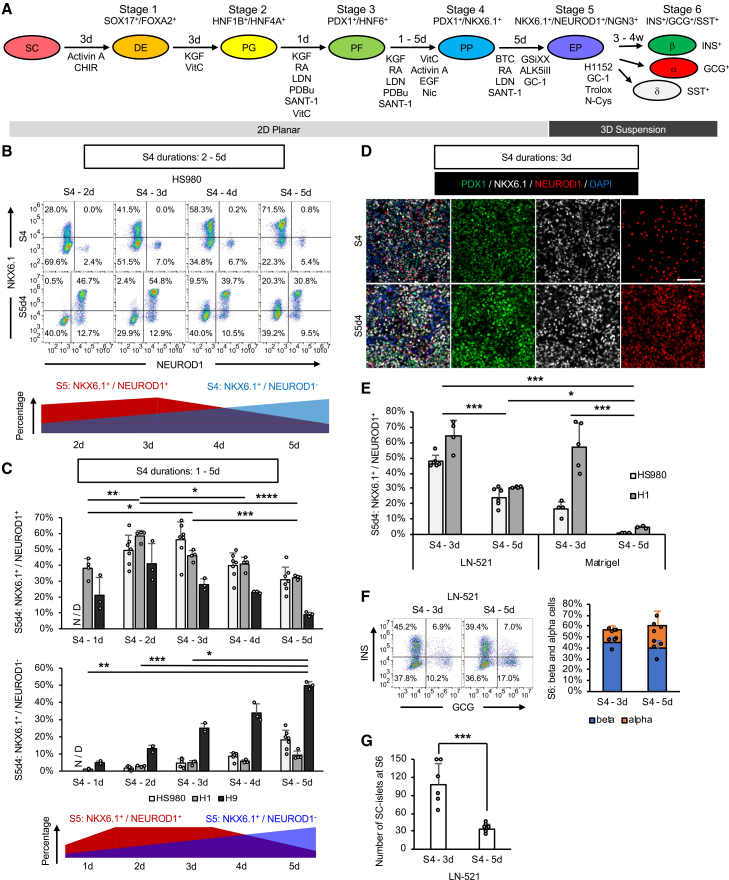
Short S4 duration enhances S5 EP differentiation (A) Schematic of stages S1–S6 with durations, factors, and key markers. (B) NKX6.1 and NEUROD1 expression in HS980 cells on LN-521 with S4 durations of 2–5 days, measured at the end of S4 or S5d4; representative dot plots shown. (C) Percentages of NKX6.1^+^/NEUROD1^+^ and NKX6.1^+^/NEUROD1^−^ cells at S5d4 in HS980, H1, and H9 cells with S4 durations of 1–5 days. Data are means ± SD, *n* = 7 for HS980, 4 for H1, and 3 for H9; one-way ANOVA. (D) Immunofluorescence of PDX1, NKX6.1, and NEUROD1 at S4 and S5d4; representative images, *n* = 3; scale bars, 100 μm. (E) S5 EP differentiation with S4 durations of 3 or 5 days on LN-521 and Matrigel. Bar graphs show NKX6.1^+^/NEUROD1^+^ percentages in HS980 and H1 cells. Data are means ± SD, *n* = 3–6; one-way ANOVA. (F and G) Differentiation to S6 SC-islets from dissociated S5 EP cells with 3- or 5-day S4 on LN-521. (F) INS and GCG expression at S6; representative dot plots and bar graphs show INS^+^/GCG^−^ β and GCG^+^/INS^−^ α cells. Data are means ± SD, *n* = 4. (G) SC-islet counts from 1 × 10^6^ EP cells; data are means ± SD, *n* = 6; unpaired two-tailed *t* test. Statistical significance: ^∗^*p* < 0.05, ^∗∗^*p* < 0.01, ^∗∗∗^*p* < 0.001, ^∗∗∗∗^*p* < 0.0001. See also Figure S1.

Differentiation protocols have advanced substantially and guide hPSCs through stages mimicking pancreatic development (Figure 1A), ultimately producing stem cell-derived pancreatic islets (SC-islets) (D'Amour et al., 2006; Kroon et al., 2008; Pagliuca et al., 2014; Rezania et al., 2014; Nostro et al., 2015; Velazco-Cruz et al., 2019; Veres et al., 2019; Hogrebe et al., 2020; Hogrebe et al., 2021; Balboa et al., 2022; Barsby et al., 2022). However, several key challenges persist. Differentiation beyond the stage (S) 4 pancreatic progenitor (PP) stage frequently yields heterogeneous cultures containing proliferative non-endocrine cells and immature endocrine cells (Sharon et al., 2019b; Veres et al., 2019; Rajaei et al., 2025), increasing the risk of cyst or tumor formation (Kroon et al., 2008; Kelly et al., 2011; Rezania et al., 2012; Aghazadeh et al., 2022; Lithovius et al., 2024). Although efficient induction of PP cells has been achieved in multiple hPSC lines (Nostro et al., 2015; Cogger et al., 2017; Aghazadeh et al., 2022; Balboa et al., 2022), further optimization is needed for the S5 endocrine progenitor (EP) stage and beyond. In addition, SC-islets commonly display reduced glucose-stimulated insulin secretion (GSIS) compared with human islets (Pagliuca et al., 2014; Rezania et al., 2014; Lyon et al., 2016; Cogger et al., 2017; Velazco-Cruz et al., 2019; Davis et al., 2020). Studies of pancreatic development indicate that EP cells delaminate from the epithelium and form 3D aggregates *in vivo* (Jeon et al., 2009; Gouzi et al., 2011; Nair and Hebrok, 2015; Sharon et al., 2019a), but most protocols do not fully recapitulate this process. Moreover, differentiation efficiencies vary across hPSC lines (Rezania et al., 2014).

Here, we report a refined differentiation strategy that consistently generates functional SC-islets across eight hPSC lines. Optimizing EP differentiation on 2D laminin (LN)-521, followed by spontaneous 3D aggregation, produces endocrine clusters devoid of non-endocrine cells that exhibit strong glucose responsiveness *in vitro* and restore glycemic control after transplantation into diabetic mice.

## Results

### Short S4 duration enhances differentiation into S5 pancreatic EP cells

Current differentiation protocols efficiently generate PP cells, but the transition to EP remains suboptimal and often results in heterogeneous, proliferative cultures (Sharon et al., 2019b; Veres et al., 2019). To refine this step, we differentiated hPSCs toward S5 EP stage on 2D LN-521, a defined xeno-free substrate supporting hPSC derivation and expansion (Rodin et al., 2014; Main et al., 2020). The efficiency of differentiation into PDX1^+^/NKX6.1^+^ PP cells on LN-521 is comparable to that on Matrigel (Figures S1A and S1B). Because shortening S3 enhances PP induction at S4 (Nostro et al., 2015; Velazco-Cruz et al., 2019), we reduced S3 to 1 day and evaluated whether the duration of S4 affects downstream EP formation. Reducing S4 duration increased NKX6.1^+^/NEUROD1^+^ EP cells at day 4 of S5 (S5d4), despite lower NKX6.1 expression at S4 (Figure 1B), an effect consistent in two additional cell lines (Figure S1C). Systematic testing identified 2–3 days as optimal, whereas longer S4 generated more NKX6.1^+^/NEUROD1^−^ PP-like cells (Figure 1C). Immunocytochemistry (ICC) confirmed the efficient induction of PDX1^+^/NKX6.1^+^/NEUROD1^+^ and PDX1^+^/NKX6.1^+^/NGN3^+^ EP cells (Figures 1D and S1D). Both short S4 and LN-521 enhanced S5 EP differentiation efficiency, likely through additive effects (Figure 1E). EP cells from short (3-day) or long (5-day) S4 durations on LN-521 were aggregated into 3D at S5d4, generating SC-islets containing both mono-hormonal INS^+^/GCG^−^ β cells and GCG^+^/INS^−^ α cells at the end of S6 (Figure 1F). Short S4 cultures, however, yielded significantly more SC-islets (Figure 1G), indicating that efficient S5 EP differentiation under short S4 conditions is critical for SC-islet formation. Although short S4 enabled S5 EP differentiation on both LN-521 and Matrigel (Figure 1E), LN-521 yielded higher proportions of INS^+^/GCG^−^ β cells at S6 than Matrigel (Figure S1E). As LN-521, also available in GMP-compatible quality, supports hPSC derivation and expansion (Rodin et al., 2014; Main et al., 2020), all subsequent differentiation experiments were performed on LN-521 using 1 day of S3 and 3 days of S4 (hereafter referred to as the short differentiation protocol; see “methods”).

### 3D aggregation at the S5 EP stage enriches endocrine cells and eliminates non-endocrine populations

To identify the optimal stage for transitioning to 3D culture, differentiating cells on LN-521 were dissociated at either the end of S4 or at S5d4, and allowed to aggregate spontaneously (Figure S2A).

**Figure 2:**
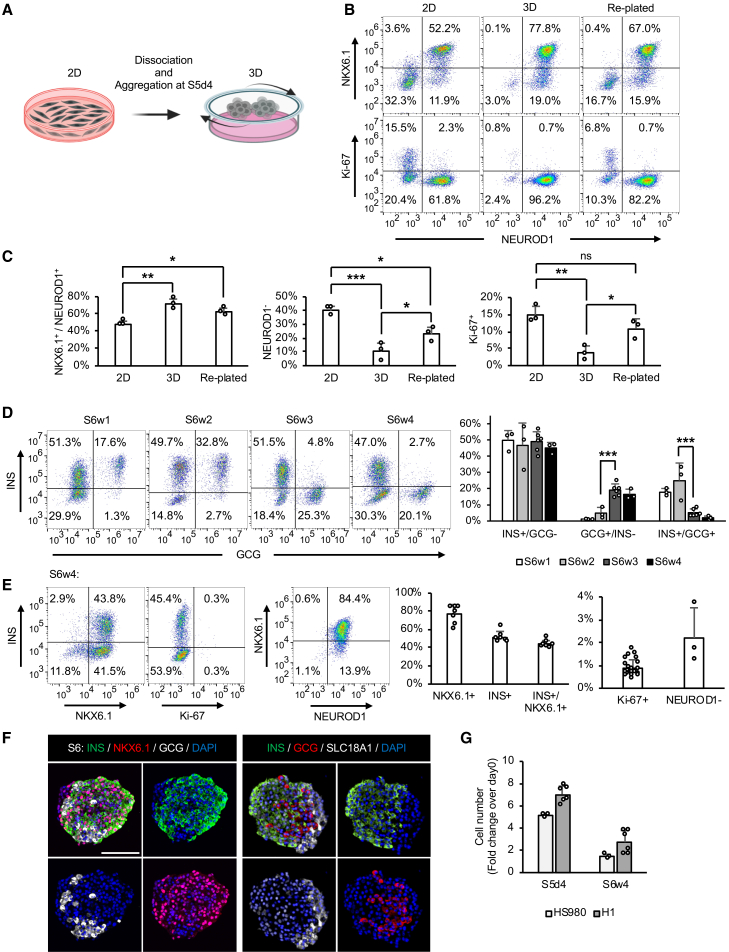
3D aggregate formation at S5 EP stage effectively removes non-endocrine cells (A) Schematic of 3D aggregate formation at S5d4. Cells differentiated on LN-521 were dissociated and maintained in suspension (3D) or re-plated on LN-521. (B and C) NKX6.1, NEUROD1, and Ki-67 expression before (2D) and one day after dissociation (3D, Re-plated) by flow cytometry. (B) Representative dot plots; (C) bar graphs show NKX6.1^+^/NEUROD1^+^ EP cells, NEUROD1^−^ non-endocrine cells, and Ki-67^+^ proliferative cells; data are means ± SD, *n* = 3; one-way ANOVA. (D and E) S6 differentiation from S5d4 in 3D suspension. (D) INS and GCG expression during S6w1–4; representative dot plots and bar graphs; data are means ± SD, *n* = 3–6; one-way ANOVA. (E) NKX6.1, INS, NEUROD1, and Ki-67 expression at S6w4; representative plots and bar graphs; data are means ± SD, *n* = 7 (INS, NKX6.1), 21 (Ki-67), 3 (NEUROD1). (F) Immunofluorescence of INS, GCG, NKX6.1 (left) and SLC18A1 (right) at S6w4; representative images, *n* = 3; scale bars, 100 μm. (G) Cell numbers at S5d4 and S6w4 as fold change over day 0. Data are means ± SD, *n* = 3–6. Statistical significance: ns, not significant; ^∗^*p* < 0.05, ^∗∗^*p* < 0.01, ^∗∗∗^*p* < 0.001. See also Figure S2.

Aggregation of S5 EP cells generated >10-fold more SC-islets at S6, and these aggregates contained higher proportions of INS^+^/GCG^−^ β cells and GCG^+^/INS^−^ α cells compared with aggregates derived from S4 PP cells (Figures S2B and S2C). We next examined whether S5 EP cells were selectively enriched by spontaneous aggregation at S5d4 (Figure 2A). One day after aggregation, EP clusters showed increased NKX6.1^+^/NEUROD1^+^ EP cells and a marked reduction in NEUROD1^−^ non-endocrine and Ki-67^+^ proliferative cells (Figures 2B and 2C). Re-plating dissociated EP cells on LN-521 retained a substantial fraction of these unwanted cells (Figures 2B and 2C), demonstrating that 3D aggregation is required for efficient enrichment of EP cells. ROCK inhibitor (H1152) improved aggregation efficiency and cell recovery (Figure S2E), without affecting EP identity (Figure S2D). Aggregation in either ULA or AggreWell formats yielded similar EP and final endocrine compositions (Figure S2F).

During S6 differentiation, INS^+^/GCG^−^ β cells remained ∼50% of the culture while GCG^+^/INS^−^ α cells progressively increased and INS^+^/GCG^+^ polyhormonal cells declined to <5% (Figure 2D). By week 4 of S6 (S6w4), most β cells co-expressed NKX6.1, and NEUROD1^−^ and Ki-67^+^ cells were further reduced to 2.2 ± 1.32% and 0.88 ± 0.37%, respectively (Figure 2E). ICC confirmed mono-hormonal β and α cells, as well as SLC18A1^+^ EC-like cells (Figure 2F). S5 EP cells could be cryopreserved and later differentiated into SC-islets with comparable endocrine composition and static GSIS (Figures S2G and S2H). Despite ∼40%–50% cell loss during aggregation (Figure S2E), the protocol still produced a net expansion of endocrine cells by S6w4 (Figure 2G; Table S1). Taken together, these results show that S5 EP cells undergo efficient self-aggregation and differentiation into SC-islets, while proliferative and non-endocrine cells are selectively removed.

### Functional SC-islets are generated across multiple hPSC lines

**Figure 3:**
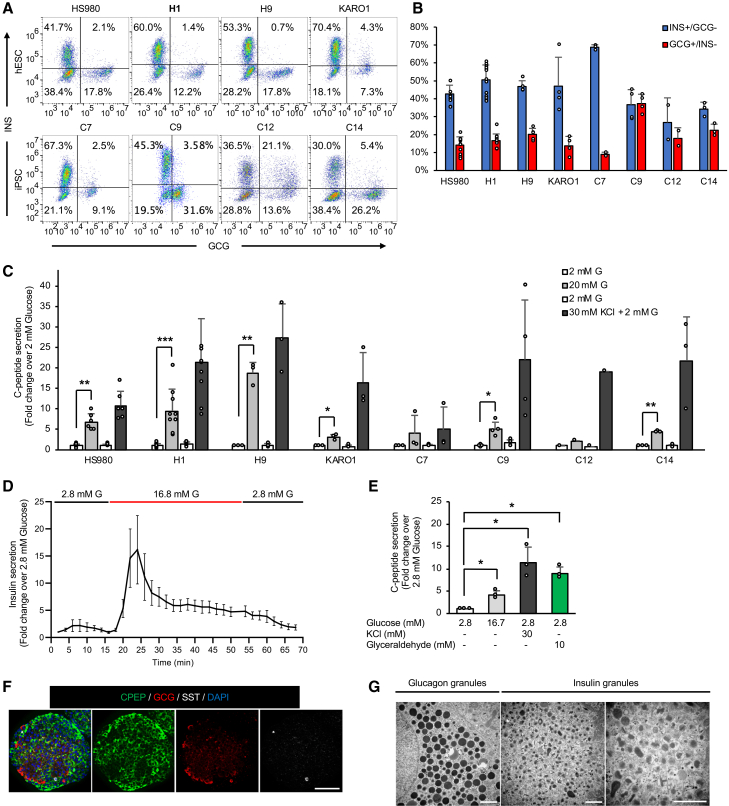
Functional SC-islets generated from multiple hPSC lines (A and B) Eight hPSC lines differentiated using the short differentiation protocol (Wu et al.). INS and GCG assessed by flow cytometry at S6. (A) Representative dot plots and (B) bar graphs are shown. Data are means ± SD, *n* = 12 (HS980), 14 (H1), 5 (H9), 4 (KARO1, C9), 3 (C7, C14), 2 (C12). (C) Static GSIS showing fold change in c-peptide over 2 mM glucose; data are means ± SD, *n* = 6 (HS980), 9 (H1), 3 (H9, KARO1, C7, C14), 4 (C9), 1 (C12); paired two-tailed *t* tests. (D) Dynamic GSIS from HS980 SC-islets; fold change over 2.8 mM glucose; data are means ± SEM, *n* = 4. (E) Glyceraldehyde-stimulated insulin secretion; fold change over 2.8 mM glucose; data are means ± SD, *n* = 3; paired two-tailed *t* test. (F) Immunofluorescence of CPEP, GCG, and SST in H1 SC-islets; representative images, *n* = 3; scale bars, 100 μm. (G) TEM showing insulin and glucagon granules in H1 SC-islets; scale bars, 1 μm. Statistical significance: ^∗^*p* < 0.05, ^∗∗^*p* < 0.01, ^∗∗∗^*p* < 0.001, ^∗∗∗∗^*p* < 0.0001. See also Figure S3.

To address cell-line variability in SC-islet differentiation, we evaluated the protocol across eight hPSC lines, four embryonic (HS980, H1, H9, and KARO1) and four induced pluripotent (C7, C9, C12, and C14), without excluding any line (Figure 3A). All lines generated SC-islets containing appropriate proportions of mono-hormonal INS^+^/GCG^−^ β cells and GCG^+^/INS^−^ α cells (Figure 3B). At the end of S6, SC-islets from all lines showed glucose-stimulated C-peptide secretion with a return to baseline after glucose withdrawal (Figure 3C), within the range reported for freshly isolated human islets (1.4- to 37.3-fold) (Lyon et al., 2016). SC-islets derived from HS980, H1, and H9 exhibited particularly strong glucose responses. Dynamic GSIS of HS980 SC-islets demonstrated a pronounced first-phase insulin release (16.1-fold), followed by sustained second-phase secretion (6.1-fold) (Figure 3D), closely matching human islet responses (15.0- and 6.7-fold) (Velazco-Cruz et al., 2019). To determine whether our protocol overcomes a previously described glycolytic bottleneck (Davis et al., 2020), c-peptide secretion was measured in response to glyceraldehyde. SC-islets responded strongly (Figure 3E), indicating restored glyceraldehyde metabolism, unlike prior SC-islet protocols (Davis et al., 2020; Balboa et al., 2022).

We further characterized SC-islets with ICC and transmission electron microscopy (TEM) (Figures 3F and 3G). Mono-hormonal CPEP^+^ β, GCG^+^ α, and SST^+^ δ cells were confirmed (Figure 3F). β cells contained granules with dark and dense insulin crystalline cores surrounded by a light halo, while α cells displayed large dark vesicles, some with gray halo surrounding a dense core (Figure 3G), both resembling primary human islet ultrastructure (Pagliuca et al., 2014; Peterson et al., 2020; Balboa et al., 2022).

We compared our shortened LN-521-based approach with two published protocols (Velazco-Cruz et al., 2019; Balboa et al., 2022). The 3D long protocol of Velazco-Cruz et al. (5-day S4) (Velazco-Cruz et al., 2019) generated S5 NKX6.1^+^/NEUROD1^+^ EP cells primarily from H1 cells (Figure S3B), whereas our protocol produced EP cells across all eight hPSC lines (Figure S3A), consistent with the combined requirement for both short S4 duration and LN-521 (Figure 1E). Further differentiation of H1 cells using the Velazco-Cruz protocol yielded INS^+^/GCG^−^ β cells, few GCG^+^/INS^−^ α cells, abundant INS^+^/GCG^+^ polyhormonal cells, and minimal static GSIS (1.3-fold; Figures S3C and S3D). The Balboa et al. protocol (Balboa et al., 2022), which maintains cells on 2D through S4 before aggregation, generated more α cells but fewer β cells and exhibited glucose responsiveness (2.75-fold; Figures S3C and S3D). In contrast, our protocol consistently generated mature SC-islets with higher fraction of INS^+^/GCG^−^ β cells and robust glucose-stimulated secretion (9.5-fold).

Collectively, these results demonstrate that the combination of shortened S4, LN-521-based 2D culture, and spontaneous S5 aggregation enables robust and scalable generation of functional SC-islets across diverse hPSC lines, as further supported by comparison to previous protocols (Table S1) (Velazco-Cruz et al., 2019; Augsornworawat et al., 2020; Hogrebe et al. 2020, 2021; Balboa et al., 2022; Barsby et al., 2022; Maxwell et al., 2022; Lithovius et al., 2024; Rajaei et al., 2025).

### Single-cell transcriptome profiling of SC-islets identifies key endocrine but not non-endocrine cell types

**Figure 4:**
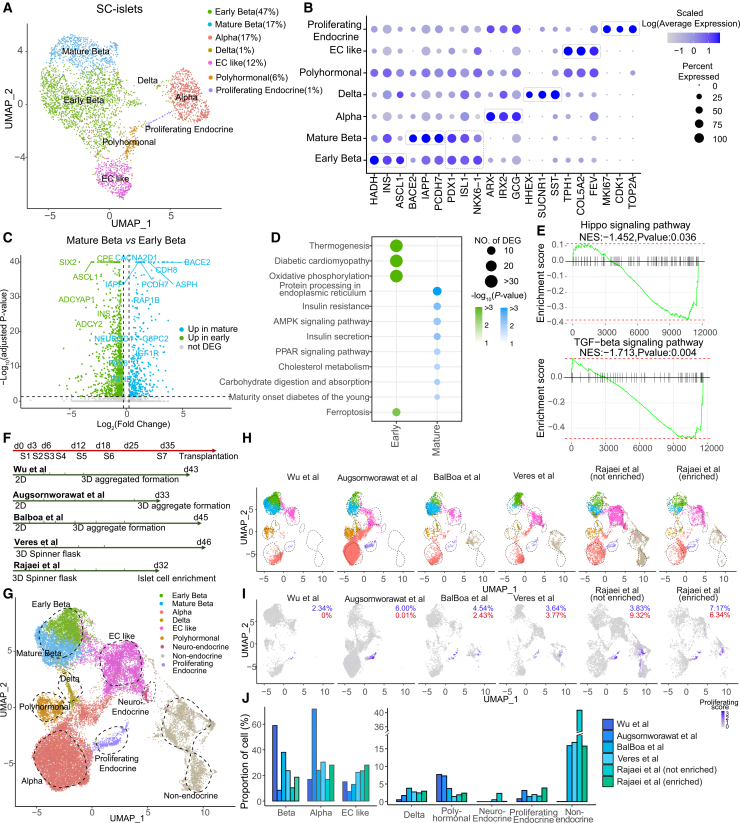
Single-cell transcriptomics define endocrine cell types in SC-islets (A) UMAP of day 43 SC-islets (H1) highlighting major endocrine populations. (B) Dot plot of marker genes used for cell-type annotation. (C) Volcano plot showing fold-change in expression and Bonferroni-adjusted two-sided *p* values for DEGs between mature and early β cells in day 43 H1 SC-islets. (D) KEGG enrichment dot plot for DEGs, showing terms with one-sided *p* < 0.05. Dot size reflects DEG counts in each term and color indicates *p* value. (E) GSEA enrichment curves comparing mature and early β cells. (F) Schematic comparison of timing and 2D/3D formats in published protocols (Augsornworawat, Balboa, Veres, Rajaei) versus Wu et al. (G) Integrated UMAP embedding of datasets from different protocols showing β, α, δ, EC-like, polyhormonal, neuro-endocrine, non-endocrine, and proliferating endocrine clusters. (H) UMAP embedding segregated by protocol. (I) UMAP of the proliferation signature across protocols, showing the percentages of endocrine (blue) and non-endocrine (red) cells with positive proliferation module scores. (J) Cell-type composition across protocols, with major endocrine subsets (β, α, EC-like) displayed on the right and other populations on the left side. See also Figure S4.

Single-cell RNA sequencing (scRNA-seq) of H1 SC-islets at day 43 revealed three major endocrine populations, β, α, and EC-like cells, all expressing chromogranin A (*CHGA*) (Figures 4A, S4A, and S4B). β cells accounted for 64% of all cells and expressed *INS*, *PDX1*, *NKX6.1*, and *ISL1* (Figures 4B, S4B, and S4C). Two β cell sub-clusters were detected: an early population marked by *HADH* (Balboa et al., 2022) and *ASCL1* (Veres et al., 2019), and a mature population expressing *IAPP*, *BACE2* (Díaz-Catalán et al., 2021), *PCDH7* (Yoon et al., 2022), and *CACNA2D1* (Tuluc et al., 2021), key regulators of β cell functional maturation and insulin secretion. Both populations showed high *INS* and low *GCG* expression. α cells (17%) expressed *GCG*, *ARX*, and *IRX2* and, unlike β cells, showed high *GCG* and low *INS* expression (Figures 4A, 4B, S4B, and S4C), consistent with their expected hormone identity.

Differential expression analysis revealed enrichment of *NEUROD1* and *PAX6* in mature β cells, both master regulators of β cell identity and insulin secretion (D'Amour et al., 2006; Gosmain et al., 2012; Mastracci et al., 2013; Bohuslavova et al., 2021), alongside maturation-associated genes such as *BACE2*, *PCDH7*, and *CACNA2D1* (Figure 4C). Pathway analysis highlighted KEGG pathways linked to β cell maturation, including protein processing in the ER, AMPK signaling, insulin secretion, and PPAR signaling (Figure 4D), all central to β cell metabolic and secretory function (Svendsen et al., 2018; Zhu et al., 2019; Entezari et al., 2022). GSEA further showed downregulation of Hippo and TGF-β signaling in mature β cells (Figure 4E), consistent with their roles for β cell differentiation (Mamidi et al., 2018; Rosado-Olivieri et al., 2019; Velazco-Cruz et al., 2019). Together, these data indicate a transition toward enhanced insulin secretory capacity during β cell maturation.

EC-like cells comprised 12% of the SC-islets and expressed *TPH1*, *FEV*, and *COL5A2* (Figures 4A and 4B). Recent studies show that EC-like and SC-β cells display gradient identities (Augsornworawat et al., 2023), and resemble transient 5-HT-producing pre-β cells in fetal pancreas development (Zhu et al., 2023). Three minor populations were detected, namely polyhormonal cells (6%), co-expressing *INS* and *GCG*, δ cells (1%), expressing *SST*, *HHEX*, and *SUCNR1*, and proliferating endocrine cells (1%), expressing proliferation markers *MKI67*, *TOP2A*, and *CDK1* (Figures 4A, 4B, S4B, and S4C). γ and ε cells were rare, showing only sparse marker expression (Figure S4B). Non-endocrine cells were not detected, aside from a few cells co-expressing *CPA1* and *CHGA* but not *TOP2A*, and no mesenchymal, endothelial, or neuronal cells were observed (Figure S4B).

We next compared our SC-islets (Wu et al.) with four published scRNA-seq datasets (Figure 4F). Augsornworawat et al. differentiated HUES8 cells on 2D until stage 6 before 3D aggregation and performed scRNA-seq on day 33 (Augsornworawat et al., 2020). Balboa et al. differentiated H1 cells to S4 PP on 2D, then formed 3D aggregates in microwells and profiled cells on day 45 (S7w3) (Balboa et al., 2022). Veres et al. differentiated HUES8 cells in 3D and analyzed cells on day 46 (S6w4) (Veres et al., 2019). Rajaei et al. reported a GMP-compliant 3D suspension protocol with density-based purification (Rajaei et al., 2025). Integration of all datasets, using the original annotations, revealed distinct cell-type composition across protocols (Figures 4G, 4H, and S4D). Our dataset showed the highest fraction of β cells and the fewest δ cells (Figures 4H, 4J, and S4D). Augsornworawat et al. had the highest α-cell fraction (71%), while all datasets displayed similar levels of EC-like cells (8%–25%) (Figure 4J). Non-endocrine cells were present in Balboa, Veres, and Rajaei et al. datasets (16%–41%) (Figures 4H, 4J, and S4D). A subset of these non-endocrine cells expressed proliferation markers (2.4%–9.3%) (Figure 4I, red), as described by Veres et al., (2019). Previous reports linked such cells to cyst and tumor risk (Kroon et al., 2008; Kelly et al., 2011; Rezania et al., 2012; Aghazadeh et al., 2022; Lithovius et al., 2024). Although all datasets contained endocrine cells with proliferative signatures (*MKI67*, *CDK1*, *TOP2A*, *CCNB2*, *CCNA2*, and *PBK*) (Figure 4I, blue), our SC-islets showed the lowest level (Figure 4I, blue).

### Intraocular transplantation of SC-islets reverses preexisting diabetes in mice

**Figure 5:**
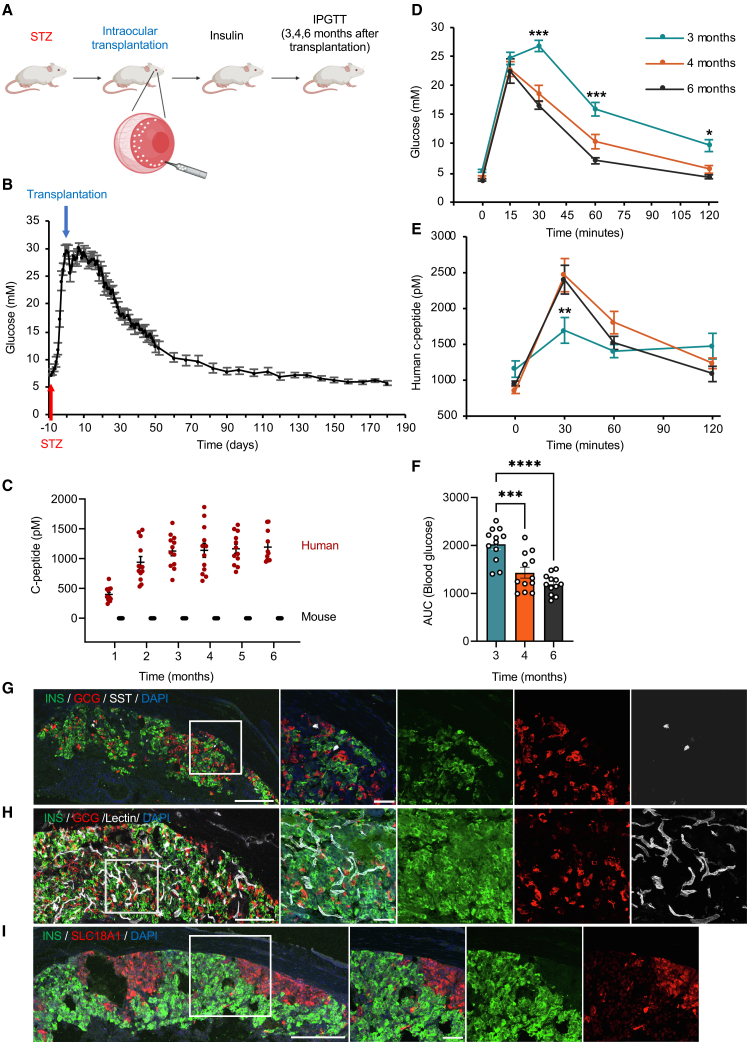
Intraocular SC-islet transplantation reverses preexisting diabetes in mice (A) Experimental design of transplantation and analyses. (B) Non-fasting blood glucose levels (mM) before and after transplantation. (C) Human and mouse plasma c-peptide levels (pM) at indicated time points after transplantation. Data are means ± SEM, *n* = 11–12 (human), 5–9 (mouse). (D–F) IPGTT at 3, 4, and 6 months post-transplantation. (D) Blood glucose and (E) human plasma c-peptide were measured at different time points following glucose injection, at 3, 4, and 6 months post-transplantation. Data are means ± SEM, *n* = 11–12; two-way ANOVA. (F) Quantification of glucose values as area under the curve (AUC). Data are means ± SEM, *n* = 11–12; one-way ANOVA. (G–I) Representative immunohistochemistry of eye sections with SC-islet grafts at 6 months (G) INS, GCG, SST, (H) vessels visualized with lectin, and (I) INS and SLC18A1; representative images, *n* = 3; scale bars: long, 200 μm; short, 50 μm. Statistical significance: ^∗^*p* < 0.05, ^∗∗^*p* < 0.01, ^∗∗∗^*p* < 0.001, ^∗∗∗∗^*p* < 0.0001.

The anterior chamber of the eye (ACE) provides a transparent and accessible site for noninvasive monitoring of engrafted SC-islets through the cornea, while transplantation into this compartment is straightforward and minimally invasive (Speier et al. 2008a, 2008b; Berggren et al., 2024). To evaluate *in vivo* function and maturation, we transplanted SC-islets into the ACE of streptozotocin (STZ)-induced diabetic mice (Figure 5A). Non-fasting blood glucose and plasma human c-peptide levels in 11–12 diabetic mice were monitored for 6 months (Figures 5B and 5C). SC-islet transplantation reversed hyperglycemia by 3 months, and by 5–6 months blood glucose levels fell slightly below pre-STZ baselines (Figure 5B), consistent with species-specific glycemic set points reported previously (Rodriguez-Diaz et al., 2018). Plasma human c-peptide was detectable at 1 month, increased by 3 months, and remained stable, correlating inversely with glucose levels; no mouse c-peptide was detected, confirming glycemic control by SC-β cells (Figure 5C).

Intraperitoneal glucose tolerance tests (IPGTT) at 3, 4, and 6 months post-transplantation showed improved glucose handling over time (Figures 5D–5F). At 3 months, glucose peaked at 30 min and declined by 60 min, whereas at 4 and 6 months it began falling by 30 min and nearly returned to baseline by 120 min (Figure 5D). Plasma human C-peptide increased after glucose injection, with larger responses and a clearer peak-and-decline pattern at 4 and 6 months (Figure 5E). Together, these data indicate enhanced GSIS and continued functional maturation of SC-islets *in vivo*.

At 6 months post-transplantation, eyes were enucleated for cryosectioning. Engrafted SC-islets contained mono-hormonal INS^+^ β, GCG^+^ α, and SST^+^ δ cells (Figure 5G). Lectin staining revealed blood vessels within the grafts, confirming vascularization (Figure 5H). SLC18A1^+^ EC-like cells were also detected in discrete regions of the grafts (Figure 5I).

### SC-islets undergo maturation *in vivo*

**Figure 6:**
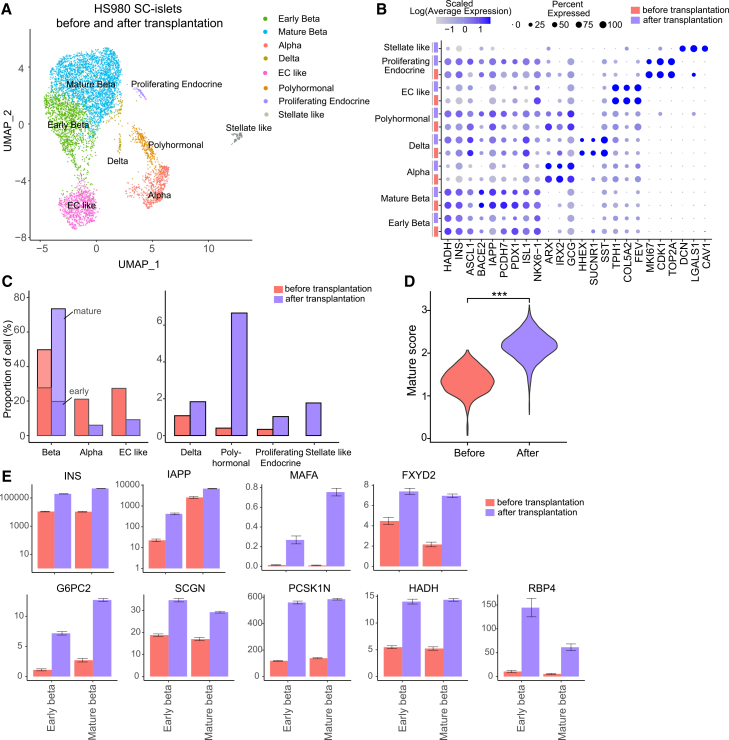
SC-islets undergo maturation *in vivo* (A) UMAP projection of SC-islets from HS980 cells before and 20 weeks after transplantation, showing major cell populations. (B) Dot plot showing the average expression levels of markers used for cell-type annotation; log-transformed values scaled separately for pre- and post-transplantation samples. (C) Cell-type proportions before and after transplantation. (D) Maturation signature of β cells and expression of genes involved in insulin secretion pre- versus post-transplantation; Wilcoxon test, ^∗∗∗^*p* < 0.001. (E) Expression of selected DEGs in early and mature beta cells across pre- and post-transplantation states. See also Figures S5 and S6.

To assess *in vivo* maturation, HS980 SC-islets were analyzed by scRNA-seq 20 weeks after transplantation. The same endocrine populations seen pre-transplantation were identified; α, early β, mature β, δ, EC-like, polyhormonal, and proliferating endocrine cells, all expressing expected markers (Figures 6A–6C and S5A–S5H). Mature β cells increased from 22% to 54%, while early β cells decreased from 28% to 20%, yielding a total β-cell mass of 74% (Figure 6C). α and EC-like cells declined, whereas polyhormonal cells expanded (Figure 6C). A minor population of non-proliferating pancreatic stellate cells expressing the ECM gene *DCN*, but not *TOP2A*, emerged post-transplantation (Figures 6A–6C and S5H), indicating a low risk of islet fibrosis or related damage (Wang et al., 2024b). Proliferating β cells (1%) co-expressing *INS* and *TOP2A* were also detected (Figures 6A–6C, S5G, and S5H), consistent with the low β cell proliferation rate reported previously (Kassem et al., 2000). Few cells expressed *CPA1*, but all co-expressed *CHGA*, indicating the absence of true exocrine or other non-endocrine cells (Figures S5F and S5H). No EP, mesenchymal, endothelial, or neuronal cells were observed (Figures S5F and S5H).

RNA velocity revealed trajectories from early to mature β cells and from α to polyhormonal cells (Figure S6A), consistent with the increased mature β and polyhormonal fractions after transplantation (Figure 6C). To examine *in vivo* β cell maturation (Figure 6D), we compared gene expression in early and mature β cells before and after transplantation (Figures 6E and S6B–S6D). Most differentially expressed genes were upregulated within the same β cell subpopulation post-transplantation (Figure S6C), including genes associated with mature β cell identity and insulin secretion (Figures 6E and S5E–S5H), indicating *in vivo* maturation consistent with previous studies (Augsornworawat et al., 2020; Balboa et al., 2022). Hippo and TGF-β signaling gene sets were downregulated in mature β cells before and after transplantation (Figures S6B and S6D), supporting their role in functional β cell differentiation (Mamidi et al., 2018; Rosado-Olivieri et al., 2019; Velazco-Cruz et al., 2019).

## Discussion

Cell therapy for T1D requires efficient *in vitro* generation of transplantable SC-islets. Previous protocols exhibit variable efficiency across hPSC lines and often produce SC-islets with immature function and non-endocrine contaminants. Here, we present a new protocol for generating functional SC-islets from multiple hPSC lines, featuring two key improvements: (1) optimization of the S4 PP duration on 2D LN-521 to enhance S5 EP differentiation and (2) spontaneous 3D aggregation of single S5 EP cells, which removes proliferative and non-EP cells and yields SC-islets with greatly improved endocrine purity.

During stepwise pancreatic differentiation, high efficiency at intermediate stages is essential to prevent heterogeneous final populations. A recent study showed that extended expansion enhances early endodermal progenitor differentiation (Wong et al., 2023). Efficient generation of S4 NKX6.1^+^ PP cells is particularly important, as endocrine induction before NKX6.1 produces polyhormonal rather than functional β cells (Kelly et al., 2011; Sharon et al., 2019b; Veres et al., 2019; Hogrebe et al., 2020; Peterson et al., 2020). Although earlier protocols used a long (5-day) S4 stage to enhance PP formation (Pagliuca et al., 2014; Cogger et al., 2017; Aghazadeh et al., 2022), we found that prolonged S4 duration impairs progression to S5 EP cells (Figure 1B). Shortening S4 to 2–3 days markedly improves S5 EP differentiation (Figure 1C), suggesting that S4 PP cells possess a limited temporal window of endocrine competence. Prolonged S4 culture may therefore reduce the fraction of cells able to initiate the endocrine differentiation program, resulting in fewer S5 EP cells.

To generate functional SC-islets, 2D progenitor cells must be organized into 3D aggregates. Previous methods using multipotent S4 PP cells often required enforced aggregation (Balboa et al., 2022; Barsby et al., 2022), yielding heterogeneous populations with proliferative non-endocrine cells (Figure 4I). In contrast, aggregation at S5 EP or later at S6, as in our protocol and in Augsornworawat et al., (2020), substantially reduced non-endocrine contaminants (Figures 4H–4J). We reasoned that S5 NEUROD1^+^ EP cells could spontaneously form 3D islet-like aggregates, mirroring NeuroD1^+^ cluster formation during mouse pancreas development (Gouzi et al., 2011; Sharon et al., 2019a). Indeed, S5 NEUROD1^+^ EP cells formed 3D aggregates that excluded most NEUROD1^−^ non-endocrine and Ki-67^+^ proliferative cells (Figures 2A–2C). Aggregation was enhanced by low-dose ROCK inhibition (Figure S2E), reflecting the limited survival of non-adherent single EP cells and consistent with EP cells migrating cohesively into NeuroD1^+^ clusters, as described by Sharon et al. (2019a).

Our protocol generated glucose-responsive SC-islets from all eight hPSC lines tested (Figures 3A–3D), demonstrating potential for autologous applications. scRNA-seq confirmed high endocrine purity, with only 1% proliferative endocrine cells and no non-endocrine contaminants, though EC-like cells remained (Figures 2F, 4A, and 4F–4J). Transplantation into diabetic mice reversed hyperglycemia and restored glucose homeostasis (Figure 5). Post-transplant analysis showed *in vivo* maturation of SC-β cells and a reduced fraction of EC-like cells (Figures 6C–6E). No graft growth or cysts were observed after 6 months. While a minor population of pancreatic stellate cells was detected, these cells were largely non-proliferative, suggesting a low risk of islet fibrosis or related damage.

Several limitations should be noted. The molecular basis of the enhanced endocrine differentiation following S4 shortening remains unclear. β cell function was demonstrated by GSIS assays and reversal of hyperglycemia after transplantation. Due to considerable variabilities between primary human islet preparations, a direct comparison was not meaningful and therefore not performed. Further studies including electrophysiology, Ca^2+^ imaging, responses to other secretagogues, and assessment of glucagon secretion from α cells would in addition define endocrine maturity. Finally, although transplantation restored glycemic control without cyst formation for up to 6 months, longer-term studies are needed to assess graft stability and potential stromal remodeling.

Transplantation of autologous iPSC-derived islets has achieved sustained insulin independence (Wang et al., 2024a), and sufficiently pure, mature autologous SC-islets are expected to reduce immune rejection. Our efficient differentiation protocol represents a key step toward autologous cell therapy, though further work is required to realize this goal.

## Methods

### hPSC culture and differentiation

Human ESC lines HS980 (KIe033-A), KARO1 (KIe034-A), H1 (WAe001-A), and H9 (WAe009-A), and iPSC lines C7, C9, C12, and C14, were cultured under xeno-free conditions in NutriStem hPSC XF Medium (Biological Industries, 05-100-1A) on tissue-culture plates coated with 10 μg/mL laminin (LN)-521 (BioLamina, LN521) (Rodin et al., 2014; Main et al., 2020; Plaza Reyes et al., 2020). Cells were maintained at 37°C, 5% CO_2_, and 5% O_2_, routinely confirmed mycoplasma-free, and validated for pluripotency marker expression by flow cytometry. Cells were passaged every 3–5 days using TrypLE Select (Thermo Fisher, A1285901) and re-plated at 15,000–24,000 cells/cm^2^. Full culture details are provided in supplemental methods.

Upon reaching 90%–100% confluence, hPSCs were differentiated into pancreatic islet cells at 37°C, 5% CO_2_, and 20% O_2_, progressing through six stages (S1–S6) using the following factors:

**S1 Definitive endoderm** (3 days): 100 ng/mL Activin A (R&D, 338-AC) and 5 μM CHIR99021 (Tocris, 4423) for the first 24 h, followed by 100 ng/mL Activin A for 2 additional days.
**S2 Primitive gut tube** (3 days): 50 ng/mL KGF (R&D, 251-KG).
**S3 Posterior foregut** (1 day): 50 ng/mL KGF, 2 μM retinoic acid (Sigma, R2625), 0.25 μM SANT-1 (Sigma, S4572), 0.5 μM PDBu (Tocris, 4153), and 200 nM LDN193189 (Tocris, 6053).
**S4 PP** (2–3 days): 50 ng/mL KGF, 100 ng/mL EGF (R&D, 236-EG), 5 ng/mL Activin A, 10 mM nicotinamide (Sigma, N0636), 100 nM retinoic acid, 0.25 μM SANT-1, 0.5 μM PDBu, and 200 nM LDN193189.
**S5 EP** (5 days): 20 ng/mL Betacellulin (R&D, 261-CE), 100 nM retinoic acid, 0.25 μM SANT-1, 100 nM GSI-XX (Sigma, 565789), 10 μM ALK5 inhibitor II (Cayman Chemical, 14794), 1 μM GC-1 (Tocris, 4554), and 100 nM LDN193189.

On day 4, cells were dissociated with Accutase, resuspended at 1.0–1.5 × 10^6^ cells/mL in ultra-low attachment plates (Corning, 3471) with 10 μM ROCK inhibitor H1152 (Tocris, 2414), and cultured on an orbital shaker (Infors HT Celltron) at 95 rpm to form islet-like aggregates.

**S6 Pancreatic islets** (3–4 weeks): 10 μM H1152, 1 μM GC-1, 1 mM N-acetyl-L-cysteine (Sigma, A9165), and 10 μM Trolox (Merck Millipore, 648471). Aggregates maintained on orbital shaker (95 rpm).

Media were changed daily from S1 to S5 and every 2–3 days during S6. Full daily media compositions, factor concentrations, and details of short versus long differentiation protocols are provided in supplemental methods.

### Flow cytometry

Cells were dissociated with Accutase, washed, and resuspended at 1 × 10^6^ cells/mL in PBS. Live/dead staining was performed for 30 min at 4°C (Thermo Fisher, L34963 and L34965). After two PBS washes, cells were fixed in Cytofix/Cytoperm buffer (BD, 554722) for 20 min at 4°C, washed, and incubated with conjugated antibodies in 1× Perm/Wash buffer (BD, 554723) for 30 min at 4°C. Following two additional washes, cells were resuspended in FACS buffer (PBS + 2% FBS + 1 mM EDTA) and analyzed on a Beckman Coulter CytoFLEX S flow cytometer. Data were processed using FlowJo v10.8.1. Antibodies are listed in Table S2.

### Immunofluorescence

Cells were fixed in 4% paraformaldehyde for 20 min at room temperature (RT) and washed in PBS. Samples were blocked in 5% normal donkey serum (Merck Millipore; S30-100 mL) with 0.3% Triton X-100 (Sigma, T9284) for 1 h at RT, then incubated overnight at 4°C with primary antibodies diluted in PBS containing 0.1% Triton X-100 and 5% normal donkey serum. After washing, secondary antibodies were applied for 1 h at RT. For optical clearing, SC-islets were incubated in FocusClear (CelExplorer, FC-101) overnight at 4°C and mounted in MountClear (CelExplorer, MC-301). Imaging was performed using a Nikon ECLIPSE Ti2 spinning-disk confocal microscope.

Mouse eyes containing SC-islets were excised 6 months post-transplant, fixed in 10% formalin overnight at 4°C, washed in PBS, transferred sequentially to 10%, 20%, and 30% sucrose, embedded in optimal cutting temperature compound (OCT, Sakura Finetek, 4583), and frozen at −80°C. For vessel visualization, some mice before sacrifice received tail-vein injection of 100 μL Lycopersicon Esculentum Lectin, DyLight 649 (1 mg/mL, Thermo Fisher, L32472). Cryo-sections (16 μm) were permeabilized with 0.1% Triton X-100 for 15 min, blocked with 10% FBS for 2 h at RT, and stained with primary and secondary antibodies in buffer containing 1% FBS and 0.01% Triton X-100. Sections were mounted in ProLong Gold antifade reagent with DAPI (Thermo Fisher, P36931) and imaged on a Leica TCS SP8 X confocal microscope. Images were processed using Fiji.

Primary antibodies are listed in Table S3; secondary antibodies were conjugated to Alexa Fluor 488, 546, 633, and 647 (Thermo Fisher).

### Static *in vitro* GSIS

SC-islets (20–30 per assay; S6w4) were preconditioned overnight in S6 medium without ITS-X (5 mM glucose). Islets were washed and pre-incubated for 2 h in Krebs buffer containing 2 mM glucose, then sequentially incubated for 30 min in Krebs buffer containing 2 mM glucose, 20 mM glucose, 2 mM glucose, and 2 mM glucose plus 30 mM KCl, with washes between steps. Supernatants were collected for human C-peptide quantification by ELISA (R&D, DICP00) and normalized to total cell number after Accutase dissociation. Detailed buffer composition and handling steps are provided in supplemental methods.

### TEM

SC-islets at the end of S6 were immersion-fixed in 2.5% glutaraldehyde and 1% formaldehyde in 0.1 M phosphate buffer (pH 7.4) for 1 h at RT and stored at 4°C. Samples were rinsed and post-fixed in 2% OsO_4_ in 0.1 M phosphate buffer for 2 h at 4°C, dehydrated through graded ethanol and acetone, and embedded in LX-112 resin (Ladd Research Industries). Ultrathin sections (80–100 nm) were cut using a Leica EM UC7 ultramicrotome, mounted on formvar-stabilized slot grids, and contrasted with uranyl acetate and lead citrate. Imaging was performed on a Hitachi HT7700 TEM at 80 kV with a 2k × 2k Veleta CCD camera (Olympus SIS).

### Dynamic GSIS assay

Dynamic GSIS was measured using a Biorep PERI-4.2 perifusion system with SC-islets. Effluent was collected at regular intervals, and insulin concentrations were quantified using an AlphaLISA kit. Detailed protocols are provided in supplemental methods.

### scRNA-seq

S6 SC-islets from H1 and HS980 cells, and post-transplant grafts, were dissociated into single cells and processed for scRNA-seq using 10× Genomics Chromium Next GEM 3′ kits. Libraries were sequenced on an Illumina NextSeq 2000, and data were aligned to the human reference genome (GRCh38) with mouse reads removed for graft samples. High-quality cells were retained for downstream analysis with Seurat package (v5.1.0) (Hao et al., 2021), with clustering, UMAP visualization, and differential gene expression performed as described in supplemental methods. Integration and batch correction of pre- and post-transplant datasets, as well as comparisons to published differentiation protocols, were conducted using fastMNN and multiBatchNorm from SeuratWrappers package (v0.3.5) and batchelor package (v1.18.1) (Haghverdi et al., 2018), respectively. Proliferation and β-cell maturation scores were computed with AddModuleScore from Seurat package. RNA velocity analysis was performed on HS980 and grafted cells using scvelo. Detailed sample preparation, analysis pipelines, and parameters are provided in supplemental methods.

### Transplantation studies

All animal procedures were approved by Regional Ethical Committee at Karolinska Institutet. Six- to seven-week-old NSG mice (Jackson Laboratories) were maintained under standard conditions. Diabetes was induced at 8 weeks by intraperitoneal injection (i.p.) of STZ (60 mg/kg body weight, 4 consecutive days). Non-fasting blood glucose was monitored throughout, and monthly tail-vein samples were used to measure human and mouse c-peptide by species-specific ELISA.

Diabetic NSG mice were anesthetized with isoflurane, and HS980 SC-islets (280–300 per eye) were transplanted into the anterior chamber via a corneal puncture using a glass microcannula connected to a syringe. Postoperative care included ocular lubrication and subcutaneous analgesia (Temgesic, 0.1 μg/g). Mice were supported with long-acting insulin (Insulatard Penfill, Novo Nordisk) at 0.05–0.15 IU s.c. from days 4–5 post-transplant for 45–56 days. Glucose responsiveness was assessed by IPGTT after 5 h fasting, measuring blood glucose at 0, 15, 30, 60, and 120 min and plasma c-peptide at 0, 30, 60, and 120 min. Detailed procedures are provided in supplemental methods.

### Statistical analysis

Data are shown as mean ± SD or SEM (*n* = independent experiments). Two-tailed paired or unpaired *t* tests were used for two-group comparisons; one-way ANOVA with Tukey’s test or two-way ANOVA with Bonferroni’s test was applied for multiple groups or time points. Significance was set at *p* < 0.05. Analyses were performed in GraphPad Prism and Excel.

## Resource availability

### Lead contact

Requests for further information and resources should be directed to and will be fulfilled by the lead contact, Siqin Wu (siqinw@gmail.com).

### Materials availability

This study did not generate new unique reagents.

### Data and code availability

The accession number for the single-cell RNA sequencing data reported in this paper is Gene Expression Omnibus: GSE270864. Analysis code is available at https://github.com/kblust/SC-islets.

## Acknowledgments

Open access funding was provided by 10.13039/501100004047Karolinska Institutet. This work was supported by 10.13039/501100001858VINNOVA; Spiber Technologies AB; the Swedish Research Council; Swedish Foundation for Strategic Research; 10.13039/100007459Ragnar Söderberg Foundation; Ming Wai Lau Center for Reparative Medicine; Wallenberg Academy Fellow; 10.13039/501100004063Knut and Alice Wallenberg Foundation; 10.13039/501100004973Barndiabetesfonden; National ATMP Research School; Family Erling-Persson Foundation; 10.13039/501100009708Novo Nordisk Foundation; Jonas & Christina af Jochnick Foundation; Swedish Diabetes Association; ERC grant ERC-2018-AdG 834860 EYELETS; and Karolinska Institutet Strategic Research Programs. Imaging was performed at the Live Cell Imaging Unit/Nikon Center of Excellence and BioNut. Flow cytometry was performed at MedH Flow Cytometry core facility, and prenatal human tissue was provided by Developmental Tissue Bank. Sequencing was conducted at ESCG Infrastructure and Bioinformatics units at Science for Life Laboratory. Transmission electron microscopy was performed at the electron microscopy core facility (EMil) at the Department of Laboratory Medicine Karolinska Institute.

## Author contributions

S.W., M.H., P.-O.B., and F.L. conceived the study; S.W., S.C., P.E., and E.E. conducted *in vitro* experiments; G.B. performed *in vivo* studies; C.Z. and K.B. analyzed scRNA-seq data; S.W., G.B., S.C., C.Z., and K.B. assembled and interpreted the data; S.W. wrote the original draft. All authors reviewed, edited, and approved the manuscript.

## Declaration of interests

S.W. is the inventor on patent applications (WO2024033299A1 and WO2024033300A1) related to the pancreatic differentiation protocol described in this paper. S.W. is employed by Spiber Technologies AB. P.E. was employed by Spiber Technologies AB. M.H. holds shares in Spiber Technologies AB. P.-O.B. is CEO of Biocrine AB.

## Supplemental information

Document S1. Figures S1–S6, Tables S1–S3, and Supplemental Methods

Document S2. Article plus supplemental information
