# Supplementary material for: An optimized protocol for efficient derivation of pancreatic islets from multiple human pluripotent stem cell lines
Source: Stem Cell Reports. 2026 Apr 16;21(5):102892. doi: 10.1016/j.stemcr.2026.102892 (PMC13163220; doi:10.1016/j.stemcr.2026.102892)
Supplement: Document S1. Figures S1–S6, Tables S1–S3, and Supplemental Methods [file mmc1.pdf]

Stem Cell Reports, Volume *21*

Supplemental Information

An optimized protocol for efficient derivation of pancreatic islets from multiple human pluripotent stem cell lines

Siqin Wu, Shivam Chandel, Galyna Bryzgalova, Paschalis Efstathopoulos, Kelly Blust, Cheng Zhao, Eda Erbil, Anna Falk, My Hedhammar, Per-Olof Berggren, and Fredrik Lanner

Supplemental Information

An optimized protocol for efficient derivation of pancreatic islets from multiple human pluripotent stem cell lines

Siqin Wu, Shivam Chandel, Galyna Bryzgalova, Paschalis Efstathopoulos, Kelly Blust, Cheng Zhao, Eda Erbil, Anna Falk, My Hedhammar, Per-Olof Berggren, Fredrik Lanner

Figure S1

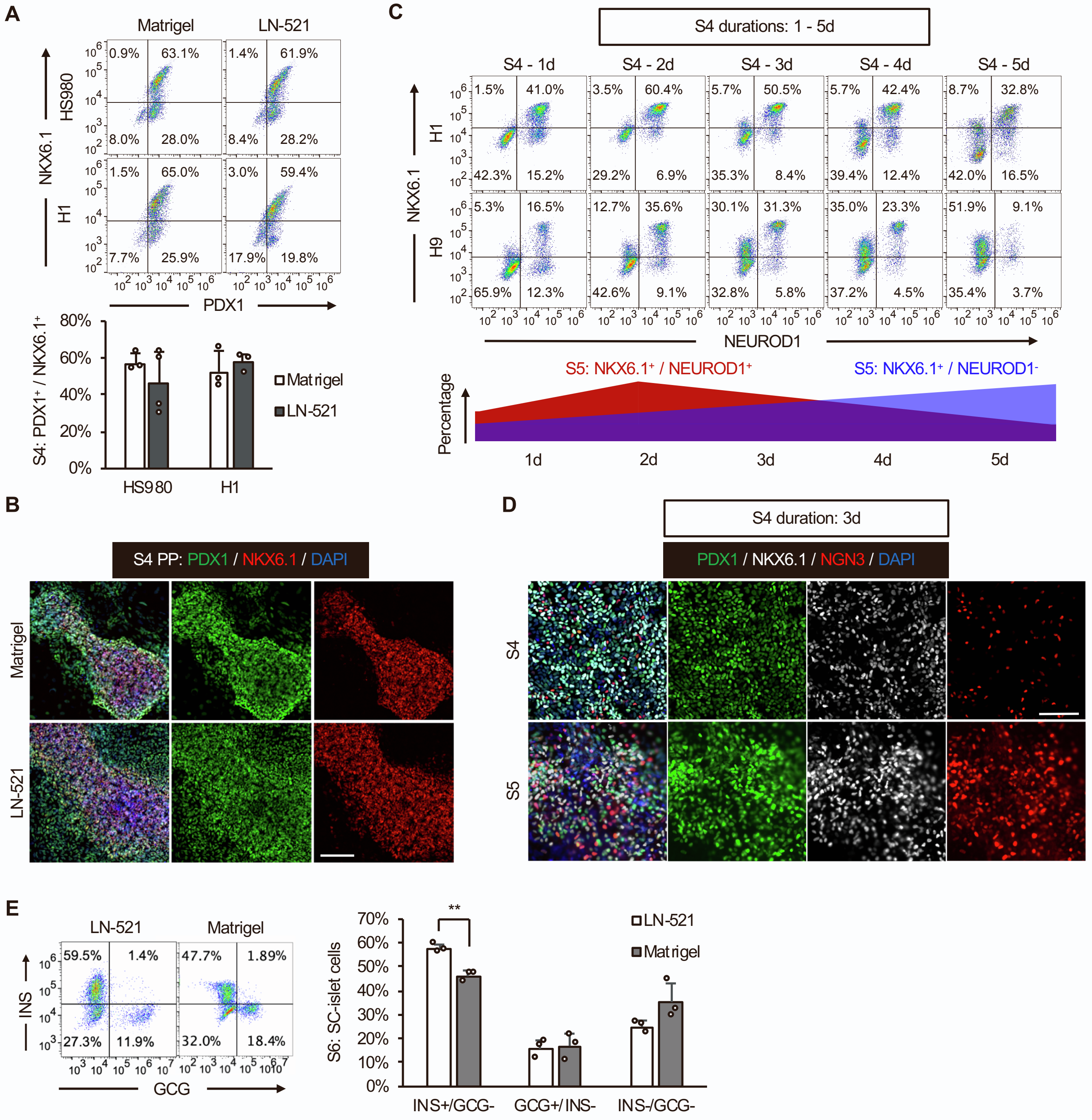
A
Matrigel
LN-521
HS980
H1
NKX6.1
PDX1
0.9%
63.1%
8.0%
28.0%
1.4%
61.9%
8.4%
28.2%
1.5%
65.0%
7.7%
25.9%
3.0%
59.4%
17.9%
19.8%
S4: PDX1+ / NKX6.1+
80%
60%
40%
20%
0%
HS980
H1
Matrigel
LN-521
C
S4 durations: 1 - 5d
S4 - 1d
S4 - 2d
S4 - 3d
S4 - 4d
S4 - 5d
H1
H9
NKX6.1
NEUROD1
1.5%
41.0%
42.3%
15.2%
3.5%
60.4%
29.2%
6.9%
5.7%
50.5%
35.3%
8.4%
5.7%
42.4%
39.4%
12.4%
8.7%
32.8%
42.0%
16.5%
5.3%
16.5%
65.9%
12.3%
12.7%
35.6%
42.6%
9.1%
30.1%
31.3%
32.8%
5.8%
35.0%
23.3%
37.2%
4.5%
51.9%
9.1%
35.4%
3.7%
S5: NKX6.1+ / NEUROD1+
S5: NKX6.1+ / NEUROD1-
Percentage
1d
2d
3d
4d
5d
B
S4 PP: PDX1 / NKX6.1 / DAPI
Matrigel
LN-521
D
S4 duration: 3d
PDX1 / NKX6.1 / NGN3 / DAPI
S4
S5
E
LN-521
Matrigel
INS
GCG
59.5%
1.4%
27.3%
11.9%
47.7%
1.89%
32.0%
18.4%
S6: SC-islet cells
70%
60%
50%
40%
30%
20%
10%
0%
**
LN-521
Matrigel
INS+/GCG-
GCG+/INS-
INS-/GCG-

Figure S1. Pancreatic islet differentiation on recombinant human laminin coatings, related to Figure 1.

(**A** and **B**) Differentiation towards S4 PP on Matrigel and recombinant human laminin (LN) -521 using the long differentiation protocol as described in Methods. The expression of PP markers PDX1 and NKX6.1 were examined at the end of S4 (S4) by flow cytometry and immunocytochemistry (ICC). (**A**) Representative dot plots (upper) and bar graphs (lower) representing the results for both HS980 and H1 cells. Data are means ± SD, n = 3 - 4. (**B**) Fluorescence microscope images showing expression of PDX1 and NKX6.1. n = 3. Scale bar = 100 μM. (**C**) Differentiation towards S5 EP on LN-521 with S4 durations of 1 - 5 days. The expression of EP markers NKX6.1 and NEUROD1 in H1 and H9 cells were examined by flow cytometry at day 4 of S5 (S5d4). Representative dot plots are shown. n = 3 - 4. Results from statistical analysis are shown in Figure 1C. (**D**) Immunofluorescence analysis of expression of EP markers PDX1, NKX6.1, and NGN3 at S4 and S5d4 (S5). The S4 duration is 3 days on LN-521. n = 3. Scale bar = 100 μM. (**E**) Differentiation towards S6 SC-islet using the short differentiation protocol as described in Methods. H1 cells from LN-521 and Matrigel were dissociated into single cells at S5d4 and then aggregated in 3D suspension. Representative dot plots (left) and bar graphs (right) show the expression of INS and GCG at S6w4, as determined by flow cytometry. Data are means ± SD, n = 3. Unpaired 2-tailed t-tests, ** $p < 0.01$.

Figure S2

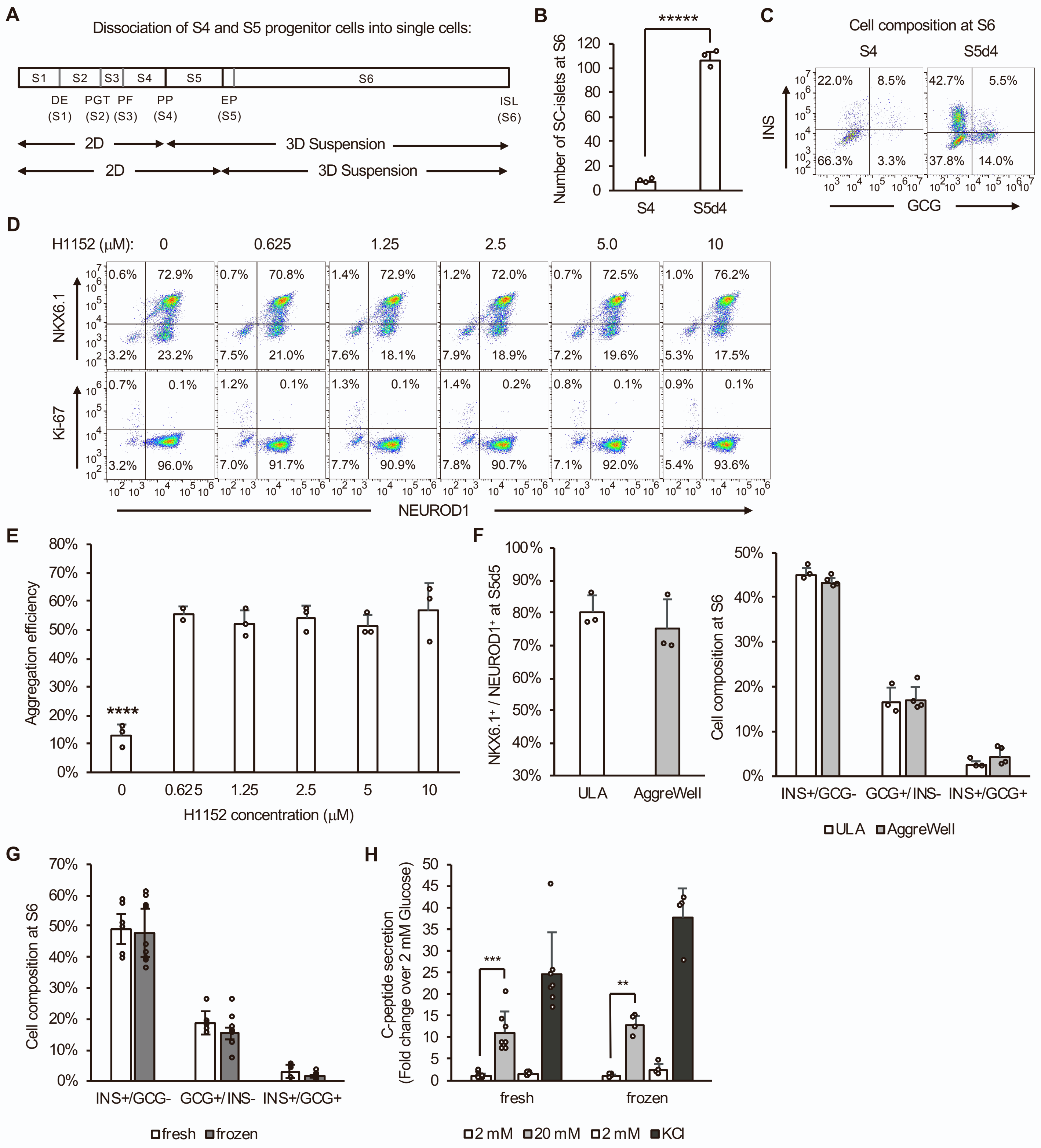
A
Dissociation of S4 and S5 progenitor cells into single cells:
S1 S2 S3 S4 S5 S6
DE (S1) PGT (S2) PF (S3) PP (S4) EP (S5) ISL (S6)
2D
3D Suspension
B
Number of SC-islets at S6
*****
S4
S5d4
C
Cell composition at S6
S4
S5d4
INS
GCG
22.0%
8.5%
66.3%
3.3%
42.7%
5.5%
37.8%
14.0%
D
H1152 (μM): 0 0.625 1.25 2.5 5.0 10
NKX6.1
Ki-67
NEUROD1
E
Aggregation efficiency
****
H1152 concentration (μM)
F
NKX6.1+ / NEUROD1+ at S5d5
ULA
AggreWell
Cell composition at S6
INS+/GCG-
GCG+/INS-
INS+/GCG+
G
Cell composition at S6
fresh
frozen
H
C-peptide secretion (Fold change over 2 mM Glucose)
***
**
2 mM
20 mM
2 mM
KCl

Figure S2. SC-islet formation of S5 EP cells in 3D suspension culture, related to Figure 2.

(**A**) Schematic representation showing the timeline of differentiation on LN-521. The cells were dissociated into single cells at the end of S4 (S4, upper timeline), or day 4 of S5 (S5d4, lower timeline), and then maintained in ULA-treated well (3D suspension) to generate SC-islets. (**B**) Bar graphs representing the numbers of S6 SC-islets generated from $1x10^6$ single S4 and S5d4 cells. Data are means ± SD, *n* = 3. Unpaired 2-tailed t-test, ***** p < 0.00001. (**C**) The expression of islet markers INS and GCG was measured by flow cytometry at the end of S6. Representative dot plots are shown. *n* = 3. (**D** and **E**) Effects of ROCK inhibitor H1152 on 3D aggregate formation at S5d4. Different concentrations of H1152, from 0 to 10 μM, were added to the single cell suspension for 24 hours as indicated. (**D**) The expression of NKX6.1, NEUROD1, and Ki-67 were examined by flow cytometry at S5d5. Representative dot plots are shown. *n* = 3. (**E**) The aggregation efficiencies were examined at S5d5, as described in Methods. Data are means ± SD, *n* = 3. One-way ANOVA, **** p < 0.0001. (**F**) The dissociated S5 cells were maintained in ULA-treated well (ULA) or on AggreWell plate (AggreWell) from S5d4. The expression of NKX6.1 and NEUROD1 at S5d5, and INS and GCG at the end of S6, were measured by flow cytometry. Bar graphs show the percentages of $NKX6.1^+/NEUROD1^+$ EP cells at S5d5 (left), and INS^+/GCG^- β, GCG^+/INS^- α, and INS^+/GCG^+ polyhormonal cells at S6 (right). Data are means ± SD, *n* = 3 for ULA, and 3 - 4 for AggreWell. (**G**) Bar graphs show the percentages of INS^+/GCG^- β, GCG^+/INS^- α, and INS^+/GCG^+ polyhormonal cells in SC-islets derived from fresh and frozen S5d4 cells. Data are means ± SD, *n* = 6 for fresh and 9 for frozen. (**H**) Bar graphs show the results of static *in vitro* GSIS, presented as fold change in c-peptide secretion over 2 mM Glucose set to 1. Data are means ± SD, *n* = 7 for fresh, and 4 for frozen. Paired 2-tailed *t*-tests, ** p < 0.01, *** p < 0.001.

Figure S3

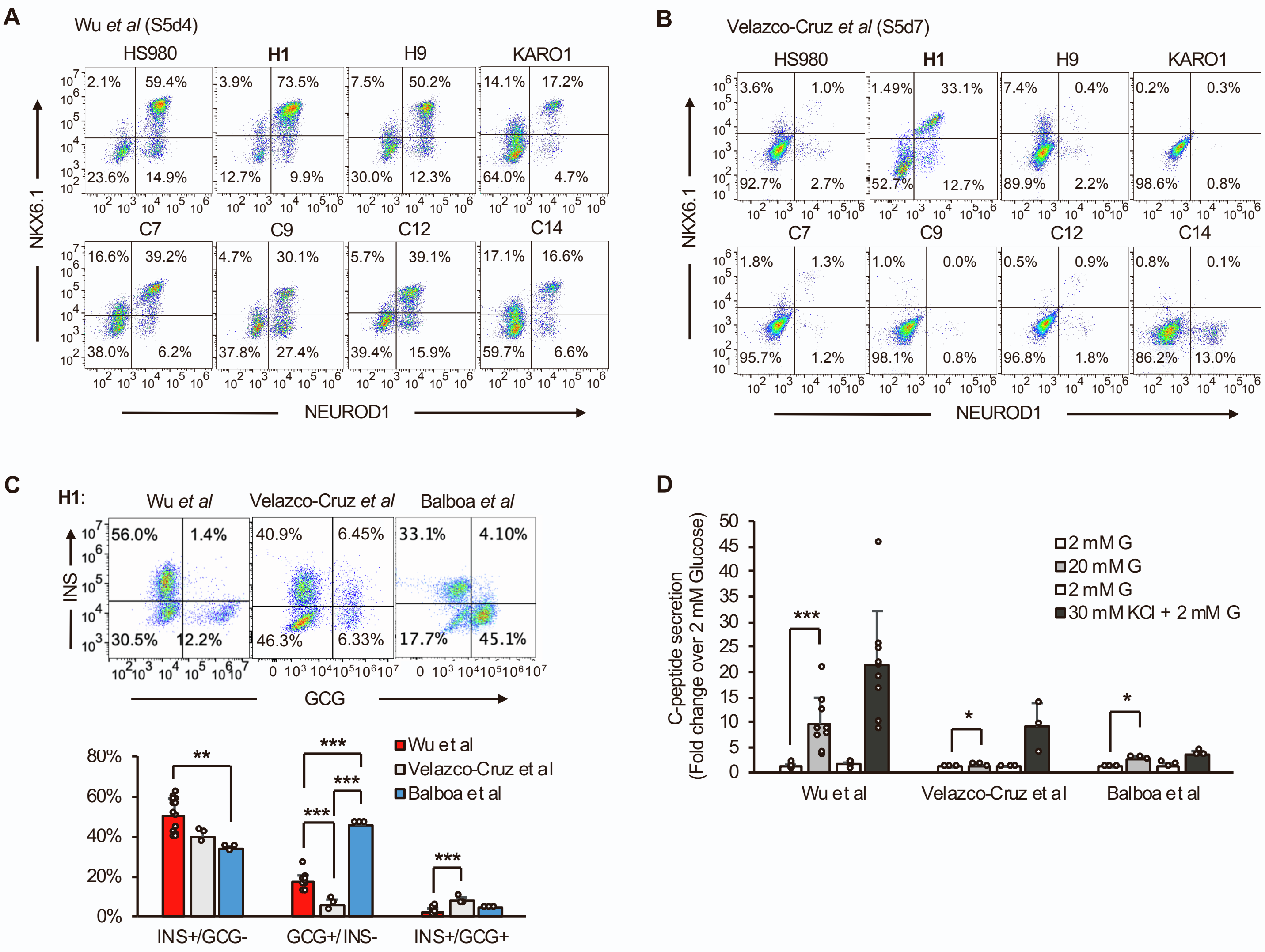

Figure S3. Comparison of three differentiation protocols, related to Figure 3.

Eight human ESC and iPSC lines were differentiated to S5 EP cells using (**A**) the short differentiation protocol as described in Methods (Wu *et al*), and (**B**) the 3D protocol described by Velazco-Cruz *et al* (Velazco-Cruz *et al*). Expressions of EP markers NKX6.1 and NEUROD1 were examined by flow cytometry at S5d4 (Wu *et al*) or S5d7 (Velazco-Cruz *et al*). Representative dot plots are shown. *n* = 7 for HS980 and H1, 3 for H9, KARO1 and C7, and 2 for C9, C12 and C14, all differentiated with Wu *et al.* *n* = 3 for H1, and 2 for HS980, H9, KARO1, C7, C9, C12, and C14, all differentiated with Velazco-Cruz *et al*. (**C** and **D**) H1 cells were further differentiated using the three differentiation protocols as indicated. (**C**) Expression of islet markers INS and GCG were analyzed by flow cytomtetry at S6. Representative dot plots (upper) and bar graphs (lower) are shown. Data are means ± SD, *n* = 13 (Wu et al), 3 (Velazco-Cruz and Balboa et al). One-way ANOVA, ** $p < 0.01$, *** $p < 0.001$. (**D**) Static GSIS showing fold change in c-peptide over 2 mM glucose. Data are means ± SD, *n* = 9 (Wu et al), 3 (Velazco-Cruz and Balboa et al); paired 2-tailed t-tests, * $p < 0.05$, ** $p < 0.01$, *** $p < 0.001$.

Figure S4

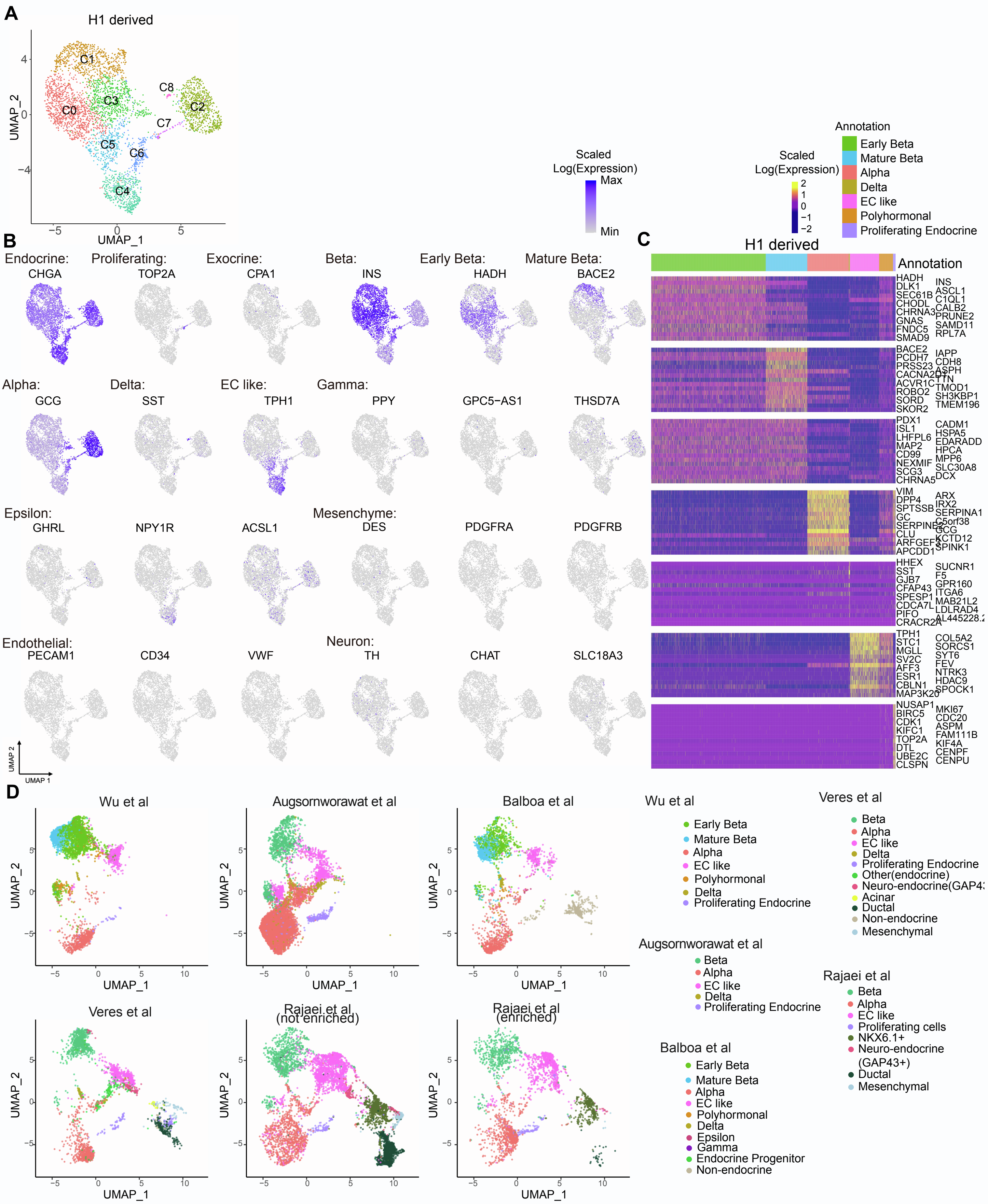

A
H1 derived
C0
C1
C2
C3
C4
C5
C6
C7
C8
UMAP_1
UMAP_2
B
Scaled Log(Expression)
Max
Min
Endocrine: CHGA
Proliferating: TOP2A
Exocrine: CPA1
Beta: INS
Early Beta: HADH
Mature Beta: BACE2
Alpha: GCG
Delta: SST
EC like: TPH1
Gamma: PPY
GPC5−AS1
THSD7A
Epsilon: GHRL
NPY1R
ACSL1
Mesenchyme: DES
PDGFRA
PDGFRB
Endothelial: PECAM1
CD34
VWF
Neuron: TH
CHAT
SLC18A3
C
H1 derived
Scaled Log(Expression)
Annotation
Early Beta
Mature Beta
Alpha
Delta
EC like
Polyhormonal
Proliferating Endocrine
D
Wu et al
Augsornworawat et al
Balboa et al
Veres et al
Rajaei et al (not enriched)
Rajaei et al (enriched)
Wu et al: Early Beta, Mature Beta, Alpha, EC like, Polyhormonal, Delta, Proliferating Endocrine
Veres et al: Beta, Alpha, EC like, Delta, Proliferating Endocrine, Other(endocrine), Acinar, Ductal, Non-endocrine, Mesenchymal
Augsornworawat et al: Beta, Alpha, EC like, Delta, Proliferating Endocrine
Rajaei et al: Beta, Alpha, EC like, Proliferating cells, NKX6.1+, Neuro-endocrine (GAP43+), Ductal, Mesenchymal
Balboa et al: Early Beta, Mature Beta, Alpha, EC like, Polyhormonal, Delta, Epsilon, Gamma, Endocrine Progenitor, Non-endocrine

Figure S4. Transcriptome analysis of SC-islets, related to Figure 4.

(**A**) UMAP projection showing the raw Seurat clusters of SC-islets derived from H1 cell lines at day 43 of differentiation under a resolution of 0.4. (**B**) UMAP plot of cells from H1 SC-islets displaying the expression of endocrine, proliferating, exocrine, beta, alpha, delta, EC-like, gamma, epsilon, Mesenchyme, Endothelial, and neuron markers. (**C**) Heatmap showing the expression of the top 15 marker genes in SC-islets from H1 cell lines at day 43 of differentiation. (**D**) Datasets from different protocols. UMAP projection of integrated datasets, segregated by different protocols, with cells colored according to their original annotations from each dataset.

Figure S5

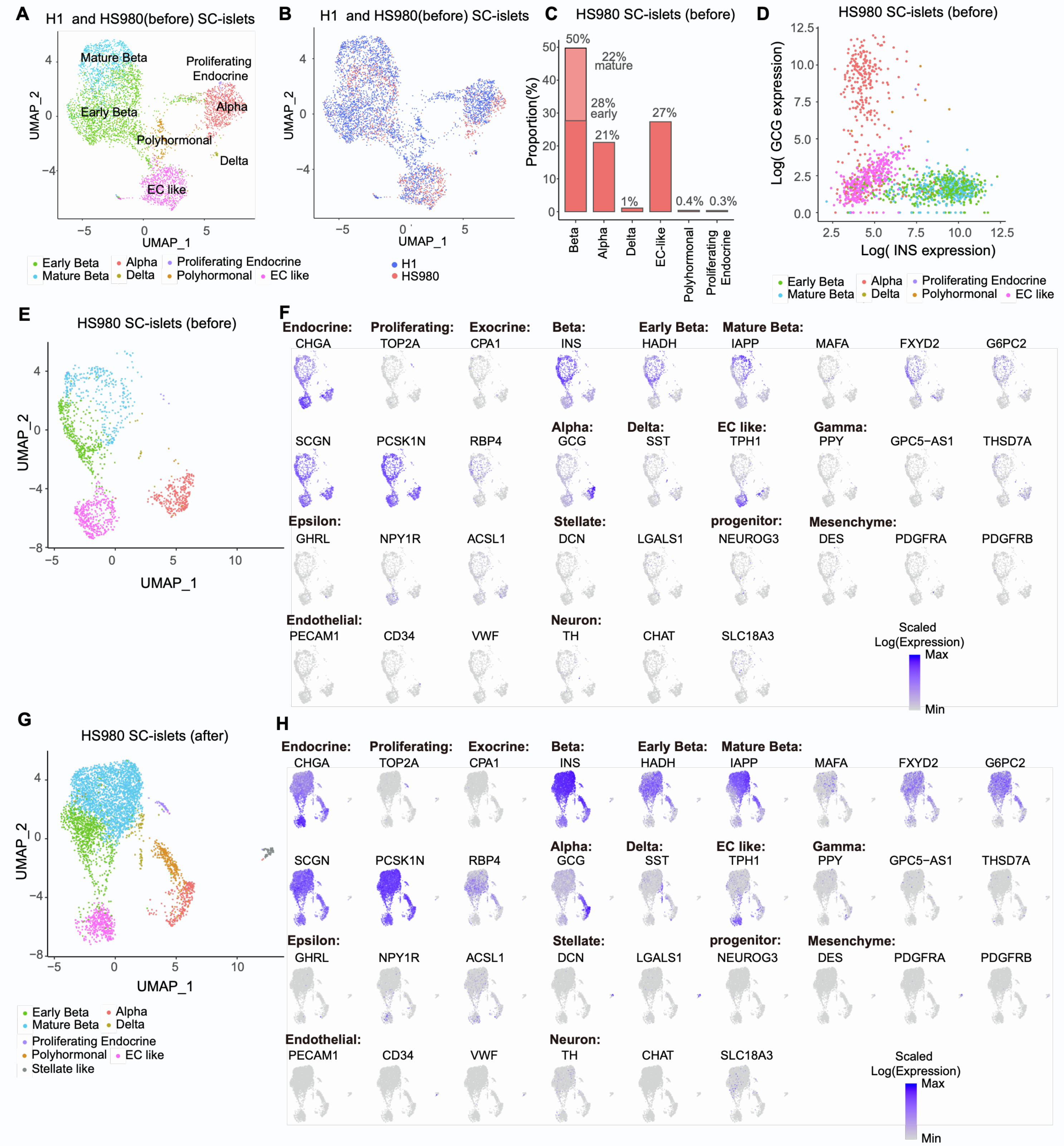

Figure S5. Transcriptome analysis of SC-islets before and after transplantation, related to Figure 6.

(**A** and **B**) UMAP projection showing the integration of SC-islets derived from H1 and HS980 cells before transplantation. Cells are colored by (**A**) annotation from each dataset and (**B**) cell source. (**C**) Bar plot showing the proportion of different cell types in SC-islets derived from HS980 cells before transplantation. (**D**) Dot plot showing expression of INS and GCG in different cell types in SC-islets derived from HS980 cells before transplantation. (**E** and **G**) UMAP projection showing the integration of SC-islets derived from HS980 cells before (**E**) and after (**G**) transplantation. (**F** and **H**) UMAP plots displaying the expression of endocrine, proliferating, exocrine, beta, alpha, delta, EC-like, gamma, epsilon, stellate, endocrine progenitor, mesenchyme, endothelial, and neuron markers in SC-islets before (**F**) and after (**H**) transplantation.

Figure S6

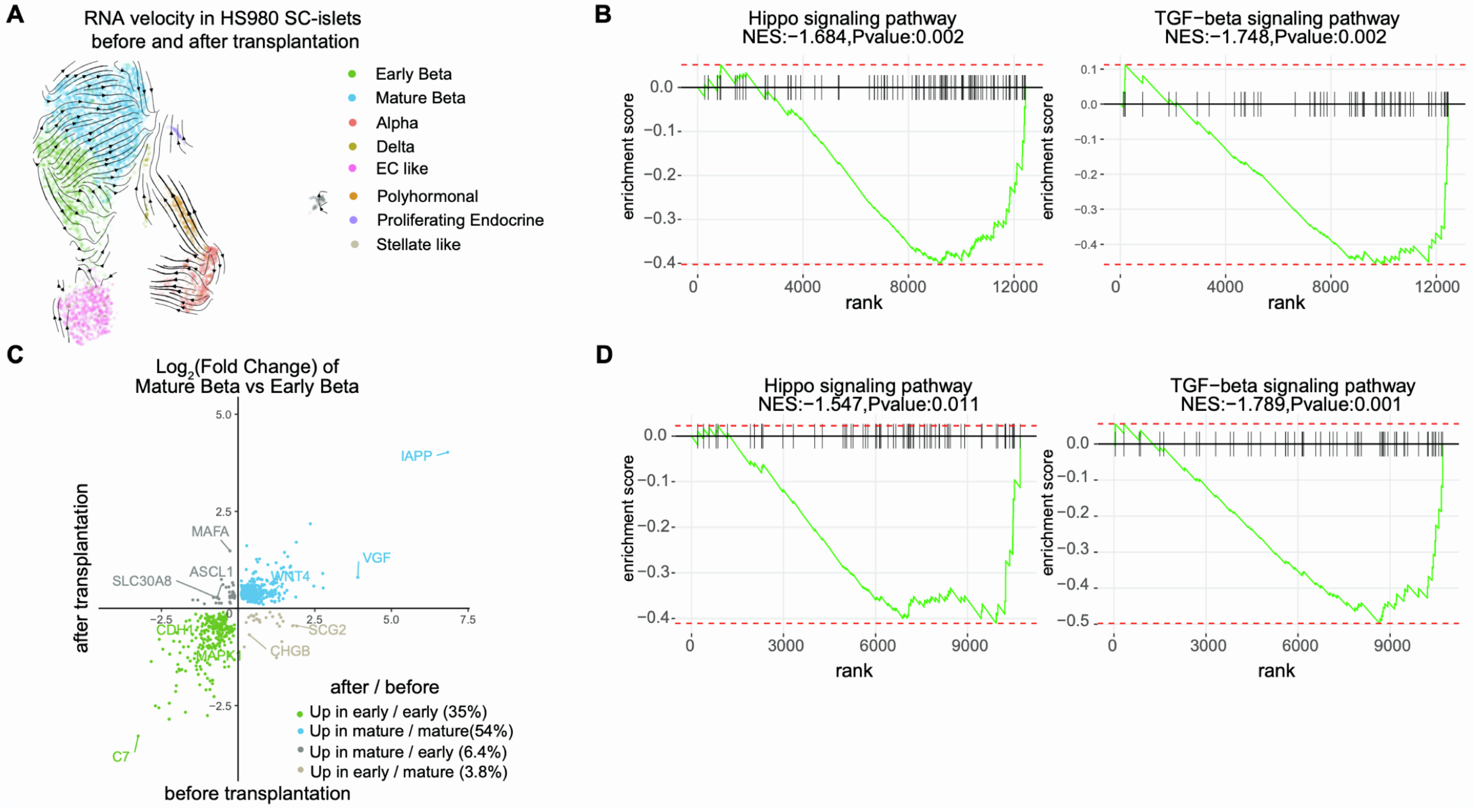

Figure S6. *In vivo* maturation of SC-islets, related to Figure 6.

(**A**) UMAP projection of the cell population post-transplantation with overlaid RNA velocity vectors. (**B** and **D**) Enrichment curves for GSEA results comparing mature beta and early beta cells in the HS980 cell line before (**B**) and after (**D**) transplantation. (**C**) Scatter plot showing the log2 fold change of the union set of differentially expressed genes (DEGs) between mature and early beta cells before transplantation (x-axis) and after transplantation (y-axis). Genes are colored based on the beta cell type (mature or early) in which they have higher expression before and after transplantation.

Table S1: Comparison of protocols for pancreatic islet differentiation.

			Wu *et al*	Hogrebe *et al* (*12, 13, 17, 18*)	Balboa *et al* (*11, 14, 16*)	Velazco-Cruz *et al* (*9*)	Rajaei *et al* (*15*)
2D planar or 3D suspension protocol			2D to 3D transition at S5	2D	2D to 3D transition at S4	3D	3D
SC-islet cell / initial stem cell			1.42 or 2.73	1 (*13*)	1 (*14*)	N/D	N/D
hPSC lines used for generation of SC-islets	hESC		**HS980, H1,** H9, KARO1	**HUES8**, H1	**H1**, H9	**HUES8**	**HUES8**, **RC9**
	WT iPSC		C7, C9, C12, C14	1013-4FA, 1016SeVA, AN1.1, BJFF.6	HEL24.3, HEL113, HEL118.3	1013-4FA, 1016SeVA	LUMC iPSC1, iPSC2, iPSC3
	Diabetic iPSC			1026-3FC, 1031SeVA, T2D001A		1019SeVA	
	Others			WS4			
Differentiation efficiencies	S4 NKX6.1$^+$		50-60%	>40% (*13*)	>60% (*14*)	N/D	N/D
	S5 NEUROD1$^+$		70-90%	N/D	N/D	N/D	N/D
	S6-7 Islet	INS$^+$/GCG$^-$	40-60%	30-55% (*12, 13*)	30-50% (*11, 14*)	40-50% (*9*)	56.4% (*15*)
		GCG$^+$/INS$^-$	15-20%	3-7% (*13*)	30-50% (*11, 14*)	<5% (*9*)	8.5% (*15*)
		INS$^+$/GCG$^+$	<5%	7-15% (*12, 13*)	<5% (*14*)	20-30% (*9*)	5.5% (*15*)
		Ki-67$^+$	1%	N/D	>1.5% (*11*)	N/D	N/D
In vitro GSIS	Static		6-12	2-3 (*13*)	N/D	3 (*9*)	1.2-1.6 (*15*)
	Dynamic	1st phase	16.1±6.29	3-9 (*12, 13*)	5-10 (*11*)	7.6±1.3 (*9*)	1.9±0.5 (*15*)
		2nd phase	6.1±1.07	2-3 (*12, 13*)	3-4 (*11*)	2.1±0.3 (*9*)	N/D
Transplantation studies	Transplantation site		ACE	Kidney (*12, 17, 18*)	Kidney (*11*), hind leg (*16*)	Kidney (*9*)	Kidney (*15*)
	Number of SC-islets or cells transplanted		600 SC-islets (1.2×10^6 cells)	$2\text{-}5 \times 10^6$ cells (*12, 17, 18*)	250-750 SC-islets (*11*)	5×10^6 cells (*9*)	N/D
	Reversal of preexisting diabetes		Yes	Yes (*12, 18*)	N/D	Yes (*9*)	N/D
	IPGTT and *in vivo* GSIS		Yes	Yes (*12, 18*)	Yes (*11*)	Yes (*9*)	Yes (*15*)
	Pancreatic exocrine cells		Not detected	Yes (*17*)	Yes (*16*)	Yes (*9*)	Yes (*15*)

Results from the herein presented protocol (Wu *et al*) are compared to what have been reported from four published protocols. The yield shows number of S6-7 SC-islet cells generated per initial stem cell. The identities of the hPSC lines from which SC-islets have been successfully generated are as shown. The main cell lines used for these studies are marked with **bold** text. The differentiation efficiency is presented as percentages of PP cells at the end of S4, EP cells at the end of S5, and endocrine and proliferative cells at the end of S6-7, as determined by flow cytometry and immunohistochemistry. The results for *in vitro* static and dynamic GSIS assays are presented as fold change over the low glucose level. The presence of pancreatic exocrine cells in graft is determined by immunohistochemistry. Data are estimated from the results presented in this manuscript and the previous reports (see Supplemental References). N/D, data not available.

Table S2: Conjugated antibodies for analysis with flow cytometry.

Conjugated antibodies	Source	Identifier	Dilution
Alexa Fluor 647 mouse anti-Insulin	BD Biosciences	565689	1:20
PE mouse anti-Glucagon	BD Biosciences	565860	1:20
Alexa Fluor 488 mouse anti-human Somatostatin	BD Biosciences	566032	1:20
PE mouse anti-NEUROD1	BD Biosciences	563001	1:20
Alexa Fluor 647 mouse anti-NKX6.1	BD Biosciences	563338	1:20
Alexa Fluor 488 mouse anti-PDX-1	BD Biosciences	562274	1:20
Alexa Fluor 488 mouse anti-Ki-67	BD Biosciences	561165	1:20
V450 mouse anti-Ki-67	BD Biosciences	561281	1:20

Table S3: Primary antibodies for analysis with immunofluorescence.

Primary antibodies	Source	Identifier	Application	Dilution
goat anti-human PDX-1	R&D systems	AF2419	ICC	1:300
guinea pig anti-PDX1	abcam	ab47308	ICC	1:200
mouse anti-NKX6.1	DSHB	F55A12-s	ICC	1:100
goat anti-human/mouse NEUROD1	R&D systems	AF2746	ICC	1:100
sheep anti-human NGN3	R&D systems	AF3444	ICC	1:100
guinea pig anti-C-Peptide	abcam	ab30477	ICC	1:100
rat anti-C-Peptide	DSHB	GN-ID4-s	ICC	1:50
mouse anti-Glucagon	Sigma	G2654	ICC	1:1000
rabbit anti-Somatostatin	Sigma	332A-1	ICC	1:500
rabbit anti-SLC18A1	Sigma	HPA063797	ICC	1:500
Goat anti-human SOX9	R&D systems	AF3075	IHC	1:40
guinea pig anti-Insulin	Dako Agilent	IR00261-2	IHC	1:10
rabbit anti-Glucagon	BioGenex	PU039-5UP	IHC	1:300
rat anti-human Somatostatin	Bio-Rad	8330-0009	IHC	1:400

Supplemental Methods

hPSC culture

hESC line HS980 (KIe033-A) was derived under xeno-free, defined conditions with informed donor consent (Swedish Ethical Review Authority 2011/745:31/3)[1]. hESC line KARO1 (KIe034-A) was generated and banked under GMP guidelines[2]. WA01/H1 (WAe001-A) and WA09/H9 (WAe009-A) were purchased from WiCell. Human iPSC lines CTRL-7-II (C7), CTRL-9-II (C9), CTRL-12-I (C12), and CTRL-14-II (C14) were obtained from the Karolinska Institute iPSC core facility[3]. Mycoplasma testing was performed every 6 months, and pluripotency marker expression was confirmed by flow cytometry.

hPSCs were maintained in NutriStem hPSC XF Medium (Biological Industries, 05-100-1A) on culture plates coated with 10 µg/mL human recombinant laminin (LN) -521 (BioLamina, LN521). Cultures were kept at 37°C with 5% CO_2 and 5% O_2 and passaged every 3–5 days at a density of 15,000–24,000 cells/cm². For passaging, cells were washed with PBS (Thermo Fisher, 14190169), incubated with TrypLE Select (Thermo Fisher, A1285901) for 4–5 min at 37°C, gently dissociated, centrifuged at 300 g for 5 min, resuspended in fresh NutriStem, and re-plated onto newly coated plates.

In vitro pancreatic islet differentiation of hPSCs

The pancreatic islet differentiation protocols described here were modified from previously published protocols[4-10].

The hESC lines were seeded onto LN-521 coated cell culture plates at 18000 - 24000 cells/cm^2 in NutriStem hPSC XF medium. The human iPSC lines were seeded onto LN-521 coated cell culture plates at 15000 cells/cm^2 in NutriStem hPSC XF medium supplemented with 5 µM ROCK inhibitor Y-27632 (Bio-Techne, 1254). Next day, the medium was changed to NutriStem hPSC XF medium without Y-27632. The pancreatic differentiation was initiated four days later, resulting in 90-100% confluency. The differentiating cell cultures were maintained in a 37°C incubator with 5% CO_2, 20% O_2 and 100% humidity. The differentiation can be divided into six stages (S1-S6), and media used for each stage were as follows:

S1 media: MCDB131 (Thermo Fisher; 10372019) + 25 mM NaHCO3 (Sigma; S6297) + 1X GlutaMAX (Thermo Fisher; 35050038) + 50 U/ml Penicillin-Streptomycin (Thermo Fisher; 15140122) + 2.5 mM D-Glucose (8 mM final concentration, Sigma; G8769) + 0.2% or 0.5% Fatty Acid Free Bovine Serum Albumin (FAF-BSA, Sigma; A8806).

S2 media: MCDB131 + 25 mM NaHCO3 + 1X GlutaMAX + 50 U/ml Penicillin-Streptomycin + 2.5 mM D-Glucose (8 mM final concentration) + 0.2% or 0.5% FAF-BSA + 0.25 mM Vitamin C (Sigma; A4544).

S3-4 media: MCDB131 + 25 mM NaHCO3 + 1X GlutaMAX + 50 U/ml Penicillin-Streptomycin + 2.5 mM D-Glucose (8 mM final concentration) + 0.5% FAF-BSA + 0.25 mM Vitamin C + 1:200 ITS-X (Thermo Fisher; 51500056).

S5 media: MCDB131 + 25 mM NaHCO3 + 1X GlutaMAX + 50 U/ml Penicillin-Streptomycin + 14.5 mM D-Glucose (20 mM final concentration) + 0.5% FAF-BSA + 1:200 ITS-X + 10 µM ZnSO4 (Sigma; Z0251) + 10 µg/ml Heparin (Sigma; H3149).

S6 media: CMRL (Thermo Fisher; 11530037) + 14 mM NaHCO3 + 1X GlutaMAX + 50 U/ml Penicillin-Streptomycin + 14.5 mM D-Glucose (20 mM final concentration) + 1% FAF-BSA + 1:200 ITS-X (for three weeks) + 10 µM ZnSO4 + 10 µg/ml Heparin + 1X NEAA (Thermo Fisher; 11140035).

The short differentiation protocol:

S1 definitive endoderm (3 days): Undifferentiated hPSCs were rinsed once with D-PBS with Ca^{2+} and Mg^{2+} (Thermo Fisher; 14040091) and then induced with 5 µM CHIR99021 (Tocris; 4423) and 100 ng/ml Activin A (R&D; 338-AC) for 24 hours in S1 media. For the next 2 days the cells were fed every day with S1 media containing only 100 ng/ml Activin A. The concentrations of FAF-BSA in S1 media were 0.2% for HS980, C9, C12 and C14 cells, and 0.5% for H1, H9, KARO1, and C7 cells.

S2 primitive gut tube (3 days): cells were induced with 50 ng/ml KGF (R&D; 251-KG) in S2 media for 3 days. The concentrations of FAF-BSA were 0.2% for HS980, C9, C12 and C14 cells, and 0.5% for H1, H9, KARO1, and C7 cells.

S3 posterior foregut (1 day): cells were induced with 50 ng/ml KGF, 2 µM Retinoic acid (Sigma; R2625), 0.25 µM SANT-1 (Sigma; S4572), 0.5 µM PDBu (Tocris; 4153), and 200 nM LDN193189 (Tocris; 6053) in S3-4 media for 24 hours.

S4 pancreatic progenitor (3 days): cells were induced with 50 ng/ml KGF, 100 ng/ml EGF (R&D; 236-EG), 5 ng/ml Activin A, 10 mM Nicotinamide (Sigma; N0636), 100 nM Retinoic acid, 0.25 µM SANT-1, 0.5 µM PDBu, and 200 nM LDN193189 in S3-4 media for 3 days. To analyze the effect of stage 4 durations, cells were also differentiated for 1-5 days during this step.

S5 endocrine progenitor (5 days): cells were induced with 20 ng/ml Betacellulin (R&D; 261-CE), 100 nM Retinoic acid, 0.25 µM SANT-1, 100 nM GSI-XX (Sigma; 565789), 10 µM ALK5 inhibitor II (Cayman Chemical; 14794), 1 µM GC-1 (Tocris; 4554), and 100 nM LDN193189 in S5 media for 4 days.

Four days into S5 (S5d4), cells were rinsed once with PBS, treated with StemPro Accutase cell dissociation reagent (Thermo Fisher; A1110501) for 10 minutes at 37°C, and then dissociated into single cells in S5 media by pipetting 10-15 times using a P1000 pipette. Single cells were pelleted by centrifugation at 300 g for 5 minutes, and then resuspended at 1.0-1.5 x 10^6 cells/ml in S5 media supplemented with 10 μM H1152 (Tocris; 2414) and the other factors. To generate islet-like aggregates, cells were transferred to ultra-low attachment 6-well plates (Corning; 3471), totally 4-6 x 10^4 cells in 4 ml per well, and incubated overnight on an orbital shaker (Infors HT Celltron) at 95 rpm, Ø 25 mm, in the incubator.

To investigate the effects of ROCK inhibitor H1152 on cell survival and aggregate formation, different concentrations of H1152, from 0 to 10 μM, were added to a single cell suspension for 24 hours. Next day, the cell aggregates were collected, rinsed once in PBS, treated with Accutase for 10 minutes at 37°C, and then dissociated into single cells in S5 media by pipetting 10-15 times using a P1000 pipette. The cell number was counted to determine the aggregation efficiency using the equation:

Aggregation efficiency = Nf / Ni

where Ni is the initial number of single cells at S5d4 and Nf is the number of cells in aggregates at S5d5.

S6 pancreatic islets (about 4 weeks): the cell aggregates were maintained in S6 media further supplemented with 10 μM H1152, 1 μM GC-1, 10 μM Trolox (Merck Millipore; 648471), and 1 mM N-acetyl-L-cysteine (Sigma; A9165). ITS-X and H1152 were removed from the media after three weeks. The aggregates were kept on an orbital shaker at 95 rpm in the incubator.

The media were changed every day from stage 1 to 5, and every 2-3 days during stage 6.

The long differentiation protocol:

hPSCs were differentiated using the same factors and media as the short differentiation protocol described above. The durations for S3 and S4 were 2 and 5 days respectively.

Comparison to published differentiation protocols

To benchmark our LN-521-based short differentiation protocol, H1 cells were differentiated in parallel using published protocols from Velazco-Cruz *et al.*[9] and Balboa *et al.*[11], following the original procedures. Endocrine composition was assessed by flow cytometry (see Methods), and functional maturation by static GSIS (see below). In addition, Table S1 provides a structured overview of several published SC-islet differentiation protocols, including 2D/3D format, SC-islet yield, hPSC lines tested, differentiation efficiencies at stages S4–S6/7, static and dynamic GSIS, and transplantation outcomes, based on previously published literature[9,11-18].

Dissociation of SC-islet and counting of cell number

The S6 SC-islets were counted under a brightfield microscope. S6 SC-islets were rinsed in PBS and incubated with Accutase for 12–15 min on an orbital shaker (95 rpm, 37°C). Islets were dissociated to single cells by pipetting 15× with a P1000 pipette, resuspended in S6 medium, pelleted at 300 g for 5 min, and washed in PBS. Cell numbers were quantified using a MOXI Z Mini Automated Cell Counter (ORFLO, MXZ001).

Freezing and thawing of stage 5 endocrine progenitor cells

At S5d4, cells were dissociated to single cells, pelleted at 300 g for 5 min, and resuspended at 1×10^7 cells/mL in cold STEM-CELLBANKER GMP solution (Amsbio, 11924). Suspensions (1–1.5 mL) were aliquoted into Nunc cryogenic tubes (Thermo Fisher, 377267) and cooled to –80°C using a Mr. Frosty container (Thermo Fisher, 5100-0001). Alternatively, cells can be cooled at 1°C/min using a programmable cooling unit. For long-term storage, vials were transferred to liquid nitrogen.

Frozen S5 cells were retrieved from liquid nitrogen and rapidly thawed at 37°C. Each 1 mL suspension was diluted into 5 mL pre-warmed S5 medium, centrifuged at 300 g for 5 min, and resuspended at $1.0–1.5 \times 10^6$ cells/mL in complete S5 medium. Differentiation then proceeded as described above.

Static *in vitro* glucose stimulated insulin secretion (GSIS)

SC-islets (20–30 per assay) at S6w4 were incubated overnight in S6 medium lacking ITS-X and without additional glucose (final glucose concentration 5 mM). The next day, SC-islets were transferred to 24-well ultra-low attachment plates (Corning; 3473) and washed twice with 2 mL Krebs buffer containing 129 mM NaCl, 4.8 mM KCl, 2.5 mM $CaCl_2$, 1.2 mM $MgSO_4$, 1 mM Na_2HPO_4, 1.2 mM KH_2PO_4, 5 mM $NaHCO_3$, 10 mM HEPES, and 0.1% fatty acid–free BSA (FAF-BSA). Islets were pre-incubated in 2 mL Krebs buffer supplemented with 2 mM glucose for 2 hours to remove residual insulin.

SC-islets were then sequentially incubated for 30 min in 2 mL Krebs buffer under the following conditions:
(1) 2 mM glucose (low glucose), (2) 20 mM glucose (high glucose), (3) 2 mM glucose (low glucose), and (4) 2 mM glucose plus 30 mM KCl (depolarization). Between each incubation, islets were washed with 2 mL Krebs buffer. After each incubation, 500 µL supernatant was collected and stored for analysis.

Following the KCl challenge, SC-islets were dissociated with Accutase for 15 min and total cell numbers were determined using an ORFLO MOXI Z cell counter. Secreted insulin was quantified using a human C-peptide ELISA kit (R&D; DICP00). C-peptide

secretion was normalized to total cell number and reported as pmol C-peptide released per 1×10^3 cells or as fold change. Samples not analyzed on the same day were stored at −80°C.

Dynamic GSIS assay

Dynamic glucose-stimulated insulin secretion (GSIS) was performed using a Biorep PERI-4.2 perifusion system (Biorep Technologies). Fifty handpicked SC-islets were loaded into 0.27 ml columns containing Bio-Gel P4 polyacrylamide beads (Bio-Rad, 1504128). Perifusion buffer consisted of 129 mM NaCl, 4.8 mM KCl, 2.5 mM $CaCl_2$, 1.2 mM $MgSO_4$, 1 mM Na_2HPO_4, 1.2 mM KH_2PO_4, 5 mM $NaHCO_3$, 10 mM HEPES, and 0.1% FAF-BSA, supplemented with either 2.8 mM (low) or 16.8 mM (high) glucose. The buffer was delivered at a flow rate of 50 µl/min at 37°C. Following a 90-min pre-equilibration in 2.8 mM glucose, SC-islets were sequentially exposed to: 2.8 mM glucose for 12 min, 16.8 mM glucose for 36 min, and 2.8 mM glucose for 20 min. Effluent was collected every 2 min, and insulin concentrations were quantified using the AlphaLISA insulin detection kit (AL3184). A 6-min tubing delay was corrected for in data presentation.

Single-cell RNA sequencing sample preparation

S6 SC-islets from H1 and HS980 cells were collected on day 43 of differentiation. Islets were rinsed twice in PBS and incubated with TrypLE (15 min, 37°C, orbital shaker) to dissociate into single cells by pipetting. Cells were centrifuged at 300 × g for 5 min, resuspended in PBS + 0.04% BSA (Sigma, A7284), and filtered through a 40 µm strainer (VWR, 732-2760).

For post-transplant samples, mouse iris containing SC-islet grafts was excised at 20 weeks post-transplantation. Surrounding tissue was trimmed and washed twice in PBS. Samples were digested in PBS containing 2 mg/ml Collagenase IV (ThermoFisher, 17104019), 10 U/ml DNase I (Merck, 04716728001), and 1 mg/ml Papain (Sigma, 76216) for 50 min at 37°C in a ULA 6-well plate, with 15 pipetting strokes every 10 min. Enzymatic activity was quenched with PBS + 10% FBS. Cells were filtered (40 µm), pelleted, and resuspended at 0.8×10^6 cells/ml in PBS + 1% BSA. Viability (~87%) and total cell numbers were determined using Trypan blue (0.4%, ThermoFisher, 15250061) and a hemocytometer. For scRNA-seq, 5×10^3 H1, 2.5×10^3 HS980, and 1×10^4 graft-derived cells were loaded per library.

Single-cell RNA sequencing analysis

Single-cell libraries were prepared using 10x Genomics Chromium Next GEM Single Cell 3′ Reagent Kits v3.1 (CG000315 or CG000388), with optional Cell Multiplexing Oligo labeling (CG000391). Libraries were sequenced on an Illumina NextSeq 2000 at ~4×10^4 reads per cell. FASTQ files and feature-barcode matrices were generated using Cell Ranger 6.1.1 (*cellranger count*, default settings). Reads were aligned to the human reference genome GRCh38.98 (v3.0.0). To exclude mouse cells in graft samples,

reads were also mapped to the mm10 reference genome (gex-mm10-2020-A, 10x Genomics), and only cells expressing ≥2.25-fold more human than mouse genes (based on pre-transplant controls) were retained. Downstream analysis was performed in Seurat v5.1.0 (R v4.3.3), keeping high-quality cells with 1,000–7,000 detected genes (nFeature) and <25% mitochondrial genes (percent.mito), yielding 3,940 H1, 1,485 HS980, and 4,461 grafted cells.

Single-cell RNA-seq data from H1 SC-islets were quality-controlled, excluding mitochondrial genes, and analyzed using the standard Seurat pipeline[19]. Genes expressed in ≥5 cells were log-normalized using the computeSumFactors function in R scran package (v1.30.2)[20]. The 2,000 most variable genes were identified using the vst method with FindVariableFeatures function. The top 25 principal components (PC) were computed (RunPCA) and used for uniform manifold approximation and projection (UMAP) dimensional reduction (RunUMAP). Clusters were identified with default Louvain clustering (FindClusters). Cluster stability was assessed with the R package clustree v0.4.3[21] across resolution values 0.2–0.8, with 0.4 resolving delta and proliferating cells. Cell identities were assigned using canonical markers. Datasets from HS980 before and after transplantation were log-normalized, rescaled with the multiBatchNorm function in batchelor package (v1.18.1)[22] to normalize size factors, and integrated using the RunFastMNN function in SeuratWrappers package (v0.3.5) with 2,000 anchor features and 25 PCs. Clustering and UMAP were performed on the integrated PCs. Differential gene expression between mature and early beta cells was assessed using the two-sided Wilcoxon test (FindMarkers). Genes with Bonferroni-adjusted $P < 0.05$, $\log_2 FC > 0.25$, and expression in >25% of cells were considered differentially expressed (DEG). Functional enrichment of DEGs and gene set enrichment analysis (GSEA) of all genes were performed using the "enricher" and "fgsea" functions from the clusterProfiler package (v3.18.1)[23] and fgsea package(v1.32.2) (http://biorxiv.org/content/early/2016/06/20/060012.), with KEGG annotations from EnrichR database[24]. To compare datasets, integration was performed with fastMNN, and integrated (MNN) PCs were used to construct UMAP, identify neighbors, and define clusters with Seurat. β cell populations before and after transplantation were compared using average expression and a maturation signature calculated from gene INS, G6PC2, HOPX, UCN, IAPP, CPE, SIX3, BACE2, MAFA, and FXYD2. RNA velocity in HS980 and grafted cells was calculated with Python script velocyto.py from scvelo package (v0.1.25)[25,26] and projected onto UMAP using scv.pl.velocity_embedding_stream with default parameters.

Comparison to established pancreatic differentiation protocols

Datasets from Augsornworawat *et al*., 2020 (S6, GSE151117), Balboa *et al*., 2022 (S7w3 day 20, GSE167880), and Veres *et al*., 2019 (S6w4, GSE114412), and Rajaei *et al*., 2025 (EGAS50000000905) were analyzed, totaling ~3×10^4 cells. Data from Augsornworawat, Balboa, and Rajaei were generated using the 10x Genomics platform[11,15,17], while Veres used inDrops[27]. To compare these protocols, batch-to-batch correction with multiBatchNorm was applied, followed by integration with fastMNN using 2500 anchor features and top 25 PCs. Downstream analysis followed the workflow described above (FindClusters resolution = 0.6).

Proliferation scores (Fig. 4I) were computed using AddModuleScore with MKI67, CDK1, TOP2A, CCNB2, CCNA2, and PBK. β-cell maturation scores (Fig. 6D) were calculated using AddModuleScore with INS, G6PC2, HOPX, UCN3, IAPP, CPE, SIX3, BACE2, MAFA, and FXYD2.

Transplantation studies

All animal procedures were approved by Regional Ethical Committee at Karolinska Institutet. Six to seven week old NOD-scid gamma mice (NSG, Jackson Laboratories) were maintained under controlled temperature and humidity on a 12 hours light/dark cycle with ad libitum chow diet and water.

Diabetes was induced in 8 week old NSG mice by intraperitoneal (i.p.) injection of streptozotocin (STZ; Sigma, S0130) at 60 mg/kg body weight for four consecutive days. Non-fasting blood glucose was monitored throughout the study using an Accu-Chek blood glucose meter (Roche). Blood samples were collected from the tail vein monthly in Microvette CB 300 EDTA K2E tubes (Sarstedt, 16.444). Human and mouse c-peptide levels were quantified using species-specific ELISA kits (Crystal Chem, 80954 and 90050).

Transplantation of SC-islets into ACE

SC-islets differentiated from HS980 cells, 280–300 per eye, were transplanted into the ACE of STZ- treated diabetic mice. Mice were anesthetized with isoflurane and secured with stereotaxic head holder and eye holder. Under a stereo microscope (M80, Leica), the cornea was punctured with a 23G needle, and SC-islets were delivered into the ACE using a glass microcannula connected via polyethylene tubing to a 0.5 mL syringe (Hamilton, USA). Oculentum simplex (APL, Sweden) was applied to prevent corneal dryness and inflammation. Temgesic (Indivior, Ireland) was administered subcutaneously (s.c.) at 0.1 μg/g body weight to relieve postoperative pain. Transplanted diabetic mice received long-acting insulin (Insulatard Penfill, Novo Nordisk) at 0.05–0.15 IU s.c. from day 4-5 post-transplant for 45-56 days.

Intraperitoneal glucose tolerance test (IPGTT)

IPGTTs were performed on 5 hours fasted mice. Blood glucose was measured at 0 min, and 15, 30, 60, and 120 min after glucose load (3.5 g/kg body weight, i.p.). Blood for c-peptide quantification was collected at 0, 30, 60, and 120 min, and plasma stored at −80°C. C-peptide levels were measured as described above.

Supplemental References

1. Rodin S, Antonsson L, Niaudet C, et al. Clonal culturing of human embryonic stem cells on laminin-521/E-cadherin matrix in defined and xeno-free environment. *Nat Commun*. 2014;5:3195. doi:10.1038/ncomms4195

2. Main H, Hedenskog M, Acharya G, Hovatta O, Lanner F. Karolinska Institutet Human Embryonic Stem Cell Bank. *Stem Cell Res*. May 2020;45:101810. doi:10.1016/j.scr.2020.101810

3. Plaza Reyes A, Petrus-Reurer S, Padrell Sánchez S, et al. Identification of cell surface markers and establishment of monolayer differentiation to retinal pigment epithelial cells. *Nat Commun*. Mar 30 2020;11(1):1609. doi:10.1038/s41467-020-15326-5

4. D'Amour KA, Bang AG, Eliazer S, et al. Production of pancreatic hormone-expressing endocrine cells from human embryonic stem cells. *Nat Biotechnol*. Nov 2006;24(11):1392-401. doi:10.1038/nbt1259

5. Kroon E, Martinson LA, Kadoya K, et al. Pancreatic endoderm derived from human embryonic stem cells generates glucose-responsive insulin-secreting cells in vivo. *Nat Biotechnol*. Apr 2008;26(4):443-52. doi:10.1038/nbt1393

6. Rezania A, Bruin JE, Arora P, et al. Reversal of diabetes with insulin-producing cells derived in vitro from human pluripotent stem cells. *Nat Biotechnol*. Nov 2014;32(11):1121-33. doi:10.1038/nbt.3033

7. Pagliuca FW, Millman JR, Gürtler M, et al. Generation of functional human pancreatic β cells in vitro. *Cell*. Oct 2014;159(2):428-39. doi:10.1016/j.cell.2014.09.040

8. Nostro MC, Sarangi F, Yang C, et al. Efficient generation of NKX6-1+ pancreatic progenitors from multiple human pluripotent stem cell lines. *Stem Cell Reports*. Apr 2015;4(4):591-604. doi:10.1016/j.stemcr.2015.02.017

9. Velazco-Cruz L, Song J, Maxwell KG, et al. Acquisition of Dynamic Function in Human Stem Cell-Derived β Cells. *Stem Cell Reports*. 02 2019;12(2):351-365. doi:10.1016/j.stemcr.2018.12.012

10. Cogger KF, Sinha A, Sarangi F, et al. Glycoprotein 2 is a specific cell surface marker of human pancreatic progenitors. *Nat Commun*. 08 2017;8(1):331. doi:10.1038/s41467-017-00561-0

11. Balboa D, Barsby T, Lithovius V, et al. Functional, metabolic and transcriptional maturation of human pancreatic islets derived from stem cells. *Nat Biotechnol*. Jul 2022;40(7):1042-1055. doi:10.1038/s41587-022-01219-z

12. Hogrebe NJ, Augsornworawat P, Maxwell KG, Velazco-Cruz L, Millman JR. Targeting the cytoskeleton to direct pancreatic differentiation of human pluripotent stem cells. *Nat Biotechnol*. 04 2020;38(4):460-470. doi:10.1038/s41587-020-0430-6

13. Hogrebe NJ, Maxwell KG, Augsornworawat P, Millman JR. Generation of insulin-producing pancreatic β cells from multiple human stem cell lines. *Nat Protoc*. Sep 2021;16(9):4109-4143. doi:10.1038/s41596-021-00560-y

14. Barsby T, Ibrahim H, Lithovius V, et al. Differentiating functional human islet-like aggregates from pluripotent stem cells. *STAR Protoc*. Dec 16 2022;3(4):101711. doi:10.1016/j.xpro.2022.101711

15. Rajaei B, Garcia AM, Juksar J, et al. Clinically compliant enrichment of human pluripotent stem cell-derived islets. *Sci Transl Med*. Apr 02 2025;17(792):eadl4390. doi:10.1126/scitranslmed.adl4390

16. Lithovius V, Lahdenpohja S, Ibrahim H, et al. Non-invasive quantification of stem cell-derived islet graft size and composition. *Diabetologia*. Jun 14 2024;doi:10.1007/s00125-024-06194-5

17. Augsornworawat P, Maxwell KG, Velazco-Cruz L, Millman JR. Single-Cell Transcriptome Profiling Reveals β Cell Maturation in Stem Cell-Derived Islets after Transplantation. *Cell Rep*. Aug 25 2020;32(8):108067. doi:10.1016/j.celrep.2020.108067

18. Maxwell KG, Kim MH, Gale SE, Millman JR. Differential Function and Maturation of Human Stem Cell-Derived Islets After Transplantation. *Stem Cells Transl Med*. Mar 31 2022;11(3):322-331. doi:10.1093/stcltm/szab013

19. Hao Y, Hao S, Andersen-Nissen E, et al. Integrated analysis of multimodal single-cell data. *Cell*. Jun 24 2021;184(13):3573-3587.e29. doi:10.1016/j.cell.2021.04.048

20. Lun AT, McCarthy DJ, Marioni JC. A step-by-step workflow for low-level analysis of single-cell RNA-seq data with Bioconductor. *F1000Res*. 2016;5:2122. doi:10.12688/f1000research.9501.2

21. Zappia L, Oshlack A. Clustering trees: a visualization for evaluating clusterings at multiple resolutions. *Gigascience*. Jul 01 2018;7(7)doi:10.1093/gigascience/giy083

22. Haghverdi L, Lun ATL, Morgan MD, Marioni JC. Batch effects in single-cell RNA-sequencing data are corrected by matching mutual nearest neighbors. *Nat Biotechnol*. Jun 2018;36(5):421-427. doi:10.1038/nbt.4091

23. Yu G, Wang LG, Han Y, He QY. clusterProfiler: an R package for comparing biological themes among gene clusters. *OMICS*. May 2012;16(5):284-7. doi:10.1089/omi.2011.0118

24. Kuleshov MV, Jones MR, Rouillard AD, et al. Enrichr: a comprehensive gene set enrichment analysis web server 2016 update. *Nucleic Acids Res*. Jul 08 2016;44(W1):W90-7. doi:10.1093/nar/gkw377

25. La Manno G, Soldatov R, Zeisel A, et al. RNA velocity of single cells. *Nature*. Aug 2018;560(7719):494-498. doi:10.1038/s41586-018-0414-6

26. Bergen V, Lange M, Peidli S, Wolf FA, Theis FJ. Generalizing RNA velocity to transient cell states through dynamical modeling. *Nat Biotechnol*. Dec 2020;38(12):1408-1414. doi:10.1038/s41587-020-0591-3

27. Veres A, Faust AL, Bushnell HL, et al. Charting cellular identity during human in vitro β-cell differentiation. *Nature*. 05 2019;569(7756):368-373. doi:10.1038/s41586-019-1168-5
