# Supplementary material for: An optimized protocol for efficient derivation of pancreatic islets from multiple human pluripotent stem cell lines
Source: Stem Cell Reports. 2026 Apr 16;21(5):102892. doi: 10.1016/j.stemcr.2026.102892 (PMC13163220; doi:10.1016/j.stemcr.2026.102892)
Supplement: Document S2. Article plus supplemental information [file mmc2.pdf]

STEM CELL REPORTS
ISSCR
Article
OPEN ACCESS

An optimized protocol for efficient derivation of pancreatic islets from multiple human pluripotent stem cell lines

Siqin Wu,[1,2,3,9,*] Shivam Chandel,[1,2,8] Galyna Bryzgalova,[4,8] Paschalis Efstathopoulos,[1,2,3] Kelly Blust,[5] Cheng Zhao,[1,2] Eda Erbil,[1,2] Anna Falk,[6,7] My Hedhammar,[5] Per-Olof Berggren,[4,*] and Fredrik Lanner[1,2]
[1]Department of Clinical Sciences, Intervention and Technology, Karolinska Institutet, 171 77 Stockholm, Sweden
[2]Gynecology and Reproductive Medicine, Karolinska Universitetssjukhuset, 141 86 Stockholm, Sweden
[3]Spiber Technologies AB, AlbaNova University Center, 106 91 Stockholm, Sweden
[4]The Rolf Luft Research Center for Diabetes and Endocrinology, Karolinska Institutet, 171 76 Stockholm, Sweden
[5]Division of Protein Technology, KTH Royal Institute of Technology, Roslagstullsbacken 21, 106 91 Stockholm, Sweden
[6]Department of Neuroscience, Karolinska Institutet, 171 65 Solna, Sweden
[7]Neural Stem Cells, Department of Experimental Medical Science, Lund Stem Cell Center, Lund University, 221 84 Lund, Sweden
[8]These authors contributed equally
[9]Lead contact
*Correspondence: siqinw@gmail.com (S.W.), per-olof.berggren@ki.se (P.-O.B.)
https://doi.org/10.1016/j.stemcr.2026.102892

SUMMARY

The success of cell therapy for type 1 diabetes (T1D) depends on reliable differentiation of stem cells into functional pancreatic islets. Current protocols produce stem cell-derived islets (SC-islets) that contain non-endocrine cells and show limited maturity. We developed a robust protocol that generates functional SC-islets from all eight tested human pluripotent stem cell (hPSC) lines. Differentiation to the endocrine progenitor (EP) stage on 2D laminin-521 is improved by shortening the prior pancreatic progenitor (PP) stage. Notably, allowing EP cells to self-aggregate efficiently removes proliferative and non-endocrine cells. Subsequent suspension culture yields SC-islets with strong glucose responsiveness *in vitro*. After transplantation into the anterior chamber of the eye of diabetic mice, SC-islets further mature and restore normal glycemic control. Single-cell analyses show that the SC-islets are free of non-endocrine cell populations before and after transplantation. This protocol enables production of highly functional SC-islets suitable for T1D cell therapy.

INTRODUCTION

Pancreatic islets regulate blood glucose through coordinated hormone secretion by insulin-producing β cells, glucagon-producing α cells, and somatostatin-producing δ cells. In type 1 diabetes (T1D), autoimmune destruction of β cells results in loss of glycemic control. Transplantation of cadaveric islets can restore insulin independence in patients with T1D (Shapiro et al., 2000; Brennan et al., 2016), but broader application is limited by donor scarcity and the requirement for chronic immunosuppression or immune-protective devices. Human pluripotent stem cells (hPSCs) can undergo unlimited self-renewal and can be engineered to reduce immune rejection (Sintov et al., 2022; Gerace et al., 2023; Hu et al., 2023), making them an attractive alternative cell source for transplantation therapies (Ramzy et al., 2021; Wang et al., 2024a; Reichman et al., 2025).

Differentiation protocols have advanced substantially and guide hPSCs through stages mimicking pancreatic development (Figure 1A), ultimately producing stem cell-derived pancreatic islets (SC-islets) (D'Amour et al., 2006; Kroon et al., 2008; Pagliuca et al., 2014; Rezania et al., 2014; Nostro et al., 2015; Velazco-Cruz et al., 2019; Veres et al., 2019; Hogrebe et al., 2020; Hogrebe et al., 2021; Balboa et al., 2022; Barsby et al., 2022). However, several key challenges persist. Differentiation beyond the stage (S) 4 pancreatic progenitor (PP) stage frequently yields heterogeneous cultures containing proliferative non-endocrine cells and immature endocrine cells (Sharon et al., 2019b; Veres et al., 2019; Rajaei et al., 2025), increasing the risk of cyst or tumor formation (Kroon et al., 2008; Kelly et al., 2011; Rezania et al., 2012; Aghazadeh et al., 2022; Lithovius et al., 2024). Although efficient induction of PP cells has been achieved in multiple hPSC lines (Nostro et al., 2015; Cogger et al., 2017; Aghazadeh et al., 2022; Balboa et al., 2022), further optimization is needed for the S5 endocrine progenitor (EP) stage and beyond. In addition, SC-islets commonly display reduced glucose-stimulated insulin secretion (GSIS) compared with human islets (Pagliuca et al., 2014; Rezania et al., 2014; Lyon et al., 2016; Cogger et al., 2017; Velazco-Cruz et al., 2019; Davis et al., 2020). Studies of pancreatic development indicate that EP cells delaminate from the epithelium and form 3D aggregates *in vivo* (Jeon et al., 2009; Gouzi et al., 2011; Nair and Hebrok, 2015; Sharon et al., 2019a), but most protocols do not fully recapitulate this process. Moreover, differentiation efficiencies vary across hPSC lines (Rezania et al., 2014).

Here, we report a refined differentiation strategy that consistently generates functional SC-islets across eight hPSC lines. Optimizing EP differentiation on 2D laminin (LN)-521, followed by spontaneous 3D aggregation, produces endocrine clusters devoid of non-endocrine cells

© 2026 The Authors.
Published by Elsevier Inc. on behalf of International Society for Stem Cell Research.
This is an open access article under the CC BY license (http://creativecommons.org/licenses/by/4.0/).

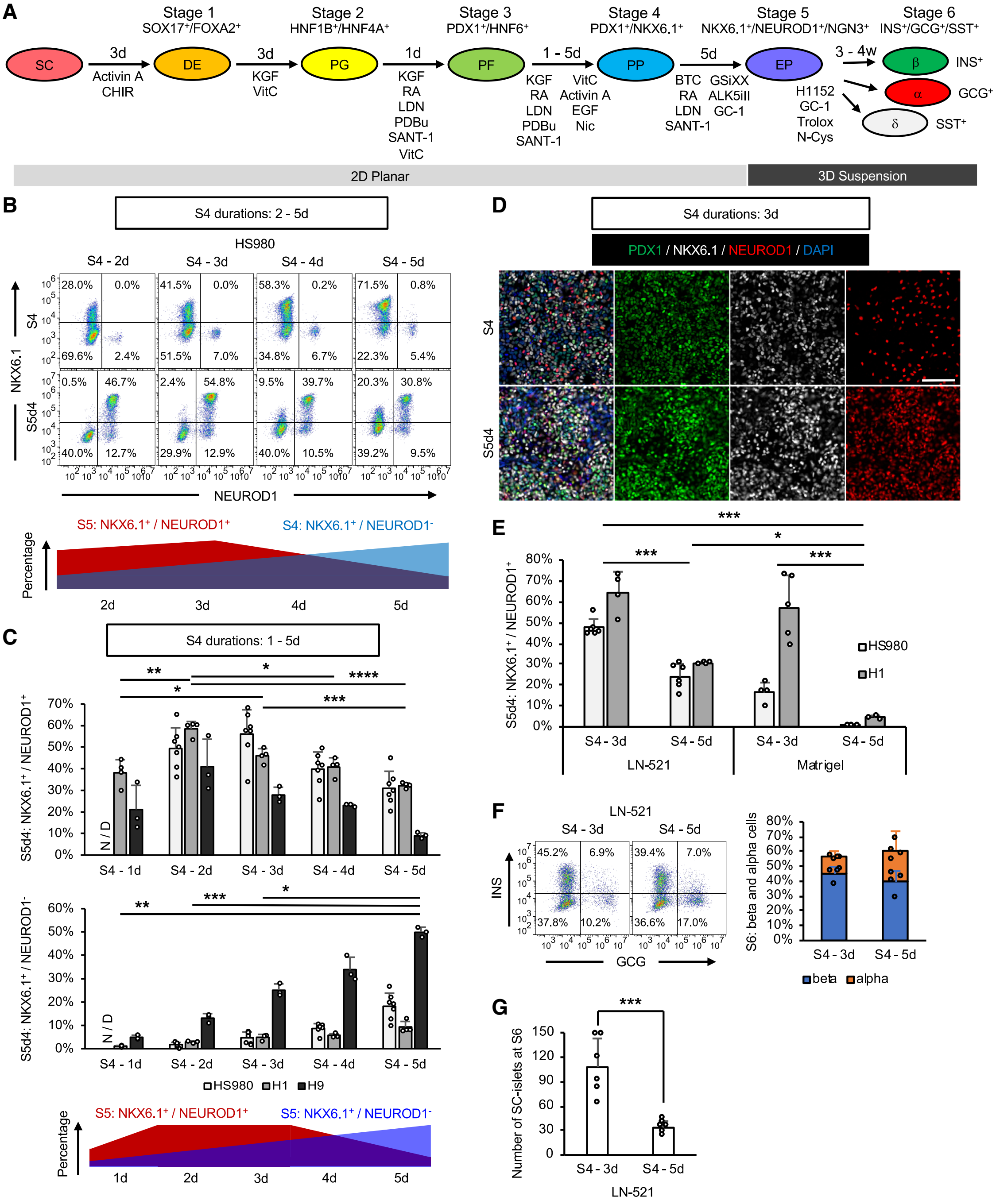

Figure 1. Short S4 duration enhances S5 EP differentiation

(A) Schematic of stages S1–S6 with durations, factors, and key markers.

(B) NKX6.1 and NEUROD1 expression in HS980 cells on LN-521 with S4 durations of 2–5 days, measured at the end of S4 or S5d4; representative dot plots shown.

(C) Percentages of NKX6.1$^+$/NEUROD1$^+$ and NKX6.1$^+$/NEUROD1$^-$ cells at S5d4 in HS980, H1, and H9 cells with S4 durations of 1–5 days. Data are means ± SD, n = 7 for HS980, 4 for H1, and 3 for H9; one-way ANOVA.

(D) Immunofluorescence of PDX1, NKX6.1, and NEUROD1 at S4 and S5d4; representative images, n = 3; scale bars, 100 μm.

(E) S5 EP differentiation with S4 durations of 3 or 5 days on LN-521 and Matrigel. Bar graphs show NKX6.1$^+$/NEUROD1$^+$ percentages in HS980 and H1 cells. Data are means ± SD, n = 3–6; one-way ANOVA.

(legend continued on next page)

that exhibit strong glucose responsiveness *in vitro* and restore glycemic control after transplantation into diabetic mice.

RESULTS

Short S4 duration enhances differentiation into S5 pancreatic EP cells

Current differentiation protocols efficiently generate PP cells, but the transition to EP remains suboptimal and often results in heterogeneous, proliferative cultures (Sharon et al., 2019b; Veres et al., 2019). To refine this step, we differentiated hPSCs toward S5 EP stage on 2D LN-521, a defined xeno-free substrate supporting hPSC derivation and expansion (Rodin et al., 2014; Main et al., 2020). The efficiency of differentiation into PDX1$^+$/NKX6.1$^+$ PP cells on LN-521 is comparable to that on Matrigel (Figures S1A and S1B). Because shortening S3 enhances PP induction at S4 (Nostro et al., 2015; Velazco-Cruz et al., 2019), we reduced S3 to 1 day and evaluated whether the duration of S4 affects downstream EP formation. Reducing S4 duration increased NKX6.1$^+$/NEUROD1$^+$ EP cells at day 4 of S5 (S5d4), despite lower NKX6.1 expression at S4 (Figure 1B), an effect consistent in two additional cell lines (Figure S1C). Systematic testing identified 2–3 days as optimal, whereas longer S4 generated more NKX6.1$^+$/NEUROD1$^-$ PP-like cells (Figure 1C). Immunocytochemistry (ICC) confirmed the efficient induction of PDX1$^+$/NKX6.1$^+$/NEUROD1$^+$ and PDX1$^+$/NKX6.1$^+$/NGN3$^+$ EP cells (Figures 1D and S1D). Both short S4 and LN-521 enhanced S5 EP differentiation efficiency, likely through additive effects (Figure 1E). EP cells from short (3-day) or long (5-day) S4 durations on LN-521 were aggregated into 3D at S5d4, generating SC-islets containing both mono-hormonal INS$^+$/GCG$^-$ β cells and GCG$^+$/INS$^-$ α cells at the end of S6 (Figure 1F). Short S4 cultures, however, yielded significantly more SC-islets (Figure 1G), indicating that efficient S5 EP differentiation under short S4 conditions is critical for SC-islet formation. Although short S4 enabled S5 EP differentiation on both LN-521 and Matrigel (Figure 1E), LN-521 yielded higher proportions of INS$^+$/GCG$^-$ β cells at S6 than Matrigel (Figure S1E). As LN-521, also available in GMP-compatible quality, supports hPSC derivation and expansion (Rodin et al., 2014; Main et al., 2020), all subsequent differentiation experiments were performed on LN-521 using 1 day of S3 and 3 days of S4 (hereafter referred to as the short differentiation protocol; see "methods").

3D aggregation at the S5 EP stage enriches endocrine cells and eliminates non-endocrine populations

To identify the optimal stage for transitioning to 3D culture, differentiating cells on LN-521 were dissociated at either the end of S4 or at S5d4, and allowed to aggregate spontaneously (Figure S2A).

Aggregation of S5 EP cells generated >10-fold more SC-islets at S6, and these aggregates contained higher proportions of INS$^+$/GCG$^-$ β cells and GCG$^+$/INS$^-$ α cells compared with aggregates derived from S4 PP cells (Figures S2B and S2C). We next examined whether S5 EP cells were selectively enriched by spontaneous aggregation at S5d4 (Figure 2A). One day after aggregation, EP clusters showed increased NKX6.1$^+$/NEUROD1$^+$ EP cells and a marked reduction in NEUROD1$^-$ non-endocrine and Ki-67$^+$ proliferative cells (Figures 2B and 2C). Replating dissociated EP cells on LN-521 retained a substantial fraction of these unwanted cells (Figures 2B and 2C), demonstrating that 3D aggregation is required for efficient enrichment of EP cells. ROCK inhibitor (H1152) improved aggregation efficiency and cell recovery (Figure S2E), without affecting EP identity (Figure S2D). Aggregation in either ULA or AggreWell formats yielded similar EP and final endocrine compositions (Figure S2F).

During S6 differentiation, INS$^+$/GCG$^-$ β cells remained ~50% of the culture while GCG$^+$/INS$^-$ α cells progressively increased and INS$^+$/GCG$^+$ polyhormonal cells declined to <5% (Figure 2D). By week 4 of S6 (S6w4), most β cells co-expressed NKX6.1, and NEUROD1$^-$ and Ki-67$^+$ cells were further reduced to 2.2 ± 1.32% and 0.88 ± 0.37%, respectively (Figure 2E). ICC confirmed mono-hormonal β and α cells, as well as SLC18A1$^+$ EC-like cells (Figure 2F). S5 EP cells could be cryopreserved and later differentiated into SC-islets with comparable endocrine composition and static GSIS (Figures S2G and S2H). Despite ~40%–50% cell loss during aggregation (Figure S2E), the protocol still produced a net expansion of endocrine cells by S6w4 (Figure 2G; Table S1). Taken together, these results show that S5 EP cells undergo efficient self-aggregation and differentiation into SC-islets, while proliferative and non-endocrine cells are selectively removed.

Functional SC-islets are generated across multiple hPSC lines

To address cell-line variability in SC-islet differentiation, we evaluated the protocol across eight hPSC lines, four embryonic (HS980, H1, H9, and KARO1) and four induced

(F and G) Differentiation to S6 SC-islets from dissociated S5 EP cells with 3- or 5-day S4 on LN-521. (F) INS and GCG expression at S6; representative dot plots and bar graphs show INS$^+$/GCG$^-$ β and GCG$^+$/INS$^-$ α cells. Data are means ± SD, n = 4. (G) SC-islet counts from 1 × 10^6 EP cells; data are means ± SD, n = 6; unpaired two-tailed t test. Statistical significance: *p < 0.05, **p < 0.01, ***p < 0.001, ****p < 0.0001. See also Figure S1.

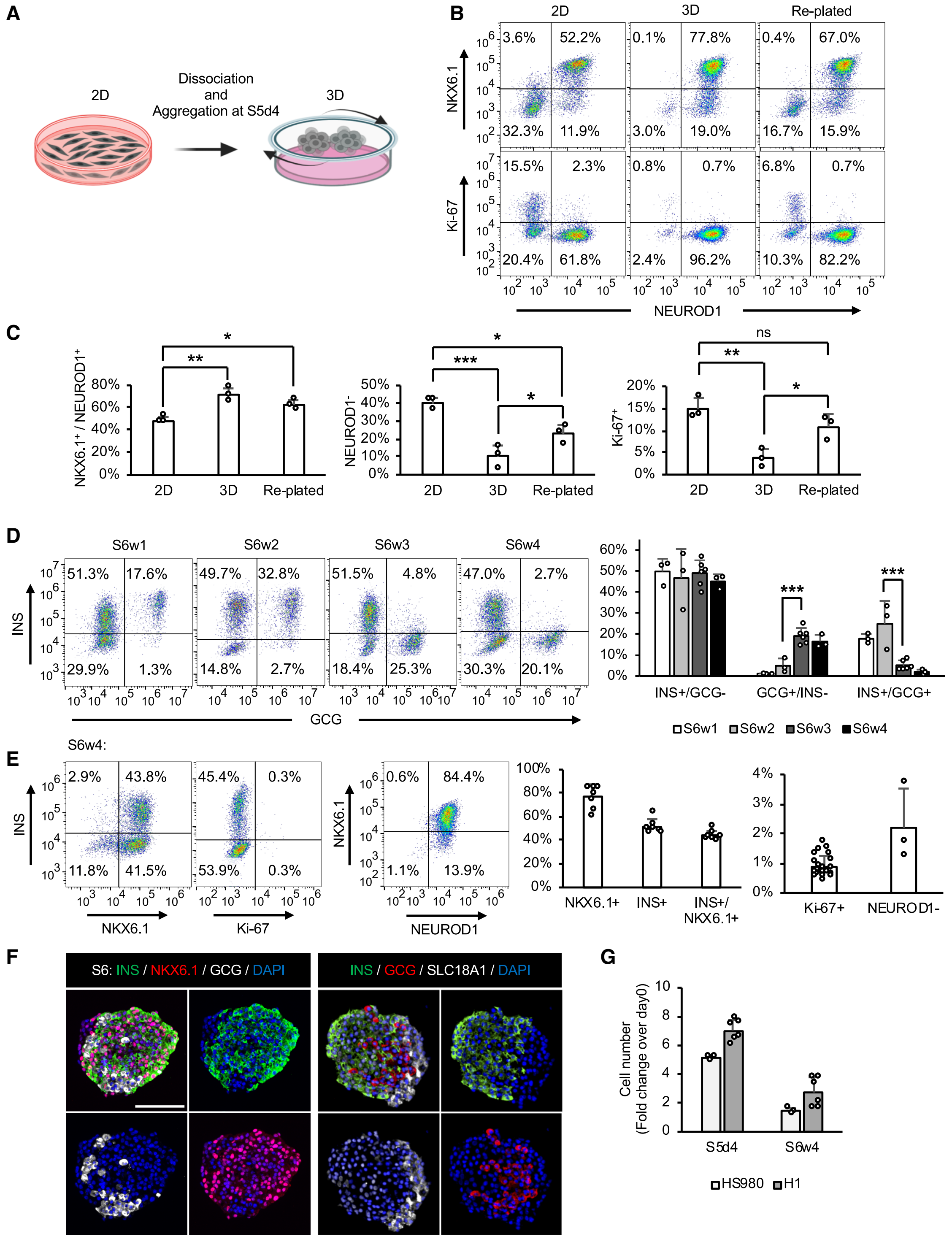

Figure 2. 3D aggregate formation at S5 EP stage effectively removes non-endocrine cells

(A) Schematic of 3D aggregate formation at S5d4. Cells differentiated on LN-521 were dissociated and maintained in suspension (3D) or re-plated on LN-521.

(B and C) NKX6.1, NEUROD1, and Ki-67 expression before (2D) and one day after dissociation (3D, Re-plated) by flow cytometry. (B) Representative dot plots; (C) bar graphs show NKX6.1$^+$/NEUROD1$^+$ EP cells, NEUROD1$^-$ non-endocrine cells, and Ki-67$^+$ proliferative cells; data are means ± SD, n = 3; one-way ANOVA.

(legend continued on next page)

pluripotent (C7, C9, C12, and C14), without excluding any line (Figure 3A). All lines generated SC-islets containing appropriate proportions of mono-hormonal INS^+/GCG^- β cells and GCG^+/INS^- α cells (Figure 3B). At the end of S6, SC-islets from all lines showed glucose-stimulated C-peptide secretion with a return to baseline after glucose withdrawal (Figure 3C), within the range reported for freshly isolated human islets (1.4- to 37.3-fold) (Lyon et al., 2016). SC-islets derived from HS980, H1, and H9 exhibited particularly strong glucose responses. Dynamic GSIS of HS980 SC-islets demonstrated a pronounced first-phase insulin release (16.1-fold), followed by sustained second-phase secretion (6.1-fold) (Figure 3D), closely matching human islet responses (15.0- and 6.7-fold) (Velazco-Cruz et al., 2019). To determine whether our protocol overcomes a previously described glycolytic bottleneck (Davis et al., 2020), c-peptide secretion was measured in response to glyceraldehyde. SC-islets responded strongly (Figure 3E), indicating restored glyceraldehyde metabolism, unlike prior SC-islet protocols (Davis et al., 2020; Balboa et al., 2022).

We further characterized SC-islets with ICC and transmission electron microscopy (TEM) (Figures 3F and 3G). Monohormonal $CPEP^+$ β, GCG^+ α, and SST^+ δ cells were confirmed (Figure 3F). β cells contained granules with dark and dense insulin crystalline cores surrounded by a light halo, while α cells displayed large dark vesicles, some with gray halo surrounding a dense core (Figure 3G), both resembling primary human islet ultrastructure (Pagliuca et al., 2014; Peterson et al., 2020; Balboa et al., 2022).

We compared our shortened LN-521-based approach with two published protocols (Velazco-Cruz et al., 2019; Balboa et al., 2022). The 3D long protocol of Velazco-Cruz et al. (5-day S4) (Velazco-Cruz et al., 2019) generated S5 $NKX6.1^+$/$NEUROD1^+$ EP cells primarily from H1 cells (Figure S3B), whereas our protocol produced EP cells across all eight hPSC lines (Figure S3A), consistent with the combined requirement for both short S4 duration and LN-521 (Figure 1E). Further differentiation of H1 cells using the Velazco-Cruz protocol yielded INS^+/GCG^- β cells, few GCG^+/INS^- α cells, abundant INS^+/GCG^+ polyhormonal cells, and minimal static GSIS (1.3-fold; Figures S3C and S3D). The Balboa et al. protocol (Balboa et al., 2022), which maintains cells on 2D through S4 before aggregation, generated more α cells but fewer β cells and exhibited glucose responsiveness (2.75-fold; Figures S3C and S3D). In contrast, our protocol consistently generated mature SC-islets with higher fraction of INS^+/GCG^- β cells and robust glucose-stimulated secretion (9.5-fold).

Collectively, these results demonstrate that the combination of shortened S4, LN-521-based 2D culture, and spontaneous S5 aggregation enables robust and scalable generation of functional SC-islets across diverse hPSC lines, as further supported by comparison to previous protocols (Table S1) (Velazco-Cruz et al., 2019; Augsornworawat et al., 2020; Hogrebe et al. 2020, 2021; Balboa et al., 2022; Barsby et al., 2022; Maxwell et al., 2022; Lithovius et al., 2024; Rajaei et al., 2025).

Single-cell transcriptome profiling of SC-islets identifies key endocrine but not non-endocrine cell types

Single-cell RNA sequencing (scRNA-seq) of H1 SC-islets at day 43 revealed three major endocrine populations, β, α, and EC-like cells, all expressing chromogranin A (*CHGA*) (Figures 4A, S4A, and S4B). β cells accounted for 64% of all cells and expressed *INS*, *PDX1*, *NKX6.1*, and *ISL1* (Figures 4B, S4B, and S4C). Two β cell sub-clusters were detected: an early population marked by *HADH* (Balboa et al., 2022) and *ASCL1* (Veres et al., 2019), and a mature population expressing *IAPP*, *BACE2* (Díaz-Catalán et al., 2021), *PCDH7* (Yoon et al., 2022), and *CACNA2D1* (Tuluc et al., 2021), key regulators of β cell functional maturation and insulin secretion. Both populations showed high *INS* and low *GCG* expression. α cells (17%) expressed *GCG*, *ARX*, and *IRX2* and, unlike β cells, showed high *GCG* and low *INS* expression (Figures 4A, 4B, S4B, and S4C), consistent with their expected hormone identity.

Differential expression analysis revealed enrichment of *NEUROD1* and *PAX6* in mature β cells, both master regulators of β cell identity and insulin secretion (D'Amour et al., 2006; Gosmain et al., 2012; Mastracci et al., 2013; Bohuslavova et al., 2021), alongside maturation-associated genes such as *BACE2*, *PCDH7*, and *CACNA2D1* (Figure 4C). Pathway analysis highlighted KEGG pathways linked to β cell maturation, including protein processing in the ER, AMPK signaling, insulin secretion, and PPAR signaling (Figure 4D), all central to β cell metabolic and secretory function (Svendsen et al., 2018; Zhu et al., 2019; Entezari et al., 2022). GSEA further showed downregulation of Hippo and TGF-β signaling in mature β cells (Figure 4E), consistent with their roles for β cell

(D and E) S6 differentiation from S5d4 in 3D suspension. (D) INS and GCG expression during S6w1–4; representative dot plots and bar graphs; data are means ± SD, $n = 3–6$; one-way ANOVA. (E) NKX6.1, INS, NEUROD1, and Ki-67 expression at S6w4; representative plots and bar graphs; data are means ± SD, $n = 7$ (INS, NKX6.1), 21 (Ki-67), 3 (NEUROD1).
(F) Immunofluorescence of INS, GCG, NKX6.1 (left) and SLC18A1 (right) at S6w4; representative images, $n = 3$; scale bars, 100 μm.
(G) Cell numbers at S5d4 and S6w4 as fold change over day 0. Data are means ± SD, $n = 3–6$. Statistical significance: ns, not significant; *$p < 0.05$, **$p < 0.01$, ***$p < 0.001$. See also Figure S2.

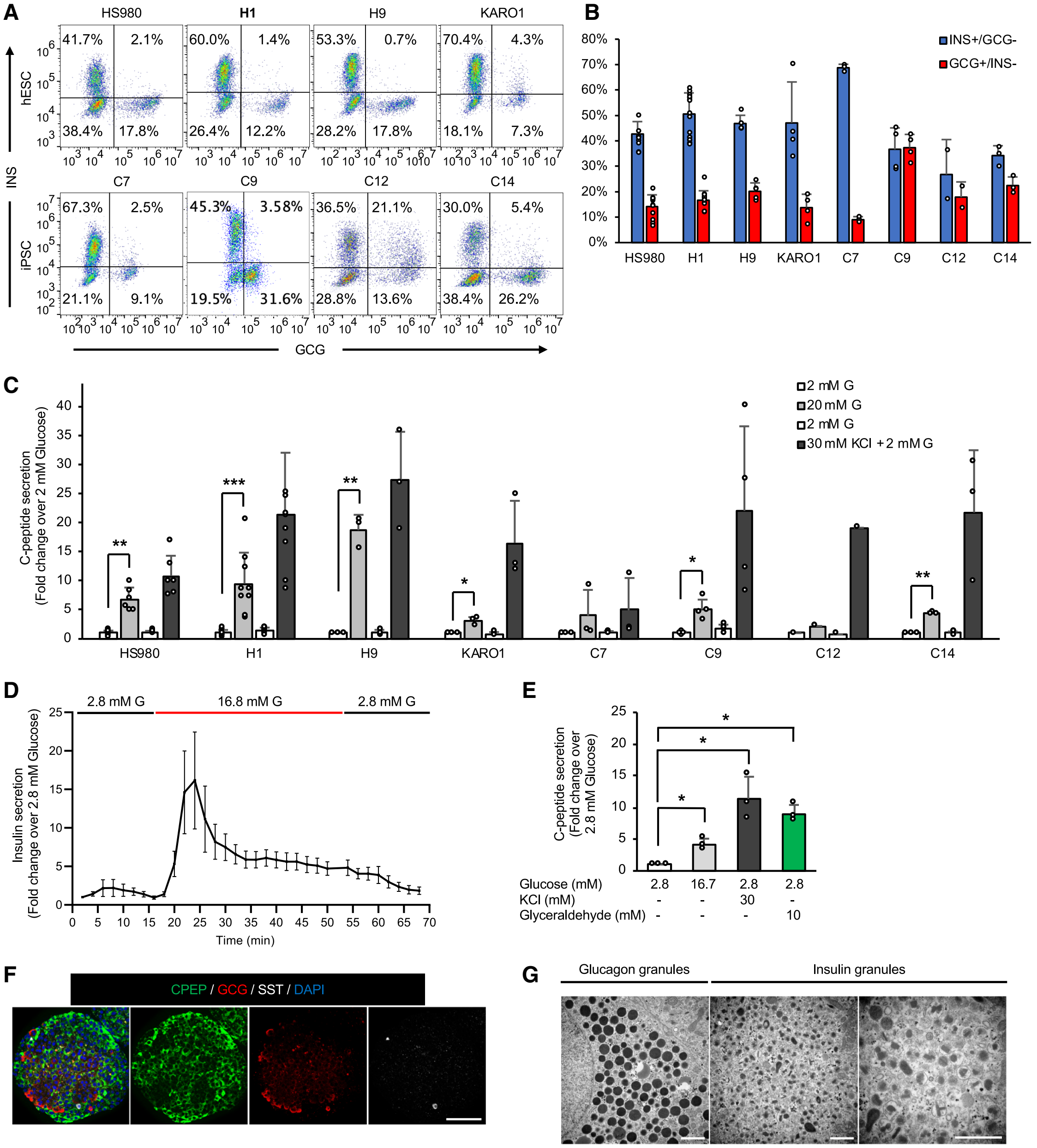

Figure 3. Functional SC-islets generated from multiple hPSC lines

(A and B) Eight hPSC lines differentiated using the short differentiation protocol (Wu et al.). INS and GCG assessed by flow cytometry at S6. (A) Representative dot plots and (B) bar graphs are shown. Data are means ± SD, n = 12 (HS980), 14 (H1), 5 (H9), 4 (KARO1, C9), 3 (C7, C14), 2 (C12).

(C) Static GSIS showing fold change in c-peptide over 2 mM glucose; data are means ± SD, n = 6 (HS980), 9 (H1), 3 (H9, KARO1, C7, C14), 4 (C9), 1 (C12); paired two-tailed t tests.

(D) Dynamic GSIS from HS980 SC-islets; fold change over 2.8 mM glucose; data are means ± SEM, n = 4.

(E) Glyceraldehyde-stimulated insulin secretion; fold change over 2.8 mM glucose; data are means ± SD, n = 3; paired two-tailed t test.

(F) Immunofluorescence of CPEP, GCG, and SST in H1 SC-islets; representative images, n = 3; scale bars, 100 μm.

(G) TEM showing insulin and glucagon granules in H1 SC-islets; scale bars, 1 μm. Statistical significance: *p < 0.05, **p < 0.01, ***p < 0.001, ****p < 0.0001. See also Figure S3.

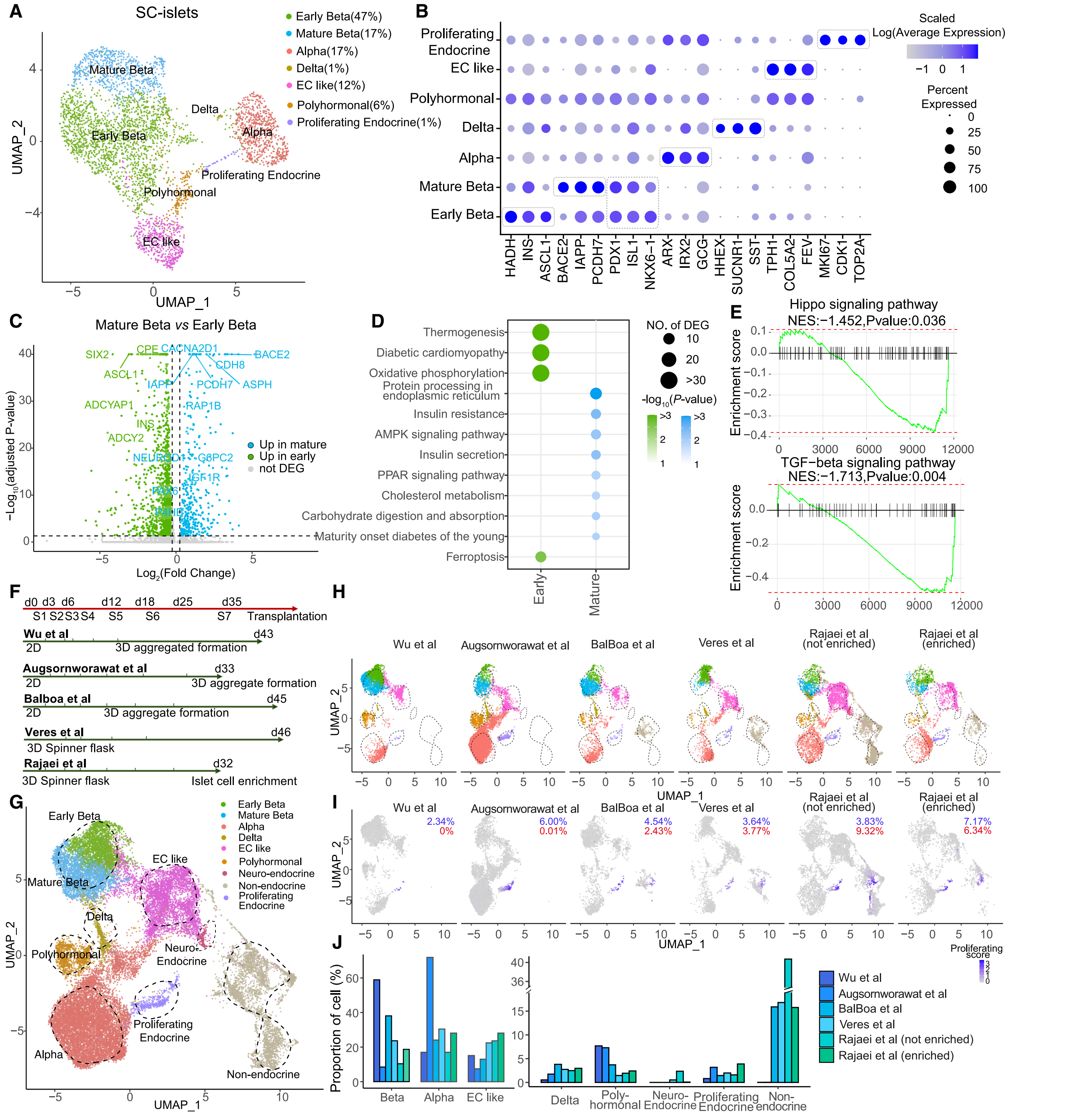

Figure 4. Single-cell transcriptomics define endocrine cell types in SC-islets

(A) UMAP of day 43 SC-islets (H1) highlighting major endocrine populations.

(B) Dot plot of marker genes used for cell-type annotation.

(C) Volcano plot showing fold-change in expression and Bonferroni-adjusted two-sided p values for DEGs between mature and early β cells in day 43 H1 SC-islets.

(D) KEGG enrichment dot plot for DEGs, showing terms with one-sided $p < 0.05$. Dot size reflects DEG counts in each term and color indicates p value.

(legend continued on next page)

differentiation (Mamidi et al., 2018; Rosado-Olivieri et al., 2019; Velazco-Cruz et al., 2019). Together, these data indicate a transition toward enhanced insulin secretory capacity during β cell maturation.

EC-like cells comprised 12% of the SC-islets and expressed *TPH1*, *FEV*, and *COL5A2* (Figures 4A and 4B). Recent studies show that EC-like and SC-β cells display gradient identities (Augsornworawat et al., 2023), and resemble transient 5-HT-producing pre-β cells in fetal pancreas development (Zhu et al., 2023). Three minor populations were detected, namely polyhormonal cells (6%), co-expressing *INS* and *GCG*, δ cells (1%), expressing *SST*, *HHEX*, and *SUCNR1*, and proliferating endocrine cells (1%), expressing proliferation markers *MKI67*, *TOP2A*, and *CDK1* (Figures 4A, 4B, S4B, and S4C). γ and ε cells were rare, showing only sparse marker expression (Figure S4B). Non-endocrine cells were not detected, aside from a few cells co-expressing *CPA1* and *CHGA* but not *TOP2A*, and no mesenchymal, endothelial, or neuronal cells were observed (Figure S4B).

We next compared our SC-islets (Wu et al.) with four published scRNA-seq datasets (Figure 4F). Augsornworawat et al. differentiated HUES8 cells on 2D until stage 6 before 3D aggregation and performed scRNA-seq on day 33 (Augsornworawat et al., 2020). Balboa et al. differentiated H1 cells to S4 PP on 2D, then formed 3D aggregates in microwells and profiled cells on day 45 (S7w3) (Balboa et al., 2022). Veres et al. differentiated HUES8 cells in 3D and analyzed cells on day 46 (S6w4) (Veres et al., 2019). Rajaei et al. reported a GMP-compliant 3D suspension protocol with density-based purification (Rajaei et al., 2025). Integration of all datasets, using the original annotations, revealed distinct cell-type composition across protocols (Figures 4G, 4H, and S4D). Our dataset showed the highest fraction of β cells and the fewest δ cells (Figures 4H, 4J, and S4D). Augsornworawat et al. had the highest α-cell fraction (71%), while all datasets displayed similar levels of EC-like cells (8%–25%) (Figure 4J). Non-endocrine cells were present in Balboa, Veres, and Rajaei et al. datasets (16%–41%) (Figures 4H, 4J, and S4D). A subset of these non-endocrine cells expressed proliferation markers (2.4%–9.3%) (Figure 4I, red), as described by Veres et al., (2019). Previous reports linked such cells to cyst and tumor risk (Kroon et al., 2008; Kelly et al., 2011; Rezania et al., 2012; Aghazadeh et al., 2022; Lithovius et al., 2024). Although all datasets contained endocrine cells with proliferative signatures (*MKI67*, *CDK1*, *TOP2A*, *CCNB2*, *CCNA2*, and *PBK*) (Figure 4I, blue), our SC-islets showed the lowest level (Figure 4I, blue).

Intraocular transplantation of SC-islets reverses preexisting diabetes in mice

The anterior chamber of the eye (ACE) provides a transparent and accessible site for noninvasive monitoring of engrafted SC-islets through the cornea, while transplantation into this compartment is straightforward and minimally invasive (Speier et al. 2008a, 2008b; Berggren et al., 2024). To evaluate *in vivo* function and maturation, we transplanted SC-islets into the ACE of streptozotocin (STZ)-induced diabetic mice (Figure 5A). Non-fasting blood glucose and plasma human c-peptide levels in 11–12 diabetic mice were monitored for 6 months (Figures 5B and 5C). SC-islet transplantation reversed hyperglycemia by 3 months, and by 5–6 months blood glucose levels fell slightly below pre-STZ baselines (Figure 5B), consistent with species-specific glycemic set points reported previously (Rodriguez-Diaz et al., 2018). Plasma human c-peptide was detectable at 1 month, increased by 3 months, and remained stable, correlating inversely with glucose levels; no mouse c-peptide was detected, confirming glycemic control by SC-β cells (Figure 5C).

Intraperitoneal glucose tolerance tests (IPGTT) at 3, 4, and 6 months post-transplantation showed improved glucose handling over time (Figures 5D–5F). At 3 months, glucose peaked at 30 min and declined by 60 min, whereas at 4 and 6 months it began falling by 30 min and nearly returned to baseline by 120 min (Figure 5D). Plasma human C-peptide increased after glucose injection, with larger responses and a clearer peak-and-decline pattern at 4 and 6 months (Figure 5E). Together, these data indicate enhanced GSIS and continued functional maturation of SC-islets *in vivo*.

At 6 months post-transplantation, eyes were enucleated for cryosectioning. Engrafted SC-islets contained monohormonal INS^+ β, GCG^+ α, and SST^+ δ cells (Figure 5G).

(E) GSEA enrichment curves comparing mature and early β cells.
(F) Schematic comparison of timing and 2D/3D formats in published protocols (Augsornworawat, Balboa, Veres, Rajaei) versus Wu et al.
(G) Integrated UMAP embedding of datasets from different protocols showing β, α, δ, EC-like, polyhormonal, neuro-endocrine, non-endocrine, and proliferating endocrine clusters.
(H) UMAP embedding segregated by protocol.
(I) UMAP of the proliferation signature across protocols, showing the percentages of endocrine (blue) and non-endocrine (red) cells with positive proliferation module scores.
(J) Cell-type composition across protocols, with major endocrine subsets (β, α, EC-like) displayed on the right and other populations on the left side. See also Figure S4.

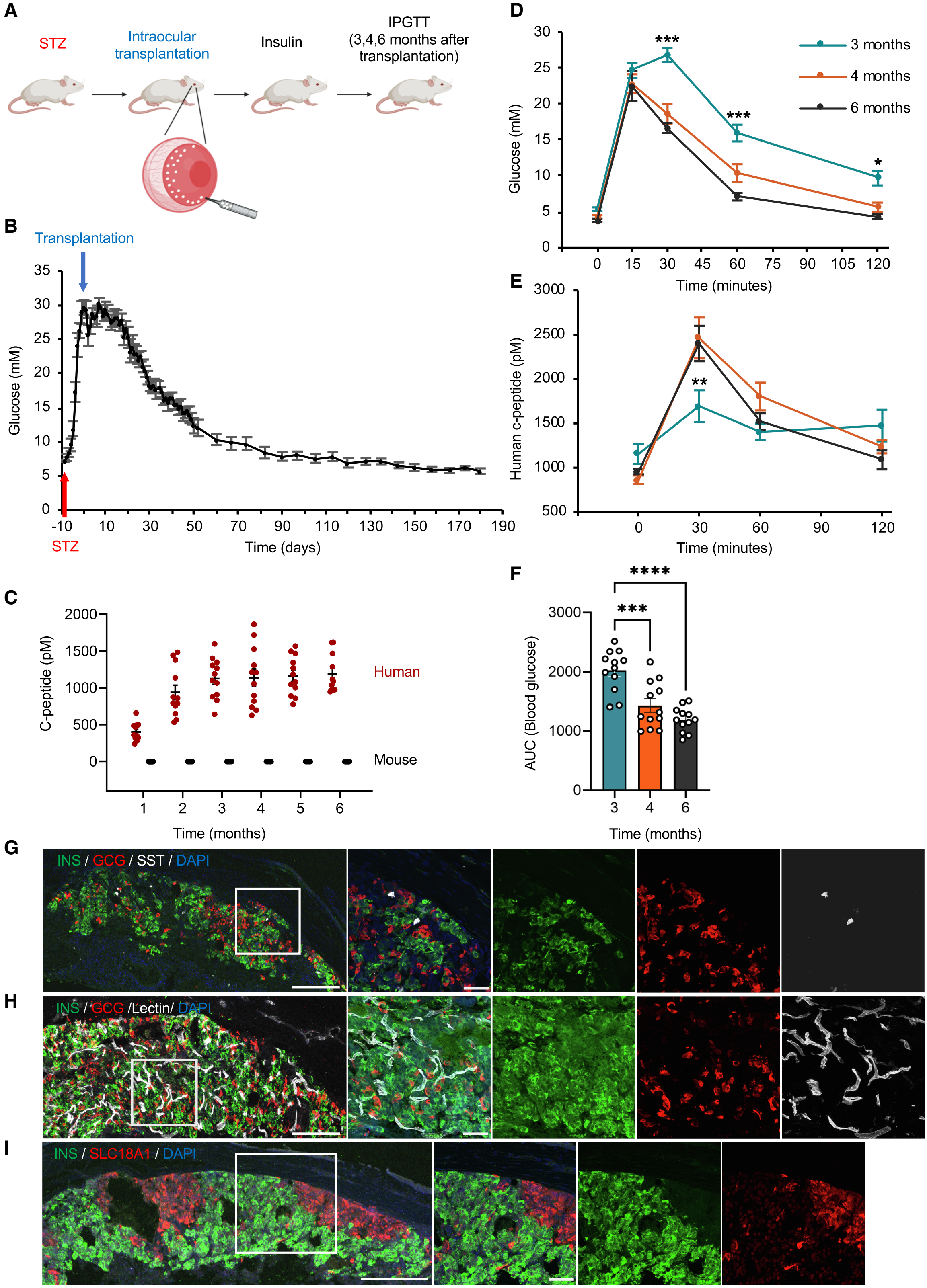

(legend on next page)

Lectin staining revealed blood vessels within the grafts, confirming vascularization (Figure 5H). SLC18A1$^+$ EC-like cells were also detected in discrete regions of the grafts (Figure 5I).

SC-islets undergo maturation *in vivo*

To assess *in vivo* maturation, HS980 SC-islets were analyzed by scRNA-seq 20 weeks after transplantation. The same endocrine populations seen pre-transplantation were identified; α, early β, mature β, δ, EC-like, polyhormonal, and proliferating endocrine cells, all expressing expected markers (Figures 6A–6C and S5A–S5H). Mature β cells increased from 22% to 54%, while early β cells decreased from 28% to 20%, yielding a total β-cell mass of 74% (Figure 6C). α and EC-like cells declined, whereas polyhormonal cells expanded (Figure 6C). A minor population of non-proliferating pancreatic stellate cells expressing the ECM gene *DCN*, but not *TOP2A*, emerged post-transplantation (Figures 6A–6C and S5H), indicating a low risk of islet fibrosis or related damage (Wang et al., 2024b). Proliferating β cells (1%) co-expressing *INS* and *TOP2A* were also detected (Figures 6A–6C, S5G, and S5H), consistent with the low β cell proliferation rate reported previously (Kassem et al., 2000). Few cells expressed *CPA1*, but all co-expressed *CHGA*, indicating the absence of true exocrine or other non-endocrine cells (Figures S5F and S5H). No EP, mesenchymal, endothelial, or neuronal cells were observed (Figures S5F and S5H).

RNA velocity revealed trajectories from early to mature β cells and from α to polyhormonal cells (Figure S6A), consistent with the increased mature β and polyhormonal fractions after transplantation (Figure 6C). To examine *in vivo* β cell maturation (Figure 6D), we compared gene expression in early and mature β cells before and after transplantation (Figures 6E and S6B–S6D). Most differentially expressed genes were upregulated within the same β cell subpopulation post-transplantation (Figure S6C), including genes associated with mature β cell identity and insulin secretion (Figures 6E and S5E–S5H), indicating *in vivo* maturation consistent with previous studies (Augsornworawat et al., 2020; Balboa et al., 2022). Hippo and TGF-β signaling gene sets were downregulated in mature β cells before and after transplantation (Figures S6B and S6D), supporting their role in functional β cell differentiation (Mamidi et al., 2018; Rosado-Olivieri et al., 2019; Velazco-Cruz et al., 2019).

DISCUSSION

Cell therapy for T1D requires efficient *in vitro* generation of transplantable SC-islets. Previous protocols exhibit variable efficiency across hPSC lines and often produce SC-islets with immature function and non-endocrine contaminants. Here, we present a new protocol for generating functional SC-islets from multiple hPSC lines, featuring two key improvements: (1) optimization of the S4 PP duration on 2D LN-521 to enhance S5 EP differentiation and (2) spontaneous 3D aggregation of single S5 EP cells, which removes proliferative and non-EP cells and yields SC-islets with greatly improved endocrine purity.

During stepwise pancreatic differentiation, high efficiency at intermediate stages is essential to prevent heterogeneous final populations. A recent study showed that extended expansion enhances early endodermal progenitor differentiation (Wong et al., 2023). Efficient generation of S4 NKX6.1$^+$ PP cells is particularly important, as endocrine induction before NKX6.1 produces polyhormonal rather than functional β cells (Kelly et al., 2011; Sharon et al., 2019b; Veres et al., 2019; Hogrebe et al., 2020; Peterson et al., 2020). Although earlier protocols used a long (5-day) S4 stage to enhance PP formation (Pagliuca et al., 2014; Cogger et al., 2017; Aghazadeh et al., 2022), we found that prolonged S4 duration impairs progression to S5 EP cells (Figure 1B). Shortening S4 to 2–3 days markedly improves S5 EP differentiation (Figure 1C), suggesting that S4 PP cells possess a limited temporal window of endocrine competence. Prolonged S4 culture may therefore reduce the fraction of cells able to initiate the endocrine differentiation program, resulting in fewer S5 EP cells.

To generate functional SC-islets, 2D progenitor cells must be organized into 3D aggregates. Previous methods using multipotent S4 PP cells often required enforced aggregation

Figure 5. Intraocular SC-islet transplantation reverses preexisting diabetes in mice
(A) Experimental design of transplantation and analyses.
(B) Non-fasting blood glucose levels (mM) before and after transplantation.
(C) Human and mouse plasma c-peptide levels (pM) at indicated time points after transplantation. Data are means ± SEM, n = 11–12 (human), 5–9 (mouse).
(D–F) IPGTT at 3, 4, and 6 months post-transplantation. (D) Blood glucose and (E) human plasma c-peptide were measured at different time points following glucose injection, at 3, 4, and 6 months post-transplantation. Data are means ± SEM, n = 11–12; two-way ANOVA.
(F) Quantification of glucose values as area under the curve (AUC). Data are means ± SEM, n = 11–12; one-way ANOVA.
(G–I) Representative immunohistochemistry of eye sections with SC-islet grafts at 6 months (G) INS, GCG, SST, (H) vessels visualized with lectin, and (I) INS and SLC18A1; representative images, n = 3; scale bars: long, 200 μm; short, 50 μm. Statistical significance: *$p < 0.05$, **$p < 0.01$, ***$p < 0.001$, ****$p < 0.0001$.

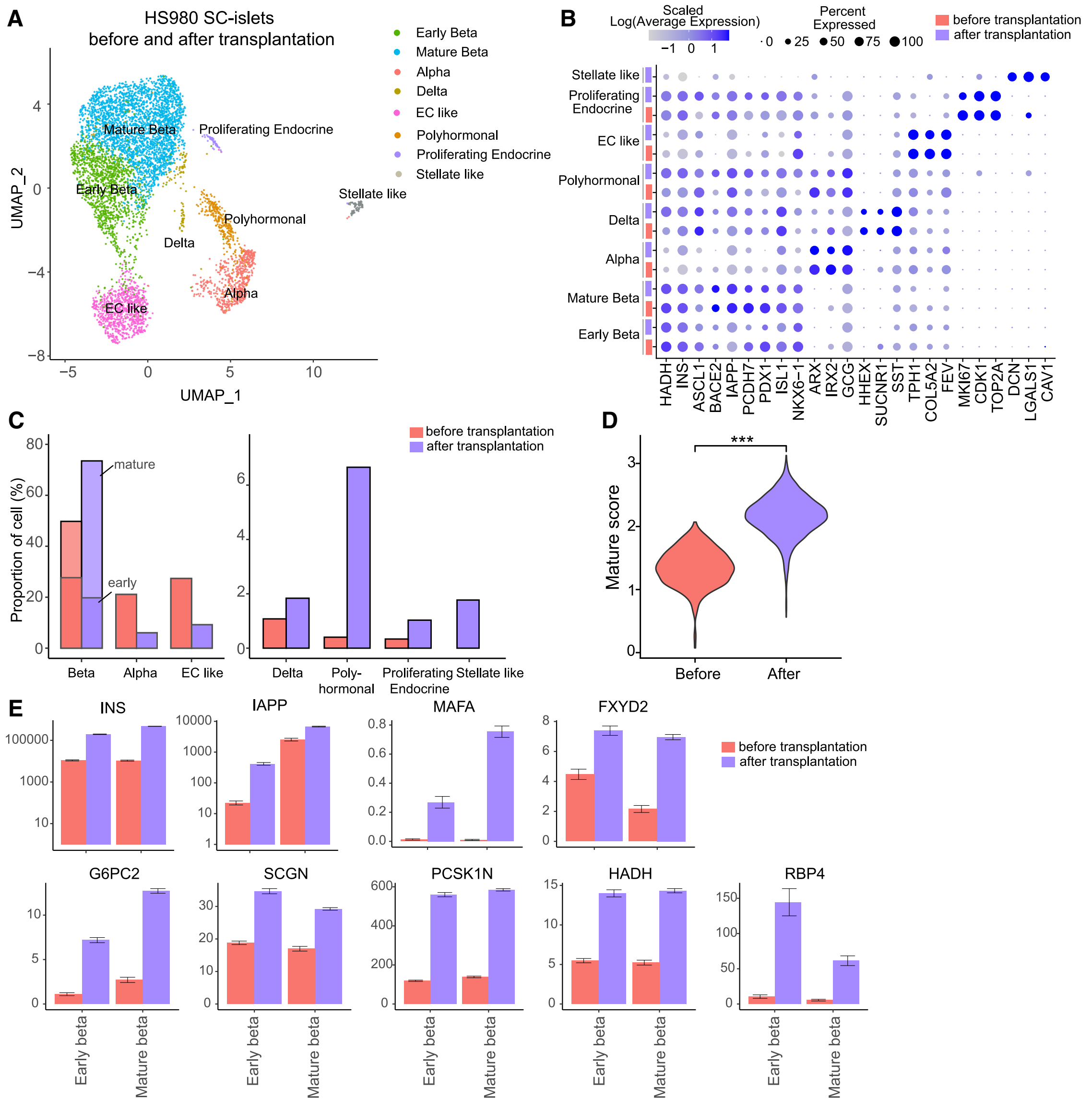

Figure 6. SC-islets undergo maturation *in vivo*

(A) UMAP projection of SC-islets from HS980 cells before and 20 weeks after transplantation, showing major cell populations.

(B) Dot plot showing the average expression levels of markers used for cell-type annotation; log-transformed values scaled separately for pre- and post-transplantation samples.

(C) Cell-type proportions before and after transplantation.

(D) Maturation signature of β cells and expression of genes involved in insulin secretion pre- versus post-transplantation; Wilcoxon test, $***p < 0.001$.

(E) Expression of selected DEGs in early and mature beta cells across pre- and post-transplantation states. See also Figures S5 and S6.

(Balboa et al., 2022; Barsby et al., 2022), yielding heterogeneous populations with proliferative non-endocrine cells (Figure 4I). In contrast, aggregation at S5 EP or later at S6, as in our protocol and in Augsornworawat et al., (2020), substantially reduced non-endocrine contaminants (Figures 4H–4J). We reasoned that S5 NEUROD1$^+$ EP cells could spontaneously form 3D islet-like aggregates, mirroring NeuroD1$^+$ cluster formation during mouse pancreas development (Gouzi et al., 2011; Sharon et al., 2019a). Indeed, S5 NEUROD1$^+$ EP cells formed 3D aggregates that excluded most NEUROD1$^-$ non-endocrine and Ki-67$^+$ proliferative cells (Figures 2A–2C). Aggregation was enhanced by low-dose ROCK inhibition (Figure S2E), reflecting the limited survival of non-adherent single EP cells

and consistent with EP cells migrating cohesively into NeuroD1$^+$ clusters, as described by Sharon et al. (2019a).

Our protocol generated glucose-responsive SC-islets from all eight hPSC lines tested (Figures 3A–3D), demonstrating potential for autologous applications. scRNA-seq confirmed high endocrine purity, with only 1% proliferative endocrine cells and no non-endocrine contaminants, though EC-like cells remained (Figures 2F, 4A, and 4F–4J). Transplantation into diabetic mice reversed hyperglycemia and restored glucose homeostasis (Figure 5). Post-transplant analysis showed *in vivo* maturation of SC-β cells and a reduced fraction of EC-like cells (Figures 6C–6E). No graft growth or cysts were observed after 6 months. While a minor population of pancreatic stellate cells was detected, these cells were largely non-proliferative, suggesting a low risk of islet fibrosis or related damage.

Several limitations should be noted. The molecular basis of the enhanced endocrine differentiation following S4 shortening remains unclear. β cell function was demonstrated by GSIS assays and reversal of hyperglycemia after transplantation. Due to considerable variabilities between primary human islet preparations, a direct comparison was not meaningful and therefore not performed. Further studies including electrophysiology, Ca^{2+} imaging, responses to other secretagogues, and assessment of glucagon secretion from α cells would in addition define endocrine maturity. Finally, although transplantation restored glycemic control without cyst formation for up to 6 months, longer-term studies are needed to assess graft stability and potential stromal remodeling.

Transplantation of autologous iPSC-derived islets has achieved sustained insulin independence (Wang et al., 2024a), and sufficiently pure, mature autologous SC-islets are expected to reduce immune rejection. Our efficient differentiation protocol represents a key step toward autologous cell therapy, though further work is required to realize this goal.

METHODS

hPSC culture and differentiation

Human ESC lines HS980 (KIe033-A), KARO1 (KIe034-A), H1 (WAe001-A), and H9 (WAe009-A), and iPSC lines C7, C9, C12, and C14, were cultured under xeno-free conditions in NutriStem hPSC XF Medium (Biological Industries, 05-100-1A) on tissue-culture plates coated with 10 μg/mL laminin (LN)-521 (BioLamina, LN521) (Rodin et al., 2014; Main et al., 2020; Plaza Reyes et al., 2020). Cells were maintained at 37°C, 5% CO_2, and 5% O_2, routinely confirmed mycoplasma-free, and validated for pluripotency marker expression by flow cytometry. Cells were passaged every 3–5 days using TrypLE Select (Thermo Fisher, A1285901) and re-plated at 15,000–24,000 cells/cm^2. Full culture details are provided in supplemental methods.

Upon reaching 90%–100% confluence, hPSCs were differentiated into pancreatic islet cells at 37°C, 5% CO_2, and 20% O_2, progressing through six stages (S1–S6) using the following factors:

S1 Definitive endoderm (3 days): 100 ng/mL Activin A (R&D, 338-AC) and 5 μM CHIR99021 (Tocris, 4423) for the first 24 h, followed by 100 ng/mL Activin A for 2 additional days.

S2 Primitive gut tube (3 days): 50 ng/mL KGF (R&D, 251-KG).

S3 Posterior foregut (1 day): 50 ng/mL KGF, 2 μM retinoic acid (Sigma, R2625), 0.25 μM SANT-1 (Sigma, S4572), 0.5 μM PDBu (Tocris, 4153), and 200 nM LDN193189 (Tocris, 6053).

S4 PP (2–3 days): 50 ng/mL KGF, 100 ng/mL EGF (R&D, 236-EG), 5 ng/mL Activin A, 10 mM nicotinamide (Sigma, N0636), 100 nM retinoic acid, 0.25 μM SANT-1, 0.5 μM PDBu, and 200 nM LDN193189.

S5 EP (5 days): 20 ng/mL Betacellulin (R&D, 261-CE), 100 nM retinoic acid, 0.25 μM SANT-1, 100 nM GSI-XX (Sigma, 565789), 10 μM ALK5 inhibitor II (Cayman Chemical, 14794), 1 μM GC-1 (Tocris, 4554), and 100 nM LDN193189.

On day 4, cells were dissociated with Accutase, resuspended at 1.0–1.5 × 10^6 cells/mL in ultra-low attachment plates (Corning, 3471) with 10 μM ROCK inhibitor H1152 (Tocris, 2414), and cultured on an orbital shaker (Infors HT Celltron) at 95 rpm to form islet-like aggregates.

S6 Pancreatic islets (3–4 weeks): 10 μM H1152, 1 μM GC-1, 1 mM N-acetyl-L-cysteine (Sigma, A9165), and 10 μM Trolox (Merck Millipore, 648471). Aggregates maintained on orbital shaker (95 rpm).

Media were changed daily from S1 to S5 and every 2–3 days during S6. Full daily media compositions, factor concentrations, and details of short versus long differentiation protocols are provided in supplemental methods.

Flow cytometry

Cells were dissociated with Accutase, washed, and resuspended at 1 × 10^6 cells/mL in PBS. Live/dead staining was performed for 30 min at 4°C (Thermo Fisher, L34963 and L34965). After two PBS washes, cells were fixed in Cytofix/Cytoperm buffer (BD, 554722) for 20 min at 4°C, washed, and incubated with conjugated antibodies in 1× Perm/Wash buffer (BD, 554723) for 30 min at 4°C. Following two additional washes, cells were resuspended in FACS buffer (PBS + 2% FBS + 1 mM EDTA) and analyzed on a Beckman Coulter CytoFLEX S flow cytometer. Data

were processed using FlowJo v10.8.1. Antibodies are listed in Table S2.

Immunofluorescence

Cells were fixed in 4% paraformaldehyde for 20 min at room temperature (RT) and washed in PBS. Samples were blocked in 5% normal donkey serum (Merck Millipore; S30-100 mL) with 0.3% Triton X-100 (Sigma, T9284) for 1 h at RT, then incubated overnight at 4°C with primary antibodies diluted in PBS containing 0.1% Triton X-100 and 5% normal donkey serum. After washing, secondary antibodies were applied for 1 h at RT. For optical clearing, SC-islets were incubated in FocusClear (CelExplorer, FC-101) overnight at 4°C and mounted in MountClear (CelExplorer, MC-301). Imaging was performed using a Nikon ECLIPSE Ti2 spinning-disk confocal microscope.

Mouse eyes containing SC-islets were excised 6 months post-transplant, fixed in 10% formalin overnight at 4°C, washed in PBS, transferred sequentially to 10%, 20%, and 30% sucrose, embedded in optimal cutting temperature compound (OCT, Sakura Finetek, 4583), and frozen at −80°C. For vessel visualization, some mice before sacrifice received tail-vein injection of 100 μL Lycopersicon Esculentum Lectin, DyLight 649 (1 mg/mL, Thermo Fisher, L32472). Cryo-sections (16 μm) were permeabilized with 0.1% Triton X-100 for 15 min, blocked with 10% FBS for 2 h at RT, and stained with primary and secondary antibodies in buffer containing 1% FBS and 0.01% Triton X-100. Sections were mounted in ProLong Gold antifade reagent with DAPI (Thermo Fisher, P36931) and imaged on a Leica TCS SP8 X confocal microscope. Images were processed using Fiji.

Primary antibodies are listed in Table S3; secondary antibodies were conjugated to Alexa Fluor 488, 546, 633, and 647 (Thermo Fisher).

Static *in vitro* GSIS

SC-islets (20–30 per assay; S6w4) were preconditioned overnight in S6 medium without ITS-X (5 mM glucose). Islets were washed and pre-incubated for 2 h in Krebs buffer containing 2 mM glucose, then sequentially incubated for 30 min in Krebs buffer containing 2 mM glucose, 20 mM glucose, 2 mM glucose, and 2 mM glucose plus 30 mM KCl, with washes between steps. Supernatants were collected for human C-peptide quantification by ELISA (R&D, DICP00) and normalized to total cell number after Accutase dissociation. Detailed buffer composition and handling steps are provided in supplemental methods.

TEM

SC-islets at the end of S6 were immersion-fixed in 2.5% glutaraldehyde and 1% formaldehyde in 0.1 M phosphate buffer (pH 7.4) for 1 h at RT and stored at 4°C. Samples were rinsed and post-fixed in 2% OsO_4 in 0.1 M phosphate buffer for 2 h at 4°C, dehydrated through graded ethanol and acetone, and embedded in LX-112 resin (Ladd Research Industries). Ultrathin sections (80–100 nm) were cut using a Leica EM UC7 ultramicrotome, mounted on formvar-stabilized slot grids, and contrasted with uranyl acetate and lead citrate. Imaging was performed on a Hitachi HT7700 TEM at 80 kV with a 2k × 2k Veleta CCD camera (Olympus SIS).

Dynamic GSIS assay

Dynamic GSIS was measured using a Biorep PERI-4.2 perifusion system with SC-islets. Effluent was collected at regular intervals, and insulin concentrations were quantified using an AlphaLISA kit. Detailed protocols are provided in supplemental methods.

scRNA-seq

S6 SC-islets from H1 and HS980 cells, and post-transplant grafts, were dissociated into single cells and processed for scRNA-seq using 10× Genomics Chromium Next GEM 3′ kits. Libraries were sequenced on an Illumina NextSeq 2000, and data were aligned to the human reference genome (GRCh38) with mouse reads removed for graft samples. High-quality cells were retained for downstream analysis with Seurat package (v5.1.0) (Hao et al., 2021), with clustering, UMAP visualization, and differential gene expression performed as described in supplemental methods. Integration and batch correction of pre- and post-transplant datasets, as well as comparisons to published differentiation protocols, were conducted using fastMNN and multiBatchNorm from SeuratWrappers package (v0.3.5) and batchelor package (v1.18.1) (Haghverdi et al., 2018), respectively. Proliferation and β-cell maturation scores were computed with AddModuleScore from Seurat package. RNA velocity analysis was performed on HS980 and grafted cells using scvelo. Detailed sample preparation, analysis pipelines, and parameters are provided in supplemental methods.

Transplantation studies

All animal procedures were approved by Regional Ethical Committee at Karolinska Institutet. Six- to seven-week-old NSG mice (Jackson Laboratories) were maintained under standard conditions. Diabetes was induced at 8 weeks by intraperitoneal injection (i.p.) of STZ (60 mg/kg body weight, 4 consecutive days). Non-fasting blood glucose was monitored throughout, and monthly tail-vein samples were used to measure human and mouse c-peptide by species-specific ELISA.

Diabetic NSG mice were anesthetized with isoflurane, and HS980 SC-islets (280–300 per eye) were transplanted

into the anterior chamber via a corneal puncture using a glass microcannula connected to a syringe. Postoperative care included ocular lubrication and subcutaneous analgesia (Temgesic, 0.1 μg/g). Mice were supported with long-acting insulin (Insulatard Penfill, Novo Nordisk) at 0.05–0.15 IU s.c. from days 4–5 post-transplant for 45–56 days. Glucose responsiveness was assessed by IPGTT after 5 h fasting, measuring blood glucose at 0, 15, 30, 60, and 120 min and plasma c-peptide at 0, 30, 60, and 120 min. Detailed procedures are provided in supplemental methods.

Statistical analysis

Data are shown as mean ± SD or SEM (n = independent experiments). Two-tailed paired or unpaired t tests were used for two-group comparisons; one-way ANOVA with Tukey's test or two-way ANOVA with Bonferroni's test was applied for multiple groups or time points. Significance was set at $p < 0.05$. Analyses were performed in GraphPad Prism and Excel.

RESOURCE AVAILABILITY

Lead contact

Requests for further information and resources should be directed to and will be fulfilled by the lead contact, Siqin Wu (siqinw@gmail.com).

Materials availability

This study did not generate new unique reagents.

Data and code availability

The accession number for the single-cell RNA sequencing data reported in this paper is Gene Expression Omnibus: GSE270864. Analysis code is available at https://github.com/kblust/SC-islets.

ACKNOWLEDGMENTS

Open access funding was provided by Karolinska Institutet. This work was supported by VINNOVA; Spiber Technologies AB; the Swedish Research Council; Swedish Foundation for Strategic Research; Ragnar Söderberg Foundation; Ming Wai Lau Center for Reparative Medicine; Wallenberg Academy Fellow; Knut and Alice Wallenberg Foundation; Barndiabetesfonden; National ATMP Research School; Family Erling-Persson Foundation; Novo Nordisk Foundation; Jonas & Christina af Jochnick Foundation; Swedish Diabetes Association; ERC grant ERC-2018-AdG 834860 EYELETS; and Karolinska Institutet Strategic Research Programs. Imaging was performed at the Live Cell Imaging Unit/Nikon Center of Excellence and BioNut. Flow cytometry was performed at MedH Flow Cytometry core facility, and prenatal human tissue was provided by Developmental Tissue Bank. Sequencing was conducted at ESCG Infrastructure and Bioinformatics units at Science for Life Laboratory. Transmission electron microscopy was performed at the electron microscopy core facility (EMil) at the Department of Laboratory Medicine Karolinska Institute.

AUTHOR CONTRIBUTIONS

S.W., M.H., P.-O.B., and F.L. conceived the study; S.W., S.C., P.E., and E.E. conducted *in vitro* experiments; G.B. performed *in vivo* studies; C.Z. and K.B. analyzed scRNA-seq data; S.W., G.B., S.C., C.Z., and K.B. assembled and interpreted the data; S.W. wrote the original draft. All authors reviewed, edited, and approved the manuscript.

DECLARATION OF INTERESTS

S.W. is the inventor on patent applications (WO2024033299A1 and WO2024033300A1) related to the pancreatic differentiation protocol described in this paper. S.W. is employed by Spiber Technologies AB. P.E. was employed by Spiber Technologies AB. M.H. holds shares in Spiber Technologies AB. P.-O.B. is CEO of Biocrine AB.

SUPPLEMENTAL INFORMATION

Supplemental information can be found online at https://doi.org/10.1016/j.stemcr.2026.102892.

Received: August 9, 2025
Revised: March 16, 2026
Accepted: March 17, 2026
Published: April 16, 2026

REFERENCES

Aghazadeh, Y., Sarangi, F., Poon, F., Nkennor, B., McGaugh, E.C., Nunes, S.S., and Nostro, M.C. (2022). GP2-enriched pancreatic progenitors give rise to functional beta cells in vivo and eliminate the risk of teratoma formation. Stem Cell Rep. *17*, 964–978.

Augsornworawat, P., Hogrebe, N.J., Ishahak, M., Schmidt, M.D., Marquez, E., Maestas, M.M., Veronese-Paniagua, D.A., Gale, S.E., Miller, J.R., Velazco-Cruz, L., and Millman, J.R. (2023). Single-nucleus multi-omics of human stem cell-derived islets identifies deficiencies in lineage specification. Nat. Cell Biol. *25*, 904–916.

Augsornworawat, P., Maxwell, K.G., Velazco-Cruz, L., and Millman, J.R. (2020). Single-Cell Transcriptome Profiling Reveals β Cell Maturation in Stem Cell-Derived Islets after Transplantation. Cell Rep. *32*, 108067.

Balboa, D., Barsby, T., Lithovius, V., Saarimäki-Vire, J., Omar-Hmeadi, M., Dyachok, O., Montaser, H., Lund, P.E., Yang, M., Ibrahim, H., et al. (2022). Functional, metabolic and transcriptional maturation of human pancreatic islets derived from stem cells. Nat. Biotechnol. *40*, 1042–1055.

Barsby, T., Ibrahim, H., Lithovius, V., Montaser, H., Balboa, D., Vähäkangas, E., Chandra, V., Saarimäki-Vire, J., and Otonkoski, T. (2022). Differentiating functional human islet-like aggregates from pluripotent stem cells. STAR Protoc. *3*, 101711.

Berggren, P.O., Yang, S.N., and Shi, Y. (2024). The anterior chamber of the eye technology and its anatomical, optical and immunological bases. Physiol. Rev. *104*, 881–929.

Bohuslavova, R., Smolik, O., Malfatti, J., Berkova, Z., Novakova, Z., Saudek, F., and Pavlinkova, G. (2021). NEUROD1 Is Required for

the Early α and β Endocrine Differentiation in the Pancreas. Int. J. Mol. Sci. *22*, 6713.

Brennan, D.C., Kopetskie, H.A., Sayre, P.H., Alejandro, R., Cagliero, E., Shapiro, A.M.J., Goldstein, J.S., DesMarais, M.R., Booher, S., and Bianchine, P.J. (2016). Long-Term Follow-Up of the Edmonton Protocol of Islet Transplantation in the United States. Am. J. Transplant. *16*, 509–517.

Cogger, K.F., Sinha, A., Sarangi, F., McGaugh, E.C., Saunders, D., Dorrell, C., Mejia-Guerrero, S., Aghazadeh, Y., Rourke, J.L., Screaton, R.A., et al. (2017). Glycoprotein 2 is a specific cell surface marker of human pancreatic progenitors. Nat. Commun. *8*, 331.

D'Amour, K.A., Bang, A.G., Eliazer, S., Kelly, O.G., Agulnick, A.D., Smart, N.G., Moorman, M.A., Kroon, E., Carpenter, M.K., and Baetge, E.E. (2006). Production of pancreatic hormone-expressing endocrine cells from human embryonic stem cells. Nat. Biotechnol. *24*, 1392–1401.

Davis, J.C., Alves, T.C., Helman, A., Chen, J.C., Kenty, J.H., Cardone, R.L., Liu, D.R., Kibbey, R.G., and Melton, D.A. (2020). Glucose Response by Stem Cell-Derived β Cells In Vitro Is Inhibited by a Bottleneck in Glycolysis. Cell Rep. *31*, 107623.

Díaz-Catalán, D., Alcarraz-Vizán, G., Castaño, C., de Pablo, S., Rodríguez-Comas, J., Fernández-Pérez, A., Vallejo, M., Ramírez, S., Claret, M., Parrizas, M., et al. (2021). BACE2 suppression in mice aggravates the adverse metabolic consequences of an obesogenic diet. Mol. Metab. *53*, 101251.

Entezari, M., Hashemi, D., Taheriazam, A., Zabolian, A., Mohammadi, S., Fakhri, F., Hashemi, M., Hushmandi, K., Ashrafizadeh, M., Zarrabi, A., et al. (2022). AMPK signaling in diabetes mellitus, insulin resistance and diabetic complications: A pre-clinical and clinical investigation. Biomed. Pharmacother. *146*, 112563.

Gerace, D., Zhou, Q., Kenty, J.H.R., Veres, A., Sintov, E., Wang, X., Boulanger, K.R., Li, H., and Melton, D.A. (2023). Engineering human stem cell-derived islets to evade immune rejection and promote localized immune tolerance. Cell Rep. Med. *4*, 100879.

Gosmain, Y., Katz, L.S., Masson, M.H., Cheyssac, C., Poisson, C., and Philippe, J. (2012). Pax6 is crucial for β-cell function, insulin biosynthesis, and glucose-induced insulin secretion. Mol. Endocrinol. *26*, 696–709.

Gouzi, M., Kim, Y.H., Katsumoto, K., Johansson, K., and Grapin-Botton, A. (2011). Neurogenin3 initiates stepwise delamination of differentiating endocrine cells during pancreas development. Dev. Dyn. *240*, 589–604.

Haghverdi, L., Lun, A.T.L., Morgan, M.D., and Marioni, J.C. (2018). Batch effects in single-cell RNA-sequencing data are corrected by matching mutual nearest neighbors. Nat. Biotechnol. *36*, 421–427.

Hao, Y., Hao, S., Andersen-Nissen, E., Mauck, W.M., Zheng, S., Butler, A., Lee, M.J., Wilk, A.J., Darby, C., Zager, M., et al. (2021). Integrated analysis of multimodal single-cell data. Cell *184*, 3573–3587.e29.

Hogrebe, N.J., Augsornworawat, P., Maxwell, K.G., Velazco-Cruz, L., and Millman, J.R. (2020). Targeting the cytoskeleton to direct pancreatic differentiation of human pluripotent stem cells. Nat. Biotechnol. *38*, 460–470.

Hogrebe, N.J., Maxwell, K.G., Augsornworawat, P., and Millman, J.R. (2021). Generation of insulin-producing pancreatic β cells from multiple human stem cell lines. Nat. Protoc. *16*, 4109–4143.

Hu, X., Gattis, C., Olroyd, A.G., Friera, A.M., White, K., Young, C., Basco, R., Lamba, M., Wells, F., Ankala, R., et al. (2023). Human hypoimmune primary pancreatic islets avoid rejection and autoimmunity and alleviate diabetes in allogeneic humanized mice. Sci. Transl. Med. *15*, eadg5794.

Jeon, J., Correa-Medina, M., Ricordi, C., Edlund, H., and Diez, J.A. (2009). Endocrine cell clustering during human pancreas development. J. Histochem. Cytochem. *57*, 811–824.

Kassem, S.A., Ariel, I., Thornton, P.S., Scheimberg, I., and Glaser, B. (2000). Beta-cell proliferation and apoptosis in the developing normal human pancreas and in hyperinsulinism of infancy. Diabetes *49*, 1325–1333.

Kelly, O.G., Chan, M.Y., Martinson, L.A., Kadoya, K., Ostertag, T.M., Ross, K.G., Richardson, M., Carpenter, M.K., D'Amour, K.A., Kroon, E., et al. (2011). Cell-surface markers for the isolation of pancreatic cell types derived from human embryonic stem cells. Nat. Biotechnol. *29*, 750–756.

Kroon, E., Martinson, L.A., Kadoya, K., Bang, A.G., Kelly, O.G., Eliazer, S., Young, H., Richardson, M., Smart, N.G., Cunningham, J., et al. (2008). Pancreatic endoderm derived from human embryonic stem cells generates glucose-responsive insulin-secreting cells in vivo. Nat. Biotechnol. *26*, 443–452.

Lithovius, V., Lahdenpohja, S., Ibrahim, H., Saarimäki-Vire, J., Uusitalo, L., Montaser, H., Mikkola, K., Yim, C.B., Keller, T., Rajander, J., et al. (2024). Non-invasive quantification of stem cell-derived islet graft size and composition. Diabetologia *67*, 1912–1929.

Lyon, J., Manning Fox, J.E., Spigelman, A.F., Kim, R., Smith, N., O'Gorman, D., Kin, T., Shapiro, A.M.J., Rajotte, R.V., and MacDonald, P.E. (2016). Research-Focused Isolation of Human Islets From Donors With and Without Diabetes at the Alberta Diabetes Institute IsletCore. Endocrinology *157*, 560–569.

Main, H., Hedenskog, M., Acharya, G., Hovatta, O., and Lanner, F. (2020). Karolinska Institutet Human Embryonic Stem Cell Bank. Stem Cell Res. *45*, 101810.

Mamidi, A., Prawiro, C., Seymour, P.A., de Lichtenberg, K.H., Jackson, A., Serup, P., and Semb, H. (2018). Mechanosignalling via integrins directs fate decisions of pancreatic progenitors. Nature *564*, 114–118.

Mastracci, T.L., Anderson, K.R., Papizan, J.B., and Sussel, L. (2013). Regulation of Neurod1 contributes to the lineage potential of Neurogenin3+ endocrine precursor cells in the pancreas. PLoS Genet. *9*, e1003278.

Maxwell, K.G., Kim, M.H., Gale, S.E., and Millman, J.R. (2022). Differential Function and Maturation of Human Stem Cell-Derived Islets After Transplantation. Stem Cells Transl. Med. *11*, 322–331.

Nair, G., and Hebrok, M. (2015). Islet formation in mice and men: lessons for the generation of functional insulin-producing β-cells

from human pluripotent stem cells. Curr. Opin. Genet. Dev. *32*, 171–180.

Nostro, M.C., Sarangi, F., Yang, C., Holland, A., Elefanty, A.G., Stanley, E.G., Greiner, D.L., and Keller, G. (2015). Efficient generation of NKX6-1+ pancreatic progenitors from multiple human pluripotent stem cell lines. Stem Cell Rep. *4*, 591–604.

Pagliuca, F.W., Millman, J.R., Gürtler, M., Segel, M., Van Dervort, A., Ryu, J.H., Peterson, Q.P., Greiner, D., and Melton, D.A. (2014). Generation of functional human pancreatic β cells in vitro. Cell *159*, 428–439.

Peterson, Q.P., Veres, A., Chen, L., Slama, M.Q., Kenty, J.H.R., Hassoun, S., Brown, M.R., Dou, H., Duffy, C.D., Zhou, Q., et al. (2020). A method for the generation of human stem cell-derived alpha cells. Nat. Commun. *11*, 2241.

Plaza Reyes, A., Petrus-Reurer, S., Padrell Sánchez, S., Kumar, P., Douagi, I., Bartuma, H., Aronsson, M., Westman, S., Lardner, E., André, H., et al. (2020). Identification of cell surface markers and establishment of monolayer differentiation to retinal pigment epithelial cells. Nat. Commun. *11*, 1609.

Rajaei, B., Garcia, A.M., Juksar, J., Doppenberg, J.B., Paz-Barba, M., Boot, F., de Vos, W., Mulder, A.A., Lambregtse, F., Daleman, L., et al. (2025). Clinically compliant enrichment of human pluripotent stem cell-derived islets. Sci. Transl. Med. *17*, eadl4390.

Ramzy, A., Thompson, D.M., Ward-Hartstonge, K.A., Ivison, S., Cook, L., Garcia, R.V., Loyal, J., Kim, P.T.W., Warnock, G.L., Levings, M.K., and Kieffer, T.J. (2021). Implanted pluripotent stem-cell-derived pancreatic endoderm cells secrete glucose-responsive C-peptide in patients with type 1 diabetes. Cell Stem Cell *28*, 2047–2061.e5.

Reichman, T.W., Markmann, J.F., Odorico, J., Witkowski, P., Fung, J.J., Wijkstrom, M., Kandeel, F., de Koning, E.J.P., Peters, A.L., Mathieu, C., et al. (2025). Stem Cell-Derived, Fully Differentiated Islets for Type 1 Diabetes. N. Engl. J. Med. *393*, 858–868.

Rezania, A., Bruin, J.E., Arora, P., Rubin, A., Batushansky, I., Asadi, A., O'Dwyer, S., Quiskamp, N., Mojibian, M., Albrecht, T., et al. (2014). Reversal of diabetes with insulin-producing cells derived in vitro from human pluripotent stem cells. Nat. Biotechnol. *32*, 1121–1133.

Rezania, A., Bruin, J.E., Riedel, M.J., Mojibian, M., Asadi, A., Xu, J., Gauvin, R., Narayan, K., Karanu, F., O'Neil, J.J., et al. (2012). Maturation of human embryonic stem cell-derived pancreatic progenitors into functional islets capable of treating pre-existing diabetes in mice. Diabetes *61*, 2016–2029.

Rodin, S., Antonsson, L., Niaudet, C., Simonson, O.E., Salmela, E., Hansson, E.M., Domogatskaya, A., Xiao, Z., Damdimopoulou, P., Sheikhi, M., et al. (2014). Clonal culturing of human embryonic stem cells on laminin-521/E-cadherin matrix in defined and xeno-free environment. Nat. Commun. *5*, 3195.

Rodriguez-Diaz, R., Molano, R.D., Weitz, J.R., Abdulreda, M.H., Berman, D.M., Leibiger, B., Leibiger, I.B., Kenyon, N.S., Ricordi, C., Pileggi, A., et al. (2018). Paracrine Interactions within the Pancreatic Islet Determine the Glycemic Set Point. Cell Metab. *27*, 549–558.e4.

Rosado-Olivieri, E.A., Anderson, K., Kenty, J.H., and Melton, D.A. (2019). YAP inhibition enhances the differentiation of functional stem cell-derived insulin-producing β cells. Nat. Commun. *10*, 1464.

Shapiro, A.M., Lakey, J.R., Ryan, E.A., Korbutt, G.S., Toth, E., Warnock, G.L., Kneteman, N.M., and Rajotte, R.V. (2000). Islet transplantation in seven patients with type 1 diabetes mellitus using a glucocorticoid-free immunosuppressive regimen. N. Engl. J. Med. *343*, 230–238.

Sharon, N., Chawla, R., Mueller, J., Vanderhooft, J., Whitehorn, L.J., Rosenthal, B., Gürtler, M., Estanboulieh, R.R., Shvartsman, D., Gifford, D.K., et al. (2019a). A Peninsular Structure Coordinates Asynchronous Differentiation with Morphogenesis to Generate Pancreatic Islets. Cell *176*, 790–804.e13.

Sharon, N., Vanderhooft, J., Straubhaar, J., Mueller, J., Chawla, R., Zhou, Q., Engquist, E.N., Trapnell, C., Gifford, D.K., and Melton, D.A. (2019b). Wnt Signaling Separates the Progenitor and Endocrine Compartments during Pancreas Development. Cell Rep. *27*, 2281–2291.e5.

Sintov, E., Nikolskiy, I., Barrera, V., Hyoje-Ryu Kenty, J., Atkin, A.S., Gerace, D., Ho Sui, S.J., Boulanger, K., and Melton, D.A. (2022). Whole-genome CRISPR screening identifies genetic manipulations to reduce immune rejection of stem cell-derived islets. Stem Cell Rep. *17*, 1976–1990.

Speier, S., Nyqvist, D., Cabrera, O., Yu, J., Molano, R.D., Pileggi, A., Moede, T., Köhler, M., Wilbertz, J., Leibiger, B., et al. (2008a). Noninvasive in vivo imaging of pancreatic islet cell biology. Nat. Med. *14*, 574–578.

Speier, S., Nyqvist, D., Köhler, M., Caicedo, A., Leibiger, I.B., and Berggren, P.O. (2008b). Noninvasive high-resolution in vivo imaging of cell biology in the anterior chamber of the mouse eye. Nat. Protoc. *3*, 1278–1286.

Svendsen, B., Larsen, O., Gabe, M.B.N., Christiansen, C.B., Rosenkilde, M.M., Drucker, D.J., and Holst, J.J. (2018). Insulin Secretion Depends on Intra-islet Glucagon Signaling. Cell Rep. *25*, 1127–1134.e2.

Tuluc, P., Theiner, T., Jacobo-Piqueras, N., and Geisler, S.M. (2021). Role of High Voltage-Gated Ca. Cells *10*, 2004.

Velazco-Cruz, L., Song, J., Maxwell, K.G., Goedegebuure, M.M., Augsornworawat, P., Hogrebe, N.J., and Millman, J.R. (2019). Acquisition of Dynamic Function in Human Stem Cell-Derived β Cells. Stem Cell Rep. *12*, 351–365.

Veres, A., Faust, A.L., Bushnell, H.L., Engquist, E.N., Kenty, J.H.R., Harb, G., Poh, Y.C., Sintov, E., Gürtler, M., Pagliuca, F.W., et al. (2019). Charting cellular identity during human in vitro β-cell differentiation. Nature *569*, 368–373.

Wang, S., Du, Y., Zhang, B., Meng, G., Liu, Z., Liew, S.Y., Liang, R., Zhang, Z., Cai, X., Wu, S., et al. (2024a). Transplantation of chemically induced pluripotent stem-cell-derived islets under abdominal anterior rectus sheath in a type 1 diabetes patient. Cell *187*, 6152–6164.e18.

Wang, Z., Dong, S., and Zhou, W. (2024b). Pancreatic stellate cells: Key players in pancreatic health and diseases. Mol. Med. Rep. *30*, 109.

Wong, Y.F., Kumar, Y., Proks, M., Herrera, J.A.R., Rothová, M.M., Monteiro, R.S., Pozzi, S., Jennings, R.E., Hanley, N.A., Bickmore, W.A., and Brickman, J.M. (2023). Expansion of ventral foregut is linked to changes in the enhancer landscape for organ-specific differentiation. Nat. Cell Biol. *25*, 481–492.

Yoon, J.S., Sasaki, S., Velghe, J., Lee, M.Y.Y., Winata, H., Nian, C., and Lynn, F.C. (2022). Calcium-dependent transcriptional changes in human pancreatic islet cells reveal functional diversity in islet cell subtypes. Diabetologia *65*, 1519–1533.

Zhu, H., Wang, G., Nguyen-Ngoc, K.V., Kim, D., Miller, M., Goss, G., Kovsky, J., Harrington, A.R., Saunders, D.C., Hopkirk, A.L., et al. (2023). Understanding cell fate acquisition in stem-cell-derived pancreatic islets using single-cell multiome-inferred regulomes. Dev. Cell *58*, 727–743.e11.

Zhu, L., Dattaroy, D., Pham, J., Wang, L., Barella, L.F., Cui, Y., Wilkins, K.J., Roth, B.L., Hochgeschwender, U., Matschinsky, F.M., et al. (2019). Intra-islet glucagon signaling is critical for maintaining glucose homeostasis. JCI Insight *5*, e127994.

Stem Cell Reports, Volume *21*

Supplemental Information

An optimized protocol for efficient derivation of pancreatic islets from multiple human pluripotent stem cell lines

Siqin Wu, Shivam Chandel, Galyna Bryzgalova, Paschalis Efstathopoulos, Kelly Blust, Cheng Zhao, Eda Erbil, Anna Falk, My Hedhammar, Per-Olof Berggren, and Fredrik Lanner

Supplemental Information

An optimized protocol for efficient derivation of pancreatic islets from multiple human pluripotent stem cell lines

Siqin Wu, Shivam Chandel, Galyna Bryzgalova, Paschalis Efstathopoulos, Kelly Blust, Cheng Zhao, Eda Erbil, Anna Falk, My Hedhammar, Per-Olof Berggren, Fredrik Lanner

Figure S1

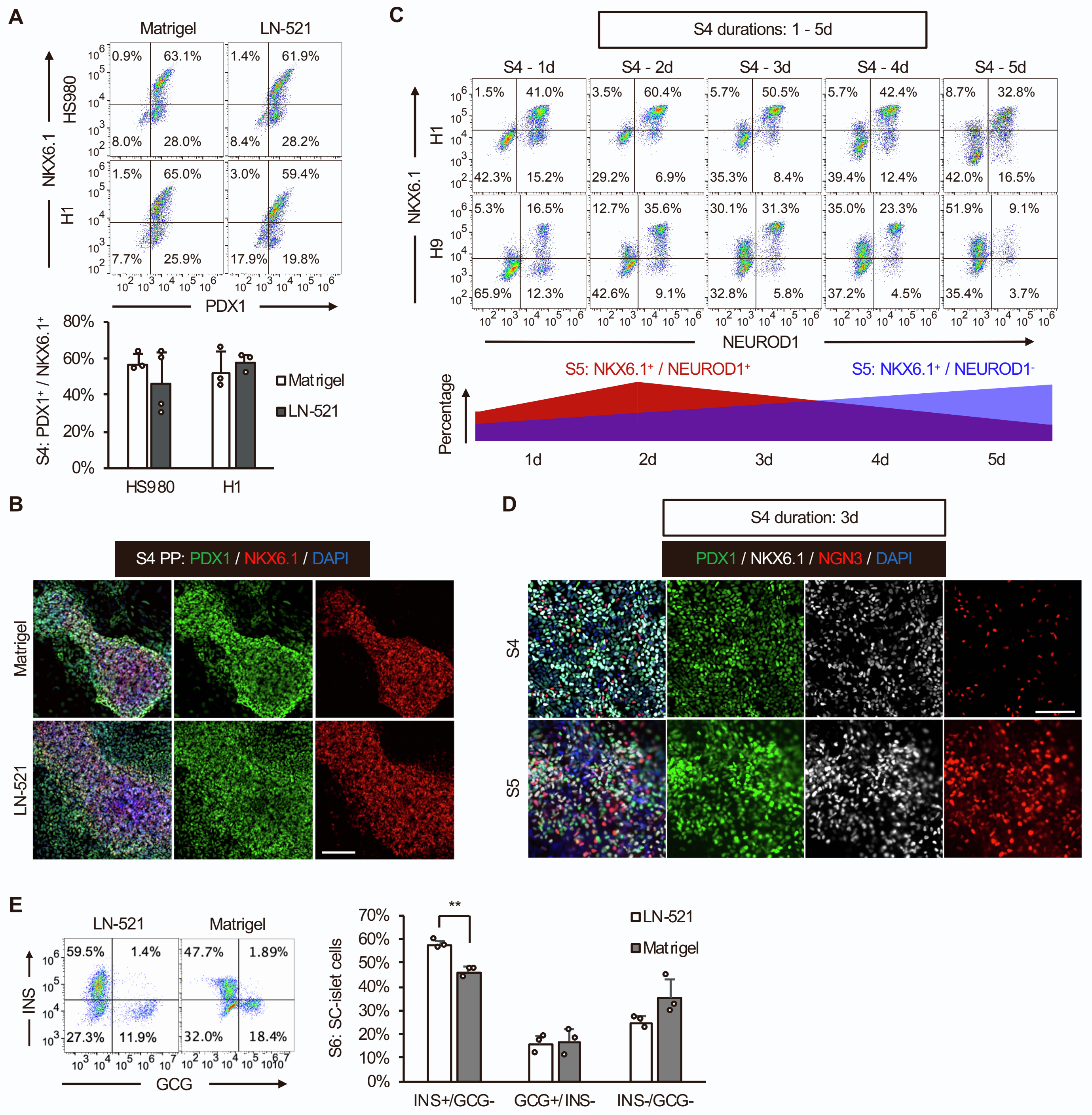

A
Matrigel
LN-521
HS980
H1
NKX6.1
PDX1
0.9% 63.1% 8.0% 28.0%
1.4% 61.9% 8.4% 28.2%
1.5% 65.0% 7.7% 25.9%
3.0% 59.4% 17.9% 19.8%
S4: PDX1⁺ / NKX6.1⁺
80% 60% 40% 20% 0%
Matrigel
LN-521
HS980
H1
B
S4 PP: PDX1 / NKX6.1 / DAPI
Matrigel
LN-521
C
S4 durations: 1 - 5d
S4 - 1d
S4 - 2d
S4 - 3d
S4 - 4d
S4 - 5d
H1
H9
NKX6.1
NEUROD1
1.5% 41.0% 42.3% 15.2%
3.5% 60.4% 29.2% 6.9%
5.7% 50.5% 35.3% 8.4%
5.7% 42.4% 39.4% 12.4%
8.7% 32.8% 42.0% 16.5%
5.3% 16.5% 65.9% 12.3%
12.7% 35.6% 42.6% 9.1%
30.1% 31.3% 32.8% 5.8%
35.0% 23.3% 37.2% 4.5%
51.9% 9.1% 35.4% 3.7%
S5: NKX6.1⁺ / NEUROD1⁺
S5: NKX6.1⁺ / NEUROD1⁻
Percentage
1d 2d 3d 4d 5d
D
S4 duration: 3d
PDX1 / NKX6.1 / NGN3 / DAPI
S4
S5
E
LN-521
Matrigel
INS
GCG
59.5% 1.4% 27.3% 11.9%
47.7% 1.89% 32.0% 18.4%
S6: SC-islet cells
70% 60% 50% 40% 30% 20% 10% 0%
**
LN-521
Matrigel
INS+/GCG-
GCG+/INS-
INS-/GCG-

Figure S1. Pancreatic islet differentiation on recombinant human laminin coatings, related to Figure 1.

(**A** and **B**) Differentiation towards S4 PP on Matrigel and recombinant human laminin (LN) -521 using the long differentiation protocol as described in Methods. The expression of PP markers PDX1 and NKX6.1 were examined at the end of S4 (S4) by flow cytometry and immunocytochemistry (ICC). (**A**) Representative dot plots (upper) and bar graphs (lower) representing the results for both HS980 and H1 cells. Data are means ± SD, n = 3 - 4. (**B**) Fluorescence microscope images showing expression of PDX1 and NKX6.1. n = 3. Scale bar = 100 μM. (**C**) Differentiation towards S5 EP on LN-521 with S4 durations of 1 - 5 days. The expression of EP markers NKX6.1 and NEUROD1 in H1 and H9 cells were examined by flow cytometry at day 4 of S5 (S5d4). Representative dot plots are shown. n = 3 - 4. Results from statistical analysis are shown in Figure 1C. (**D**) Immunofluorescence analysis of expression of EP markers PDX1, NKX6.1, and NGN3 at S4 and S5d4 (S5). The S4 duration is 3 days on LN-521. n = 3. Scale bar = 100 μM. (**E**) Differentiation towards S6 SC-islet using the short differentiation protocol as described in Methods. H1 cells from LN-521 and Matrigel were dissociated into single cells at S5d4 and then aggregated in 3D suspension. Representative dot plots (left) and bar graphs (right) show the expression of INS and GCG at S6w4, as determined by flow cytometry. Data are means ± SD, n = 3. Unpaired 2-tailed t-tests, ** $p < 0.01$.

Figure S2

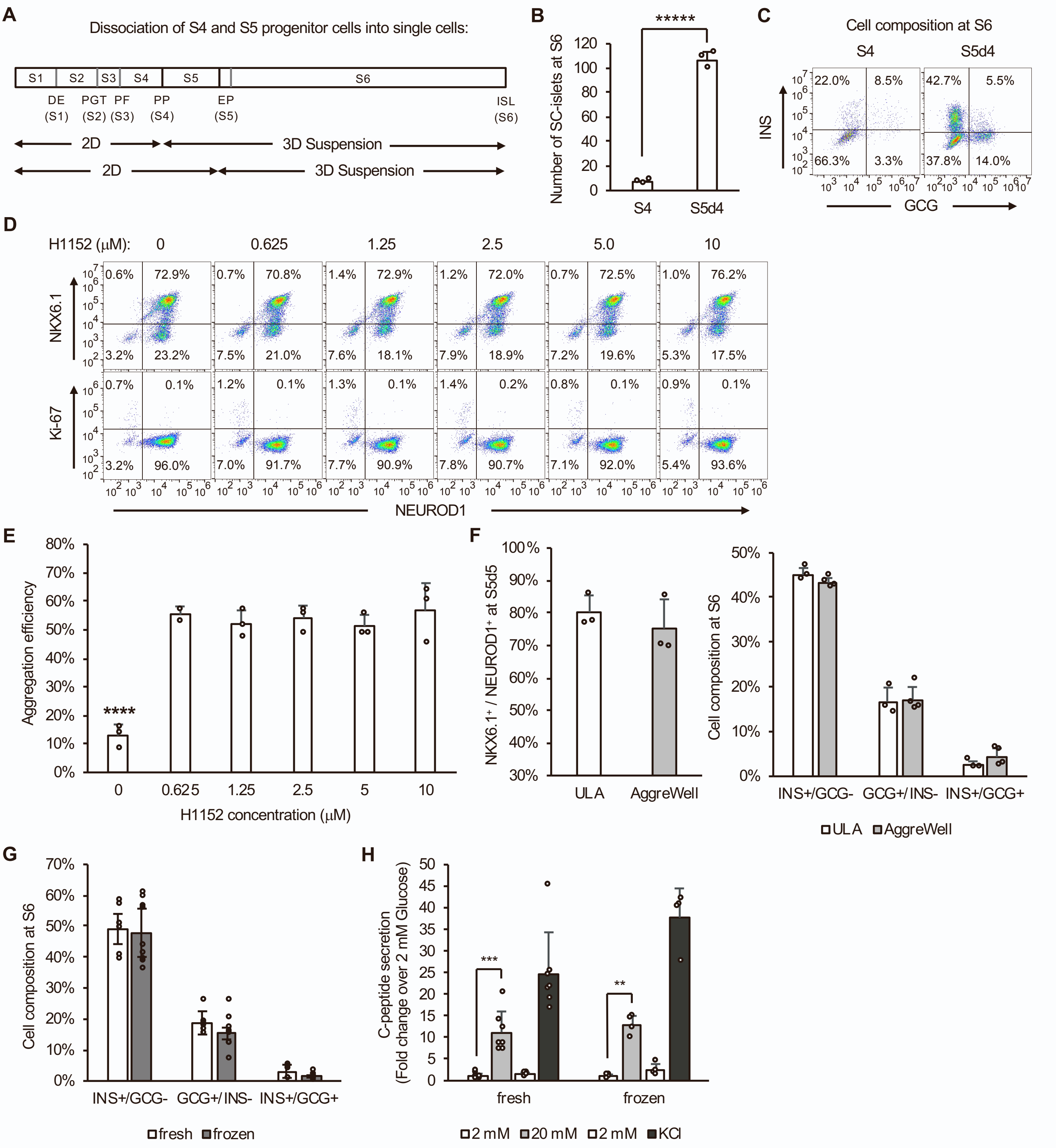
A
Dissociation of S4 and S5 progenitor cells into single cells:
S1 S2 S3 S4 S5 S6
DE (S1) PGT (S2) PF (S3) PP (S4) EP (S5) ISL (S6)
2D
3D Suspension
B
Number of SC-islets at S6
*****
S4
S5d4
C
Cell composition at S6
S4
S5d4
INS
GCG
22.0% 8.5% 66.3% 3.3%
42.7% 5.5% 37.8% 14.0%
D
H1152 (μM): 0 0.625 1.25 2.5 5.0 10
NKX6.1
Ki-67
NEUROD1
E
Aggregation efficiency
****
H1152 concentration (μM)
F
NKX6.1+ / NEUROD1+ at S5d5
ULA
AggreWell
Cell composition at S6
INS+/GCG-
GCG+/INS-
INS+/GCG+
G
Cell composition at S6
fresh
frozen
H
C-peptide secretion (Fold change over 2 mM Glucose)
***
**
2 mM
20 mM
KCl

Figure S2. SC-islet formation of S5 EP cells in 3D suspension culture, related to Figure 2.

(**A**) Schematic representation showing the timeline of differentiation on LN-521. The cells were dissociated into single cells at the end of S4 (S4, upper timeline), or day 4 of S5 (S5d4, lower timeline), and then maintained in ULA-treated well (3D suspension) to generate SC-islets. (**B**) Bar graphs representing the numbers of S6 SC-islets generated from $1x10^6$ single S4 and S5d4 cells. Data are means ± SD, *n* = 3. Unpaired 2-tailed t-test, ***** $p < 0.00001$. (**C**) The expression of islet markers INS and GCG was measured by flow cytometry at the end of S6. Representative dot plots are shown. *n* = 3. (**D** and **E**) Effects of ROCK inhibitor H1152 on 3D aggregate formation at S5d4. Different concentrations of H1152, from 0 to 10 μM, were added to the single cell suspension for 24 hours as indicated. (**D**) The expression of NKX6.1, NEUROD1, and Ki-67 were examined by flow cytometry at S5d5. Representative dot plots are shown. *n* = 3. (**E**) The aggregation efficiencies were examined at S5d5, as described in Methods. Data are means ± SD, *n* = 3. One-way ANOVA, **** $p < 0.0001$. (**F**) The dissociated S5 cells were maintained in ULA-treated well (ULA) or on AggreWell plate (AggreWell) from S5d4. The expression of NKX6.1 and NEUROD1 at S5d5, and INS and GCG at the end of S6, were measured by flow cytometry. Bar graphs show the percentages of $NKX6.1^+/NEUROD1^+$ EP cells at S5d5 (left), and INS^+/GCG^- β, GCG^+/INS^- α, and INS^+/GCG^+ polyhormonal cells at S6 (right). Data are means ± SD, *n* = 3 for ULA, and 3 - 4 for AggreWell. (**G**) Bar graphs show the percentages of INS^+/GCG^- β, GCG^+/INS^- α, and INS^+/GCG^+ polyhormonal cells in SC-islets derived from fresh and frozen S5d4 cells. Data are means ± SD, *n* = 6 for fresh and 9 for frozen. (**H**) Bar graphs show the results of static *in vitro* GSIS, presented as fold change in c-peptide secretion over 2 mM Glucose set to 1. Data are means ± SD, *n* = 7 for fresh, and 4 for frozen. Paired 2-tailed *t*-tests, ** $p < 0.01$, *** $p < 0.001$.

Figure S3

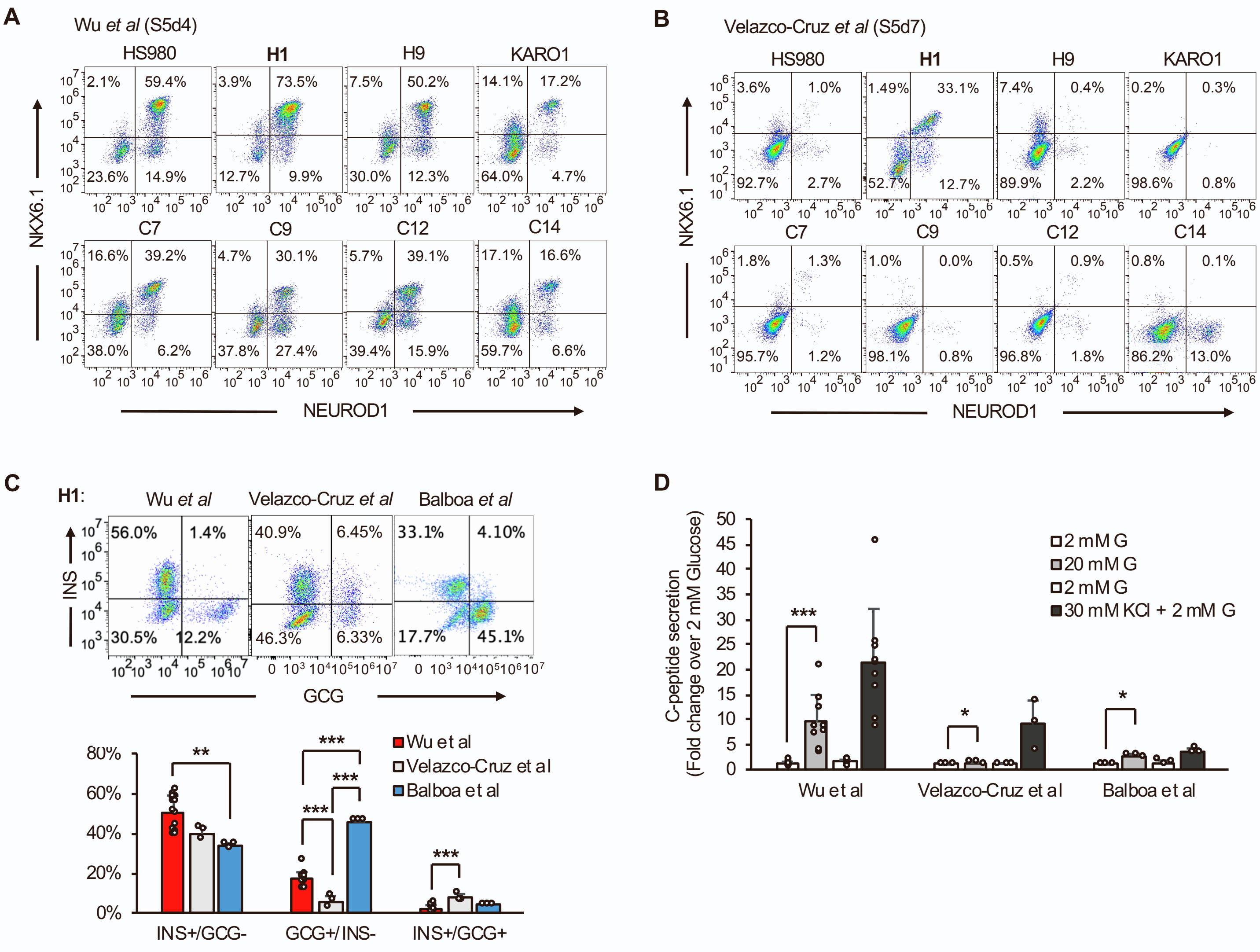

Figure S3. Comparison of three differentiation protocols, related to Figure 3.

Eight human ESC and iPSC lines were differentiated to S5 EP cells using (**A**) the short differentiation protocol as described in Methods (Wu *et al*), and (**B**) the 3D protocol described by Velazco-Cruz *et al* (Velazco-Cruz *et al*). Expressions of EP markers NKX6.1 and NEUROD1 were examined by flow cytometry at S5d4 (Wu *et al*) or S5d7 (Velazco-Cruz *et al*). Representative dot plots are shown. *n* = 7 for HS980 and H1, 3 for H9, KARO1 and C7, and 2 for C9, C12 and C14, all differentiated with Wu *et al. n* = 3 for H1, and 2 for HS980, H9, KARO1, C7, C9, C12, and C14, all differentiated with Velazco-Cruz *et al*. (**C** and **D**) H1 cells were further differentiated using the three differentiation protocols as indicated. (**C**) Expression of islet markers INS and GCG were analyzed by flow cytomtetry at S6. Representative dot plots (upper) and bar graphs (lower) are shown. Data are means ± SD, *n* = 13 (Wu et al), 3 (Velazco-Cruz and Balboa et al). One-way ANOVA, ** $p < 0.01$, *** $p < 0.001$. (**D**) Static GSIS showing fold change in c-peptide over 2 mM glucose. Data are means ± SD, *n* = 9 (Wu et al), 3 (Velazco-Cruz and Balboa et al); paired 2-tailed t-tests, * $p < 0.05$, ** $p < 0.01$, *** $p < 0.001$.

Figure S4

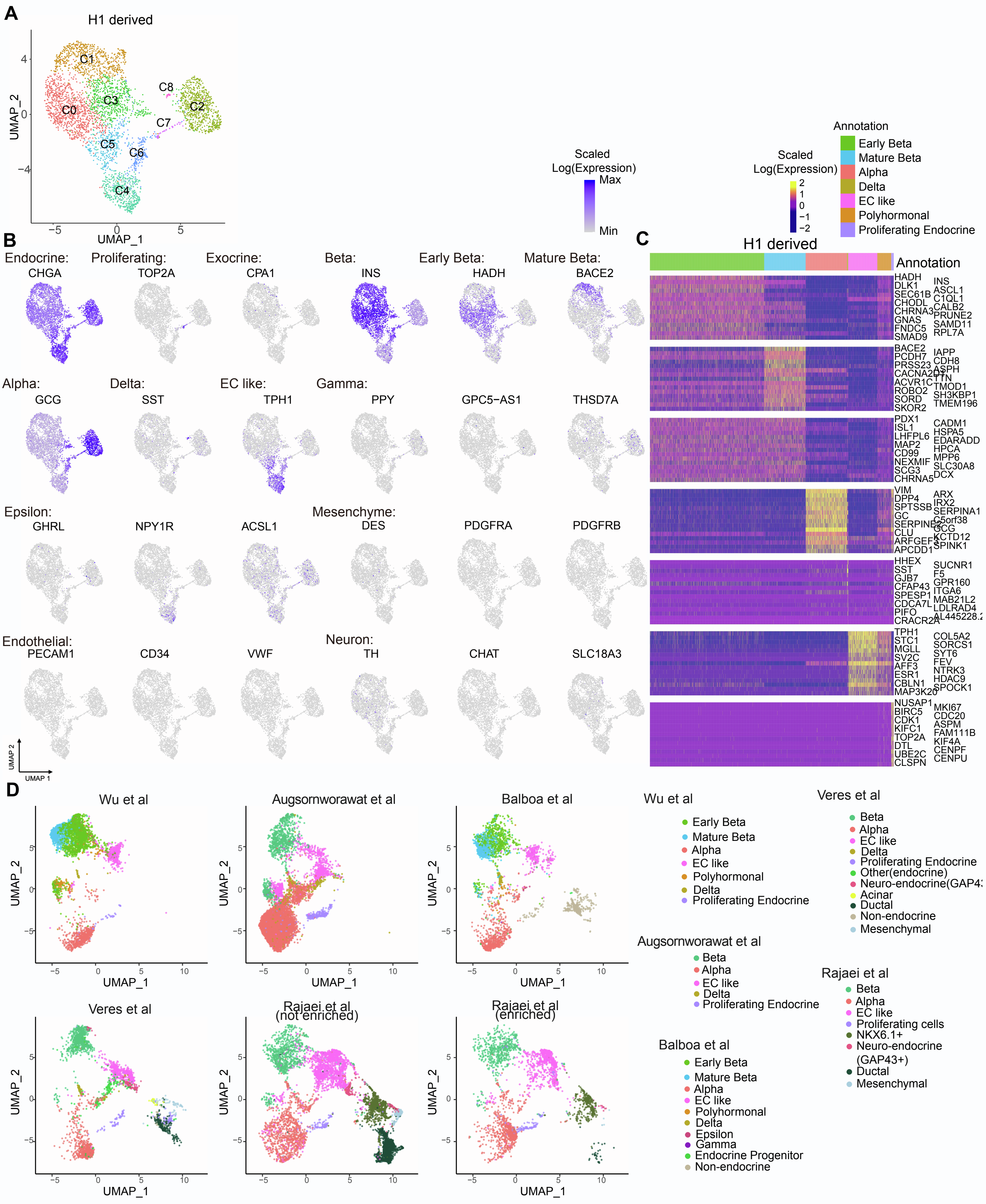
A
H1 derived
C0
C1
C2
C3
C4
C5
C6
C7
C8
UMAP_1
UMAP_2
B
Endocrine: CHGA
Proliferating: TOP2A
Exocrine: CPA1
Beta: INS
Early Beta: HADH
Mature Beta: BACE2
Alpha: GCG
Delta: SST
EC like: TPH1
Gamma: PPY
GPC5−AS1
THSD7A
Epsilon: GHRL
NPY1R
ACSL1
Mesenchyme: DES
PDGFRA
PDGFRB
Endothelial: PECAM1
CD34
VWF
Neuron: TH
CHAT
SLC18A3
Scaled Log(Expression)
Max
Min
C
H1 derived
Annotation
Scaled Log(Expression)
Annotation
Early Beta
Mature Beta
Alpha
Delta
EC like
Polyhormonal
Proliferating Endocrine
D
Wu et al
Augsornworawat et al
Balboa et al
Veres et al
Rajaei et al (not enriched)
Rajaei et al (enriched)
UMAP_1
UMAP_2
Wu et al
Early Beta
Mature Beta
Alpha
EC like
Polyhormonal
Delta
Proliferating Endocrine
Veres et al
Beta
Alpha
EC like
Delta
Proliferating Endocrine
Other(endocrine)
Acinar
Ductal
Non-endocrine
Mesenchymal
Augsornworawat et al
Beta
Alpha
EC like
Delta
Proliferating Endocrine
Rajaei et al
Beta
Alpha
EC like
Proliferating cells
NKX6.1+
Neuro-endocrine (GAP43+)
Ductal
Mesenchymal
Balboa et al
Early Beta
Mature Beta
Alpha
EC like
Polyhormonal
Delta
Epsilon
Gamma
Endocrine Progenitor
Non-endocrine

Figure S4. Transcriptome analysis of SC-islets, related to Figure 4.

(**A**) UMAP projection showing the raw Seurat clusters of SC-islets derived from H1 cell lines at day 43 of differentiation under a resolution of 0.4. (**B**) UMAP plot of cells from H1 SC-islets displaying the expression of endocrine, proliferating, exocrine, beta, alpha, delta, EC-like, gamma, epsilon, Mesenchyme, Endothelial, and neuron markers. (**C**) Heatmap showing the expression of the top 15 marker genes in SC-islets from H1 cell lines at day 43 of differentiation. (**D**) Datasets from different protocols. UMAP projection of integrated datasets, segregated by different protocols, with cells colored according to their original annotations from each dataset.

Figure S5

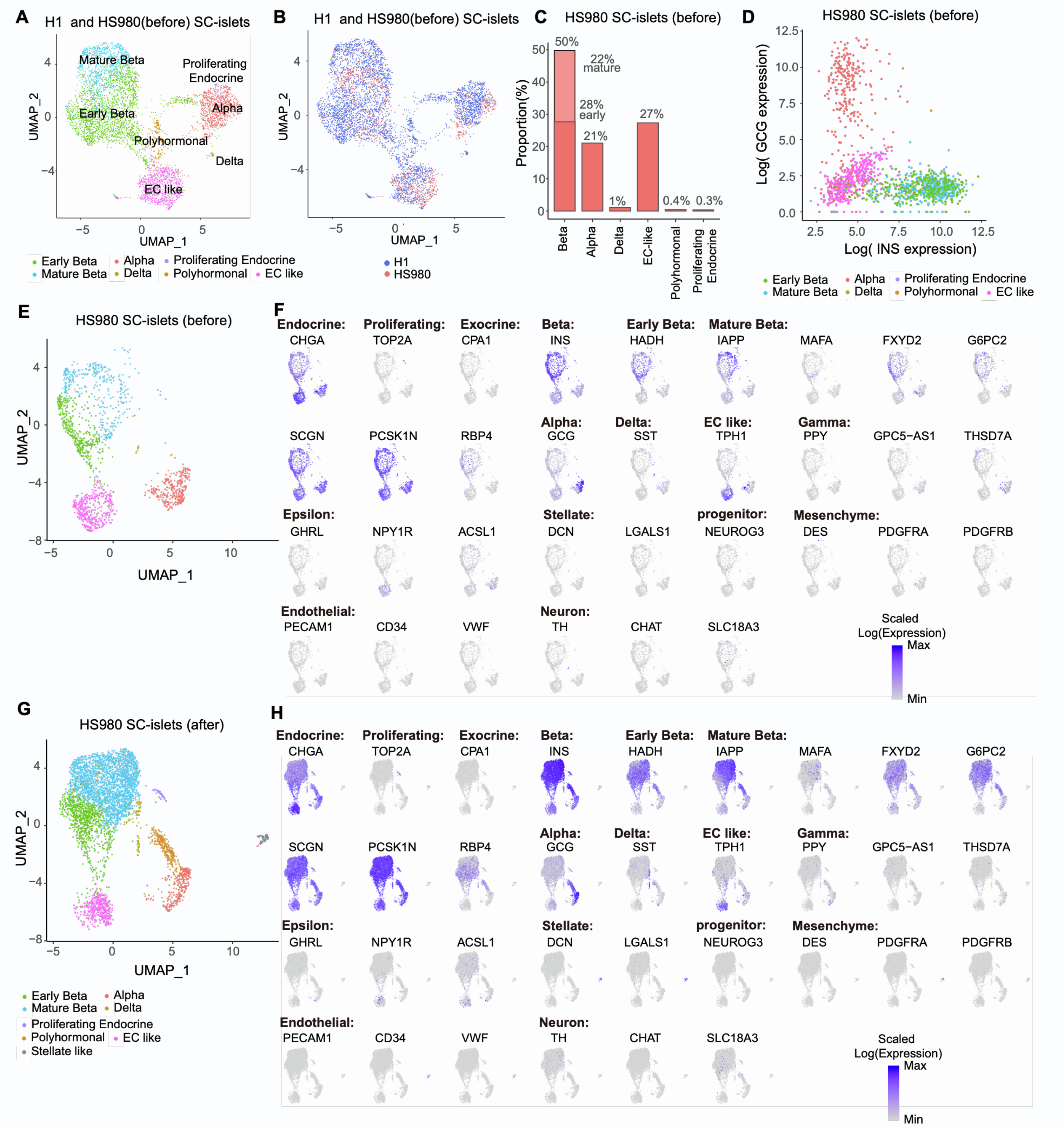

Figure S5. Transcriptome analysis of SC-islets before and after transplantation, related to Figure 6.

(**A** and **B**) UMAP projection showing the integration of SC-islets derived from H1 and HS980 cells before transplantation. Cells are colored by (**A**) annotation from each dataset and (**B**) cell source. (**C**) Bar plot showing the proportion of different cell types in SC-islets derived from HS980 cells before transplantation. (**D**) Dot plot showing expression of INS and GCG in different cell types in SC-islets derived from HS980 cells before transplantation. (**E** and **G**) UMAP projection showing the integration of SC-islets derived from HS980 cells before (**E**) and after (**G**) transplantation. (**F** and **H**) UMAP plots displaying the expression of endocrine, proliferating, exocrine, beta, alpha, delta, EC-like, gamma, epsilon, stellate, endocrine progenitor, mesenchyme, endothelial, and neuron markers in SC-islets before (**F**) and after (**H**) transplantation.

Figure S6

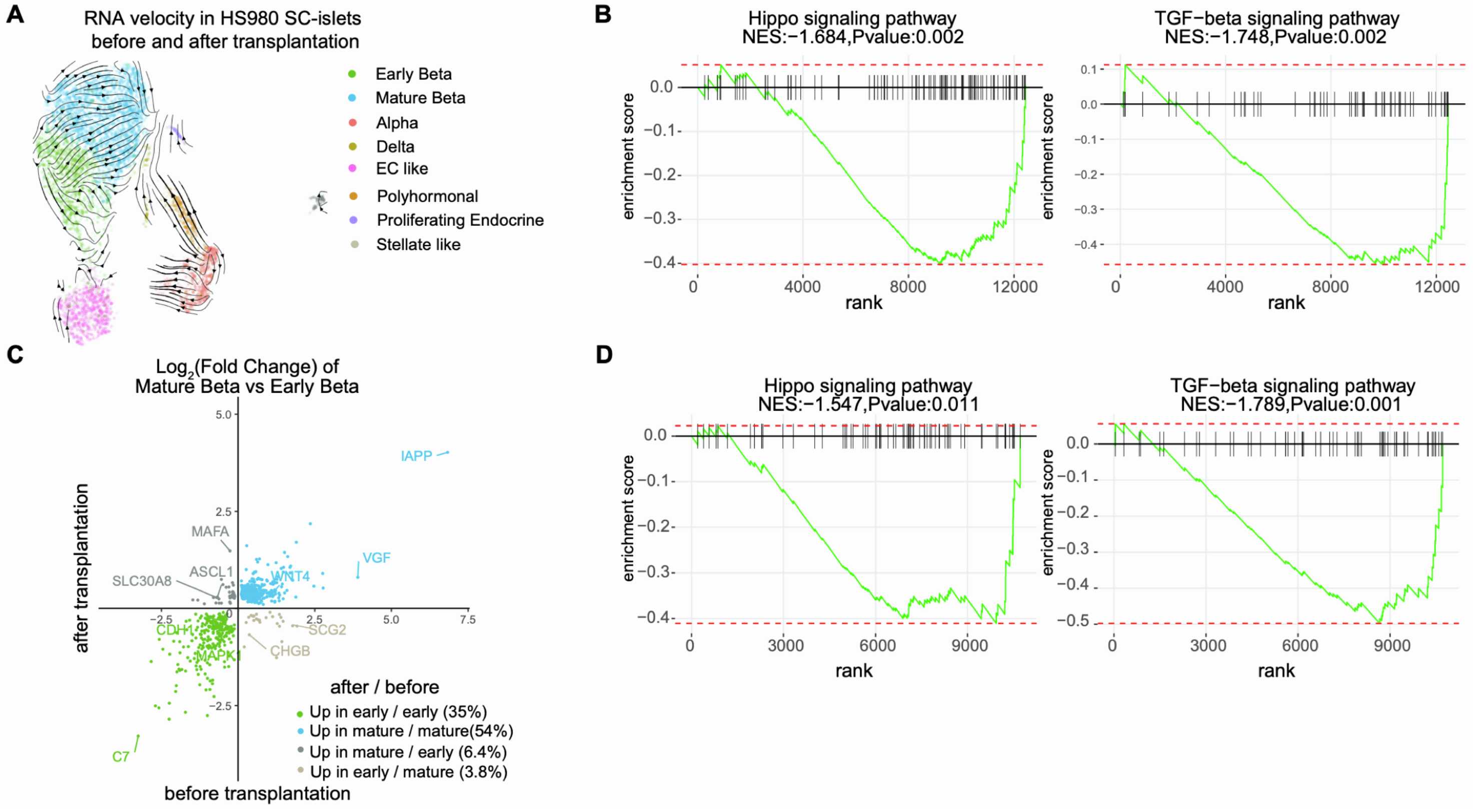

Figure S6. *In vivo* maturation of SC-islets, related to Figure 6.

(**A**) UMAP projection of the cell population post-transplantation with overlaid RNA velocity vectors. (**B** and **D**) Enrichment curves for GSEA results comparing mature beta and early beta cells in the HS980 cell line before (**B**) and after (**D**) transplantation. (**C**) Scatter plot showing the log2 fold change of the union set of differentially expressed genes (DEGs) between mature and early beta cells before transplantation (x-axis) and after transplantation (y-axis). Genes are colored based on the beta cell type (mature or early) in which they have higher expression before and after transplantation.

Table S1: Comparison of protocols for pancreatic islet differentiation.

			Wu *et al*	Hogrebe *et al* (*12, 13, 17, 18*)	Balboa *et al* (*11, 14, 16*)	Velazco-Cruz *et al* (*9*)	Rajaei *et al* (*15*)
2D planar or 3D suspension protocol			2D to 3D transition at S5	2D	2D to 3D transition at S4	3D	3D
SC-islet cell / initial stem cell			1.42 or 2.73	1 (*13*)	1 (*14*)	N/D	N/D
hPSC lines used for generation of SC-islets	hESC		**HS980, H1,** H9, KARO1	**HUES8**, H1	**H1**, H9	**HUES8**	**HUES8**, **RC9**
	WT iPSC		C7, C9, C12, C14	1013-4FA, 1016SeVA, AN1.1, BJFF.6	HEL24.3, HEL113, HEL118.3	1013-4FA, 1016SeVA	LUMC iPSC1, iPSC2, iPSC3
	Diabetic iPSC			1026-3FC, 1031SeVA, T2D001A		1019SeVA	
	Others			WS4			
Differentiation efficiencies	S4 NKX6.1$^+$		50-60%	>40% (*13*)	>60% (*14*)	N/D	N/D
	S5 NEUROD1$^+$		70-90%	N/D	N/D	N/D	N/D
	S6-7 Islet	INS$^+$/GCG$^-$	40-60%	30-55% (*12, 13*)	30-50% (*11, 14*)	40-50% (*9*)	56.4% (*15*)
		GCG$^+$/INS$^-$	15-20%	3-7% (*13*)	30-50% (*11, 14*)	<5% (*9*)	8.5% (*15*)
		INS$^+$/GCG$^+$	<5%	7-15% (*12, 13*)	<5% (*14*)	20-30% (*9*)	5.5% (*15*)
		Ki-67$^+$	1%	N/D	>1.5% (*11*)	N/D	N/D
In vitro GSIS	Static		6-12	2-3 (*13*)	N/D	3 (*9*)	1.2-1.6 (*15*)
	Dynamic	1st phase	16.1±6.29	3-9 (*12, 13*)	5-10 (*11*)	7.6±1.3 (*9*)	1.9±0.5 (*15*)
		2nd phase	6.1±1.07	2-3 (*12, 13*)	3-4 (*11*)	2.1±0.3 (*9*)	N/D
Transplantation studies	Transplantation site		ACE	Kidney (*12, 17, 18*)	Kidney (*11*), hind leg (*16*)	Kidney (*9*)	Kidney (*15*)
	Number of SC-islets or cells transplanted		600 SC-islets (1.2x10^6 cells)	2-5x10^6 cells (*12, 17, 18*)	250-750 SC-islets (*11*)	5x10^6 cells (*9*)	N/D
	Reversal of preexisting diabetes		Yes	Yes (*12, 18*)	N/D	Yes (*9*)	N/D
	IPGTT and *in vivo* GSIS		Yes	Yes (*12, 18*)	Yes (*11*)	Yes (*9*)	Yes (*15*)
	Pancreatic exocrine cells		Not detected	Yes (*17*)	Yes (*16*)	Yes (*9*)	Yes (*15*)

Results from the herein presented protocol (Wu *et al*) are compared to what have been reported from four published protocols. The yield shows number of S6-7 SC-islet cells generated per initial stem cell. The identities of the hPSC lines from which SC-islets have been successfully generated are as shown. The main cell lines used for these studies are marked with **bold** text. The differentiation efficiency is presented as percentages of PP cells at the end of S4, EP cells at the end of S5, and endocrine and proliferative cells at the end of S6-7, as determined by flow cytometry and immunohistochemistry. The results for *in vitro* static and dynamic GSIS assays are presented as fold change over the low glucose level. The presence of pancreatic exocrine cells in graft is determined by immunohistochemistry. Data are estimated from the results presented in this manuscript and the previous reports (see Supplemental References). N/D, data not available.

Table S2: Conjugated antibodies for analysis with flow cytometry.

Conjugated antibodies	Source	Identifier	Dilution
Alexa Fluor 647 mouse anti-Insulin	BD Biosciences	565689	1:20
PE mouse anti-Glucagon	BD Biosciences	565860	1:20
Alexa Fluor 488 mouse anti-human Somatostatin	BD Biosciences	566032	1:20
PE mouse anti-NEUROD1	BD Biosciences	563001	1:20
Alexa Fluor 647 mouse anti-NKX6.1	BD Biosciences	563338	1:20
Alexa Fluor 488 mouse anti-PDX-1	BD Biosciences	562274	1:20
Alexa Fluor 488 mouse anti-Ki-67	BD Biosciences	561165	1:20
V450 mouse anti-Ki-67	BD Biosciences	561281	1:20

Table S3: Primary antibodies for analysis with immunofluorescence.

Primary antibodies	Source	Identifier	Application	Dilution
goat anti-human PDX-1	R&D systems	AF2419	ICC	1:300
guinea pig anti-PDX1	abcam	ab47308	ICC	1:200
mouse anti-NKX6.1	DSHB	F55A12-s	ICC	1:100
goat anti-human/mouse NEUROD1	R&D systems	AF2746	ICC	1:100
sheep anti-human NGN3	R&D systems	AF3444	ICC	1:100
guinea pig anti-C-Peptide	abcam	ab30477	ICC	1:100
rat anti-C-Peptide	DSHB	GN-ID4-s	ICC	1:50
mouse anti-Glucagon	Sigma	G2654	ICC	1:1000
rabbit anti-Somatostatin	Sigma	332A-1	ICC	1:500
rabbit anti-SLC18A1	Sigma	HPA063797	ICC	1:500
Goat anti-human SOX9	R&D systems	AF3075	IHC	1:40
guinea pig anti-Insulin	Dako Agilent	IR00261-2	IHC	1:10
rabbit anti-Glucagon	BioGenex	PU039-5UP	IHC	1:300
rat anti-human Somatostatin	Bio-Rad	8330-0009	IHC	1:400

Supplemental Methods

hPSC culture

hESC line HS980 (KIe033-A) was derived under xeno-free, defined conditions with informed donor consent (Swedish Ethical Review Authority 2011/745:31/3)[1]. hESC line KARO1 (KIe034-A) was generated and banked under GMP guidelines[2]. WA01/H1 (WAe001-A) and WA09/H9 (WAe009-A) were purchased from WiCell. Human iPSC lines CTRL-7-II (C7), CTRL-9-II (C9), CTRL-12-I (C12), and CTRL-14-II (C14) were obtained from the Karolinska Institute iPSC core facility[3]. Mycoplasma testing was performed every 6 months, and pluripotency marker expression was confirmed by flow cytometry.

hPSCs were maintained in NutriStem hPSC XF Medium (Biological Industries, 05-100-1A) on culture plates coated with 10 µg/mL human recombinant laminin (LN) -521 (BioLamina, LN521). Cultures were kept at 37°C with 5% CO_2 and 5% O_2 and passaged every 3–5 days at a density of 15,000–24,000 cells/cm². For passaging, cells were washed with PBS (Thermo Fisher, 14190169), incubated with TrypLE Select (Thermo Fisher, A1285901) for 4–5 min at 37°C, gently dissociated, centrifuged at 300 g for 5 min, resuspended in fresh NutriStem, and re-plated onto newly coated plates.

In vitro pancreatic islet differentiation of hPSCs

The pancreatic islet differentiation protocols described here were modified from previously published protocols[4-10].

The hESC lines were seeded onto LN-521 coated cell culture plates at 18000 - 24000 cells/cm^2 in NutriStem hPSC XF medium. The human iPSC lines were seeded onto LN-521 coated cell culture plates at 15000 cells/cm^2 in NutriStem hPSC XF medium supplemented with 5 µM ROCK inhibitor Y-27632 (Bio-Techne, 1254). Next day, the medium was changed to NutriStem hPSC XF medium without Y-27632. The pancreatic differentiation was initiated four days later, resulting in 90-100% confluency. The differentiating cell cultures were maintained in a 37°C incubator with 5% CO_2, 20% O_2 and 100% humidity. The differentiation can be divided into six stages (S1-S6), and media used for each stage were as follows:

S1 media: MCDB131 (Thermo Fisher; 10372019) + 25 mM NaHCO3 (Sigma; S6297) + 1X GlutaMAX (Thermo Fisher; 35050038) + 50 U/ml Penicillin-Streptomycin (Thermo Fisher; 15140122) + 2.5 mM D-Glucose (8 mM final concentration, Sigma; G8769) + 0.2% or 0.5% Fatty Acid Free Bovine Serum Albumin (FAF-BSA, Sigma; A8806).

S2 media: MCDB131 + 25 mM NaHCO3 + 1X GlutaMAX + 50 U/ml Penicillin-Streptomycin + 2.5 mM D-Glucose (8 mM final concentration) + 0.2% or 0.5% FAF-BSA + 0.25 mM Vitamin C (Sigma; A4544).

S3-4 media: MCDB131 + 25 mM NaHCO3 + 1X GlutaMAX + 50 U/ml Penicillin-Streptomycin + 2.5 mM D-Glucose (8 mM final concentration) + 0.5% FAF-BSA + 0.25 mM Vitamin C + 1:200 ITS-X (Thermo Fisher; 51500056).

S5 media: MCDB131 + 25 mM NaHCO3 + 1X GlutaMAX + 50 U/ml Penicillin-Streptomycin + 14.5 mM D-Glucose (20 mM final concentration) + 0.5% FAF-BSA + 1:200 ITS-X + 10 µM ZnSO4 (Sigma; Z0251) + 10 µg/ml Heparin (Sigma; H3149).

S6 media: CMRL (Thermo Fisher; 11530037) + 14 mM NaHCO3 + 1X GlutaMAX + 50 U/ml Penicillin-Streptomycin + 14.5 mM D-Glucose (20 mM final concentration) + 1% FAF-BSA + 1:200 ITS-X (for three weeks) + 10 µM ZnSO4 + 10 µg/ml Heparin + 1X NEAA (Thermo Fisher; 11140035).

The short differentiation protocol:

S1 definitive endoderm (3 days): Undifferentiated hPSCs were rinsed once with D-PBS with Ca^{2+} and Mg^{2+} (Thermo Fisher; 14040091) and then induced with 5 µM CHIR99021 (Tocris; 4423) and 100 ng/ml Activin A (R&D; 338-AC) for 24 hours in S1 media. For the next 2 days the cells were fed every day with S1 media containing only 100 ng/ml Activin A. The concentrations of FAF-BSA in S1 media were 0.2% for HS980, C9, C12 and C14 cells, and 0.5% for H1, H9, KARO1, and C7 cells.

S2 primitive gut tube (3 days): cells were induced with 50 ng/ml KGF (R&D; 251-KG) in S2 media for 3 days. The concentrations of FAF-BSA were 0.2% for HS980, C9, C12 and C14 cells, and 0.5% for H1, H9, KARO1, and C7 cells.

S3 posterior foregut (1 day): cells were induced with 50 ng/ml KGF, 2 µM Retinoic acid (Sigma; R2625), 0.25 µM SANT-1 (Sigma; S4572), 0.5 µM PDBu (Tocris; 4153), and 200 nM LDN193189 (Tocris; 6053) in S3-4 media for 24 hours.

S4 pancreatic progenitor (3 days): cells were induced with 50 ng/ml KGF, 100 ng/ml EGF (R&D; 236-EG), 5 ng/ml Activin A, 10 mM Nicotinamide (Sigma; N0636), 100 nM Retinoic acid, 0.25 µM SANT-1, 0.5 µM PDBu, and 200 nM LDN193189 in S3-4 media for 3 days. To analyze the effect of stage 4 durations, cells were also differentiated for 1-5 days during this step.

S5 endocrine progenitor (5 days): cells were induced with 20 ng/ml Betacellulin (R&D; 261-CE), 100 nM Retinoic acid, 0.25 µM SANT-1, 100 nM GSI-XX (Sigma; 565789), 10 µM ALK5 inhibitor II (Cayman Chemical; 14794), 1 µM GC-1 (Tocris; 4554), and 100 nM LDN193189 in S5 media for 4 days.

Four days into S5 (S5d4), cells were rinsed once with PBS, treated with StemPro Accutase cell dissociation reagent (Thermo Fisher; A1110501) for 10 minutes at 37°C, and then dissociated into single cells in S5 media by pipetting 10-15 times using a P1000 pipette. Single cells were pelleted by centrifugation at 300 g for 5 minutes, and then resuspended at 1.0-1.5 x 10^6 cells/ml in S5 media supplemented with 10 µM H1152 (Tocris; 2414) and the other factors. To generate islet-like aggregates, cells were transferred to ultra-low attachment 6-well plates (Corning; 3471), totally 4-6 x 10^4 cells in 4 ml per well, and incubated overnight on an orbital shaker (Infors HT Celltron) at 95 rpm, Ø 25 mm, in the incubator.

To investigate the effects of ROCK inhibitor H1152 on cell survival and aggregate formation, different concentrations of H1152, from 0 to 10 µM, were added to a single cell suspension for 24 hours. Next day, the cell aggregates were collected, rinsed once in PBS, treated with Accutase for 10 minutes at 37°C, and then dissociated into single cells in S5 media by pipetting 10-15 times using a P1000 pipette. The cell number was counted to determine the aggregation efficiency using the equation:

Aggregation efficiency = Nf / Ni

where Ni is the initial number of single cells at S5d4 and Nf is the number of cells in aggregates at S5d5.

S6 pancreatic islets (about 4 weeks): the cell aggregates were maintained in S6 media further supplemented with 10 µM H1152, 1 µM GC-1, 10 µM Trolox (Merck Millipore; 648471), and 1 mM N-acetyl-L-cysteine (Sigma; A9165). ITS-X and H1152 were removed from the media after three weeks. The aggregates were kept on an orbital shaker at 95 rpm in the incubator.

The media were changed every day from stage 1 to 5, and every 2-3 days during stage 6.

The long differentiation protocol:

hPSCs were differentiated using the same factors and media as the short differentiation protocol described above. The durations for S3 and S4 were 2 and 5 days respectively.

Comparison to published differentiation protocols

To benchmark our LN-521-based short differentiation protocol, H1 cells were differentiated in parallel using published protocols from Velazco-Cruz *et al.*[9] and Balboa *et al.*[11], following the original procedures. Endocrine composition was assessed by flow cytometry (see Methods), and functional maturation by static GSIS (see below). In addition, Table S1 provides a structured overview of several published SC-islet differentiation protocols, including 2D/3D format, SC-islet yield, hPSC lines tested, differentiation efficiencies at stages S4–S6/7, static and dynamic GSIS, and transplantation outcomes, based on previously published literature[9,11-18].

Dissociation of SC-islet and counting of cell number

The S6 SC-islets were counted under a brightfield microscope. S6 SC-islets were rinsed in PBS and incubated with Accutase for 12–15 min on an orbital shaker (95 rpm, 37°C). Islets were dissociated to single cells by pipetting 15× with a P1000 pipette, resuspended in S6 medium, pelleted at 300 g for 5 min, and washed in PBS. Cell numbers were quantified using a MOXI Z Mini Automated Cell Counter (ORFLO, MXZ001).

Freezing and thawing of stage 5 endocrine progenitor cells

At S5d4, cells were dissociated to single cells, pelleted at 300 g for 5 min, and resuspended at 1×10^7 cells/mL in cold STEM-CELLBANKER GMP solution (Amsbio, 11924). Suspensions (1–1.5 mL) were aliquoted into Nunc cryogenic tubes (Thermo Fisher, 377267) and cooled to –80°C using a Mr. Frosty container (Thermo Fisher, 5100-0001). Alternatively, cells can be cooled at 1°C/min using a programmable cooling unit. For long-term storage, vials were transferred to liquid nitrogen.

Frozen S5 cells were retrieved from liquid nitrogen and rapidly thawed at 37°C. Each 1 mL suspension was diluted into 5 mL pre-warmed S5 medium, centrifuged at 300 g for 5 min, and resuspended at 1.0–1.5 × 10^6 cells/mL in complete S5 medium. Differentiation then proceeded as described above.

Static *in vitro* glucose stimulated insulin secretion (GSIS)

SC-islets (20–30 per assay) at S6w4 were incubated overnight in S6 medium lacking ITS-X and without additional glucose (final glucose concentration 5 mM). The next day, SC-islets were transferred to 24-well ultra-low attachment plates (Corning; 3473) and washed twice with 2 mL Krebs buffer containing 129 mM NaCl, 4.8 mM KCl, 2.5 mM $CaCl_2$, 1.2 mM $MgSO_4$, 1 mM Na_2HPO_4, 1.2 mM KH_2PO_4, 5 mM $NaHCO_3$, 10 mM HEPES, and 0.1% fatty acid–free BSA (FAF-BSA). Islets were pre-incubated in 2 mL Krebs buffer supplemented with 2 mM glucose for 2 hours to remove residual insulin.

SC-islets were then sequentially incubated for 30 min in 2 mL Krebs buffer under the following conditions:
(1) 2 mM glucose (low glucose), (2) 20 mM glucose (high glucose), (3) 2 mM glucose (low glucose), and (4) 2 mM glucose plus 30 mM KCl (depolarization). Between each incubation, islets were washed with 2 mL Krebs buffer. After each incubation, 500 µL supernatant was collected and stored for analysis.

Following the KCl challenge, SC-islets were dissociated with Accutase for 15 min and total cell numbers were determined using an ORFLO MOXI Z cell counter. Secreted insulin was quantified using a human C-peptide ELISA kit (R&D; DICP00). C-peptide

secretion was normalized to total cell number and reported as pmol C-peptide released per 1×10^3 cells or as fold change. Samples not analyzed on the same day were stored at −80°C.

Dynamic GSIS assay

Dynamic glucose-stimulated insulin secretion (GSIS) was performed using a Biorep PERI-4.2 perifusion system (Biorep Technologies). Fifty handpicked SC-islets were loaded into 0.27 ml columns containing Bio-Gel P4 polyacrylamide beads (Bio-Rad, 1504128). Perifusion buffer consisted of 129 mM NaCl, 4.8 mM KCl, 2.5 mM $CaCl_2$, 1.2 mM $MgSO_4$, 1 mM Na_2HPO_4, 1.2 mM KH_2PO_4, 5 mM $NaHCO_3$, 10 mM HEPES, and 0.1% FAF-BSA, supplemented with either 2.8 mM (low) or 16.8 mM (high) glucose. The buffer was delivered at a flow rate of 50 µl/min at 37°C. Following a 90-min pre-equilibration in 2.8 mM glucose, SC-islets were sequentially exposed to: 2.8 mM glucose for 12 min, 16.8 mM glucose for 36 min, and 2.8 mM glucose for 20 min. Effluent was collected every 2 min, and insulin concentrations were quantified using the AlphaLISA insulin detection kit (AL3184). A 6-min tubing delay was corrected for in data presentation.

Single-cell RNA sequencing sample preparation

S6 SC-islets from H1 and HS980 cells were collected on day 43 of differentiation. Islets were rinsed twice in PBS and incubated with TrypLE (15 min, 37°C, orbital shaker) to dissociate into single cells by pipetting. Cells were centrifuged at 300 × g for 5 min, resuspended in PBS + 0.04% BSA (Sigma, A7284), and filtered through a 40 µm strainer (VWR, 732-2760).

For post-transplant samples, mouse iris containing SC-islet grafts was excised at 20 weeks post-transplantation. Surrounding tissue was trimmed and washed twice in PBS. Samples were digested in PBS containing 2 mg/ml Collagenase IV (ThermoFisher, 17104019), 10 U/ml DNase I (Merck, 04716728001), and 1 mg/ml Papain (Sigma, 76216) for 50 min at 37°C in a ULA 6-well plate, with 15 pipetting strokes every 10 min. Enzymatic activity was quenched with PBS + 10% FBS. Cells were filtered (40 µm), pelleted, and resuspended at 0.8×10^6 cells/ml in PBS + 1% BSA. Viability (~87%) and total cell numbers were determined using Trypan blue (0.4%, ThermoFisher, 15250061) and a hemocytometer. For scRNA-seq, 5×10^3 H1, 2.5×10^3 HS980, and 1×10^4 graft-derived cells were loaded per library.

Single-cell RNA sequencing analysis

Single-cell libraries were prepared using 10x Genomics Chromium Next GEM Single Cell 3′ Reagent Kits v3.1 (CG000315 or CG000388), with optional Cell Multiplexing Oligo labeling (CG000391). Libraries were sequenced on an Illumina NextSeq 2000 at ~4×10^4 reads per cell. FASTQ files and feature-barcode matrices were generated using Cell Ranger 6.1.1 (*cellranger count*, default settings). Reads were aligned to the human reference genome GRCh38.98 (v3.0.0). To exclude mouse cells in graft samples,

reads were also mapped to the mm10 reference genome (gex-mm10-2020-A, 10x Genomics), and only cells expressing ≥2.25-fold more human than mouse genes (based on pre-transplant controls) were retained. Downstream analysis was performed in Seurat v5.1.0 (R v4.3.3), keeping high-quality cells with 1,000–7,000 detected genes (nFeature) and <25% mitochondrial genes (percent.mito), yielding 3,940 H1, 1,485 HS980, and 4,461 grafted cells.

Single-cell RNA-seq data from H1 SC-islets were quality-controlled, excluding mitochondrial genes, and analyzed using the standard Seurat pipeline[19]. Genes expressed in ≥5 cells were log-normalized using the computeSumFactors function in R scran package (v1.30.2)[20]. The 2,000 most variable genes were identified using the vst method with FindVariableFeatures function. The top 25 principal components (PC) were computed (RunPCA) and used for uniform manifold approximation and projection (UMAP) dimensional reduction (RunUMAP). Clusters were identified with default Louvain clustering (FindClusters). Cluster stability was assessed with the R package clustree v0.4.3[21] across resolution values 0.2–0.8, with 0.4 resolving delta and proliferating cells. Cell identities were assigned using canonical markers. Datasets from HS980 before and after transplantation were log-normalized, rescaled with the multiBatchNorm function in batchelor package (v1.18.1)[22] to normalize size factors, and integrated using the RunFastMNN function in SeuratWrappers package (v0.3.5) with 2,000 anchor features and 25 PCs. Clustering and UMAP were performed on the integrated PCs. Differential gene expression between mature and early beta cells was assessed using the two-sided Wilcoxon test (FindMarkers). Genes with Bonferroni-adjusted $P < 0.05$, $\log_2 FC > 0.25$, and expression in >25% of cells were considered differentially expressed (DEG). Functional enrichment of DEGs and gene set enrichment analysis (GSEA) of all genes were performed using the "enricher" and "fgsea" functions from the clusterProfiler package (v3.18.1)[23] and fgsea package(v1.32.2) (http://biorxiv.org/content/early/2016/06/20/060012.), with KEGG annotations from EnrichR database[24]. To compare datasets, integration was performed with fastMNN, and integrated (MNN) PCs were used to construct UMAP, identify neighbors, and define clusters with Seurat. β cell populations before and after transplantation were compared using average expression and a maturation signature calculated from gene INS, G6PC2, HOPX, UCN, IAPP, CPE, SIX3, BACE2, MAFA, and FXYD2. RNA velocity in HS980 and grafted cells was calculated with Python script velocyto.py from scvelo package (v0.1.25)[25,26] and projected onto UMAP using scv.pl.velocity_embedding_stream with default parameters.

Comparison to established pancreatic differentiation protocols

Datasets from Augsornworawat *et al*., 2020 (S6, GSE151117), Balboa *et al*., 2022 (S7w3 day 20, GSE167880), and Veres *et al*., 2019 (S6w4, GSE114412), and Rajaei *et al*., 2025 (EGAS50000000905) were analyzed, totaling ~3×10^4 cells. Data from Augsornworawat, Balboa, and Rajaei were generated using the 10x Genomics platform[11,15,17], while Veres used inDrops[27]. To compare these protocols, batch-to-batch correction with multiBatchNorm was applied, followed by integration with fastMNN using 2500 anchor features and top 25 PCs. Downstream analysis followed the workflow described above (FindClusters resolution = 0.6).

Proliferation scores (Fig. 4I) were computed using AddModuleScore with MKI67, CDK1, TOP2A, CCNB2, CCNA2, and PBK. β-cell maturation scores (Fig. 6D) were calculated using AddModuleScore with INS, G6PC2, HOPX, UCN3, IAPP, CPE, SIX3, BACE2, MAFA, and FXYD2.

Transplantation studies

All animal procedures were approved by Regional Ethical Committee at Karolinska Institutet. Six to seven week old NOD-scid gamma mice (NSG, Jackson Laboratories) were maintained under controlled temperature and humidity on a 12 hours light/dark cycle with ad libitum chow diet and water.

Diabetes was induced in 8 week old NSG mice by intraperitoneal (i.p.) injection of streptozotocin (STZ; Sigma, S0130) at 60 mg/kg body weight for four consecutive days. Non-fasting blood glucose was monitored throughout the study using an Accu-Chek blood glucose meter (Roche). Blood samples were collected from the tail vein monthly in Microvette CB 300 EDTA K2E tubes (Sarstedt, 16.444). Human and mouse c-peptide levels were quantified using species-specific ELISA kits (Crystal Chem, 80954 and 90050).

Transplantation of SC-islets into ACE

SC-islets differentiated from HS980 cells, 280–300 per eye, were transplanted into the ACE of STZ- treated diabetic mice. Mice were anesthetized with isoflurane and secured with stereotaxic head holder and eye holder. Under a stereo microscope (M80, Leica), the cornea was punctured with a 23G needle, and SC-islets were delivered into the ACE using a glass microcannula connected via polyethylene tubing to a 0.5 mL syringe (Hamilton, USA). Oculentum simplex (APL, Sweden) was applied to prevent corneal dryness and inflammation. Temgesic (Indivior, Ireland) was administered subcutaneously (s.c.) at 0.1 μg/g body weight to relieve postoperative pain. Transplanted diabetic mice received long-acting insulin (Insulatard Penfill, Novo Nordisk) at 0.05–0.15 IU s.c. from day 4-5 post-transplant for 45-56 days.

Intraperitoneal glucose tolerance test (IPGTT)

IPGTTs were performed on 5 hours fasted mice. Blood glucose was measured at 0 min, and 15, 30, 60, and 120 min after glucose load (3.5 g/kg body weight, i.p.). Blood for c-peptide quantification was collected at 0, 30, 60, and 120 min, and plasma stored at −80°C. C-peptide levels were measured as described above.

Supplemental References

1. Rodin S, Antonsson L, Niaudet C, et al. Clonal culturing of human embryonic stem cells on laminin-521/E-cadherin matrix in defined and xeno-free environment. *Nat Commun*. 2014;5:3195. doi:10.1038/ncomms4195

2. Main H, Hedenskog M, Acharya G, Hovatta O, Lanner F. Karolinska Institutet Human Embryonic Stem Cell Bank. *Stem Cell Res*. May 2020;45:101810. doi:10.1016/j.scr.2020.101810

3. Plaza Reyes A, Petrus-Reurer S, Padrell Sánchez S, et al. Identification of cell surface markers and establishment of monolayer differentiation to retinal pigment epithelial cells. *Nat Commun*. Mar 30 2020;11(1):1609. doi:10.1038/s41467-020-15326-5

4. D'Amour KA, Bang AG, Eliazer S, et al. Production of pancreatic hormone-expressing endocrine cells from human embryonic stem cells. *Nat Biotechnol*. Nov 2006;24(11):1392-401. doi:10.1038/nbt1259

5. Kroon E, Martinson LA, Kadoya K, et al. Pancreatic endoderm derived from human embryonic stem cells generates glucose-responsive insulin-secreting cells in vivo. *Nat Biotechnol*. Apr 2008;26(4):443-52. doi:10.1038/nbt1393

6. Rezania A, Bruin JE, Arora P, et al. Reversal of diabetes with insulin-producing cells derived in vitro from human pluripotent stem cells. *Nat Biotechnol*. Nov 2014;32(11):1121-33. doi:10.1038/nbt.3033

7. Pagliuca FW, Millman JR, Gürtler M, et al. Generation of functional human pancreatic β cells in vitro. *Cell*. Oct 2014;159(2):428-39. doi:10.1016/j.cell.2014.09.040

8. Nostro MC, Sarangi F, Yang C, et al. Efficient generation of NKX6-1+ pancreatic progenitors from multiple human pluripotent stem cell lines. *Stem Cell Reports*. Apr 2015;4(4):591-604. doi:10.1016/j.stemcr.2015.02.017

9. Velazco-Cruz L, Song J, Maxwell KG, et al. Acquisition of Dynamic Function in Human Stem Cell-Derived β Cells. *Stem Cell Reports*. 02 2019;12(2):351-365. doi:10.1016/j.stemcr.2018.12.012

10. Cogger KF, Sinha A, Sarangi F, et al. Glycoprotein 2 is a specific cell surface marker of human pancreatic progenitors. *Nat Commun*. 08 2017;8(1):331. doi:10.1038/s41467-017-00561-0

11. Balboa D, Barsby T, Lithovius V, et al. Functional, metabolic and transcriptional maturation of human pancreatic islets derived from stem cells. *Nat Biotechnol*. Jul 2022;40(7):1042-1055. doi:10.1038/s41587-022-01219-z

12. Hogrebe NJ, Augsornworawat P, Maxwell KG, Velazco-Cruz L, Millman JR. Targeting the cytoskeleton to direct pancreatic differentiation of human pluripotent stem cells. *Nat Biotechnol*. 04 2020;38(4):460-470. doi:10.1038/s41587-020-0430-6

13. Hogrebe NJ, Maxwell KG, Augsornworawat P, Millman JR. Generation of insulin-producing pancreatic β cells from multiple human stem cell lines. *Nat Protoc*. Sep 2021;16(9):4109-4143. doi:10.1038/s41596-021-00560-y

14. Barsby T, Ibrahim H, Lithovius V, et al. Differentiating functional human islet-like aggregates from pluripotent stem cells. *STAR Protoc*. Dec 16 2022;3(4):101711. doi:10.1016/j.xpro.2022.101711

15. Rajaei B, Garcia AM, Juksar J, et al. Clinically compliant enrichment of human pluripotent stem cell-derived islets. *Sci Transl Med*. Apr 02 2025;17(792):eadl4390. doi:10.1126/scitranslmed.adl4390

16. Lithovius V, Lahdenpohja S, Ibrahim H, et al. Non-invasive quantification of stem cell-derived islet graft size and composition. *Diabetologia*. Jun 14 2024;doi:10.1007/s00125-024-06194-5

17. Augsornworawat P, Maxwell KG, Velazco-Cruz L, Millman JR. Single-Cell Transcriptome Profiling Reveals β Cell Maturation in Stem Cell-Derived Islets after Transplantation. *Cell Rep*. Aug 25 2020;32(8):108067. doi:10.1016/j.celrep.2020.108067

18. Maxwell KG, Kim MH, Gale SE, Millman JR. Differential Function and Maturation of Human Stem Cell-Derived Islets After Transplantation. *Stem Cells Transl Med*. Mar 31 2022;11(3):322-331. doi:10.1093/stcltm/szab013

19. Hao Y, Hao S, Andersen-Nissen E, et al. Integrated analysis of multimodal single-cell data. *Cell*. Jun 24 2021;184(13):3573-3587.e29. doi:10.1016/j.cell.2021.04.048

20. Lun AT, McCarthy DJ, Marioni JC. A step-by-step workflow for low-level analysis of single-cell RNA-seq data with Bioconductor. *F1000Res*. 2016;5:2122. doi:10.12688/f1000research.9501.2

21. Zappia L, Oshlack A. Clustering trees: a visualization for evaluating clusterings at multiple resolutions. *Gigascience*. Jul 01 2018;7(7)doi:10.1093/gigascience/giy083

22. Haghverdi L, Lun ATL, Morgan MD, Marioni JC. Batch effects in single-cell RNA-sequencing data are corrected by matching mutual nearest neighbors. *Nat Biotechnol*. Jun 2018;36(5):421-427. doi:10.1038/nbt.4091

23. Yu G, Wang LG, Han Y, He QY. clusterProfiler: an R package for comparing biological themes among gene clusters. *OMICS*. May 2012;16(5):284-7. doi:10.1089/omi.2011.0118

24. Kuleshov MV, Jones MR, Rouillard AD, et al. Enrichr: a comprehensive gene set enrichment analysis web server 2016 update. *Nucleic Acids Res*. Jul 08 2016;44(W1):W90-7. doi:10.1093/nar/gkw377

25. La Manno G, Soldatov R, Zeisel A, et al. RNA velocity of single cells. *Nature*. Aug 2018;560(7719):494-498. doi:10.1038/s41586-018-0414-6

26. Bergen V, Lange M, Peidli S, Wolf FA, Theis FJ. Generalizing RNA velocity to transient cell states through dynamical modeling. *Nat Biotechnol*. Dec 2020;38(12):1408-1414. doi:10.1038/s41587-020-0591-3

27. Veres A, Faust AL, Bushnell HL, et al. Charting cellular identity during human in vitro β-cell differentiation. *Nature*. 05 2019;569(7756):368-373. doi:10.1038/s41586-019-1168-5
